# NET‐DNA Activates the ANXA2/TMEM215/BiP Axis to Promote Mitophagy‐Mediated Anoikis Resistance in Endometriosis

**DOI:** 10.1002/advs.75442

**Published:** 2026-04-27

**Authors:** Honglin Wang, Yanling Gou, Huiyan Zhang, Hongli Wang, Beidi Wang, Jinming Liu, Yingying Cao, Ruru Bai, Yuxin Zhao, Xu Han, Chao Feng, Xin Huang, Zongfeng Zhang

**Affiliations:** ^1^ Department of Obstetrics & Gynecology Second Affiliated Hospital of Harbin Medical University Harbin China

**Keywords:** anoikis resistance, endometriosis, MAMs, mitophagy, NET‐DNA

## Abstract

Endometriosis (EMs) features ectopic implantation of endometrial stromal cells (EESCs) and strong anoikis resistance, yet how inflammatory signals reprogram mitochondrial function remains unclear. Here, neutrophil extracellular traps (NETs), particularly their DNA scaffold (NET‐DNA), are identified as enriched in early lesions and associated with enhanced mitophagy and EESCs survival. In primary human EESCs, NET‐DNA suppresses anoikis, increases mitochondrial membrane potential, decreases reactive oxygen species, and enhances ER–mitochondria contacts. NET‐DNA directly binds Annexin A2 (ANXA2), promotes its redistribution from the plasma membrane to the cytoplasm, and independently upregulates the ER‐resident transmembrane protein TMEM215. TMEM215 facilitates formation of a Ca2+‐dependent ANXA2–TMEM215 complex, enhancing ER–mitochondria contacts and PINK1/Parkin‐mediated mitophagy. Silencing ANXA2 or TMEM215 disrupts mitophagy, impairs mitochondrial Ca2+ handling, reduces ER–mitochondria contacts, and restores anoikis sensitivity. Proteomic analysis identifies Binding Immunoglobulin Protein (BiP/GRP78) as a TMEM215‐interacting partner, and NET‐DNA promotes assembly of a TMEM215–ANXA2–BiP complex that reinforces mitochondrial quality control. In mouse EMs models, knockdown of TMEM215 or ANXA2 reduces lesion growth and partially reverses LPS‐associated progression. These findings reveal a mechanism linking inflammation to mitochondrial reprogramming in EMs and suggest a potential therapeutic target.

## Graphical abstract

NET‐DNA is enriched during early endometriotic lesion establishment and drives anoikis resistance by promoting an ANXA2–TMEM215–BiP axis. This pathway strengthens ER–mitochondria contacts, enhances PINK1/Parkin‐associated mitophagy, preserves mitochondrial homeostasis, and supports ectopic stromal cell survival, thereby facilitating lesion persistence and progression.

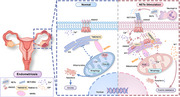

## 1 Introduction

Endometriosis (EMs) is a chronic, estrogen‐dependent inflammatory disorder characterized by the implantation and proliferation of endometrial‐like tissues outside the uterine cavity, predominantly involving the pelvic peritoneum and ovaries [1, 2]. It affects approximately 10% of reproductive‐aged women, manifesting as severe symptoms such as chronic pelvic pain, dysmenorrhea, dyspareunia, and infertility, significantly compromising their quality of life [3, 4]. Although Sampson's retrograde menstruation hypothesis, which proposes the transportation of viable endometrial cells through the fallopian tubes into the peritoneal cavity, is widely accepted [5], this phenomenon occurs in up to 90% of women, yet only a minority develop EMs. This discrepancy strongly indicates that additional factors are necessary for ectopic endometrial cell survival, attachment, and proliferation within the adverse peritoneal environment [6, 7, 8].

A critical prerequisite for the successful implantation of displaced endometrial cells is their ability to evade programmed cell death, particularly anoikis, a form of apoptosis triggered by the loss of cell‐matrix interactions [9, 10, 11]. Consequently, acquiring anoikis resistance is considered a pivotal event enabling endometrial cells to survive in suspension within the peritoneal fluid and subsequently establish ectopic lesions [12]. However, how endometrial stromal cells acquire anoikis resistance in the inflammatory peritoneal milieu remains poorly understood. Thus, elucidating these mechanisms could reveal novel therapeutic targets to prevent disease initiation and progression.

The peritoneal cavity in women with EMs is characterized by chronic sterile inflammation [13]. Increasing evidence highlights a critical role of innate immunity, particularly neutrophils, in driving this pro‐inflammatory microenvironment [14]. Upon activation, neutrophils release neutrophil extracellular traps (NETs), whose DNA scaffold and associated proteins act as potent inflammatory mediators [15]. Recent studies have demonstrated increased levels of NETs and their components, such as cell‐free DNA (NET‐DNA), in the peritoneal fluid and systemic circulation of patients with EMs, correlating with disease severity [16, 17]. Although NETs are recognized as modulators of inflammation and cellular processes in various pathological conditions, their precise role in promoting ectopic endometrial cell survival remains unexplored.

Therefore, in this study, we investigated whether NET‐DNA acts as an extracellular inflammatory signal that promotes anoikis resistance in EESCs. We further explored whether this effect is associated with altered mitophagy and ER–mitochondria communication. These findings may provide new insight into how inflammatory cues support ectopic lesion establishment and identify potential therapeutic targets for EMs.

## 2 Materials and Methods

### 2.1 Animal Experiments

#### 2.1.1 EMs Mouse Model

Female C57BL/6 mice (6–8 weeks old) were obtained from the Animal Center of Harbin Medical University and maintained under specific pathogen‐free (SPF) conditions. The EMs model was established following a previously described protocol [18]. Briefly, donor mice were treated intramuscularly with 17β‐estradiol‐3‐benzoate (30 µg/kg; Sigma, USA) [19, 20] for three consecutive days. Uterine horns were collected, minced into approximately 1 mm^3^ fragments, and intraperitoneally injected into two recipient mice (n = 6 per group) using an 18‐gauge needle to induce ectopic lesion formation. Four days after implantation, mice were randomized into three groups and treated daily by intraperitoneal injection with saline, lipopolysaccharide (LPS, 10 µg/mouse; MCE, China), or LPS combined with deoxyribonuclease I (DNase I, 100 U/mouse; Sigma, USA) for 14 consecutive days. On day 24, mice were sacrificed, and ectopic lesions were harvested. Lesion length and width were measured using a vernier caliper, and lesion volumes were calculated using the formula V = (length × width^2^) × 0.52. All animal procedures were approved by the Institutional Animal Care and Use Committee of Harbin Medical University.

#### 2.1.2 Peritoneal Lavage

In the experiment, we found that the data for days 1, 3, and 5 were insufficient because the lesions had not formed yet, or the lesions formed were too small to perform subsequent histological, immunofluorescence, and protein analysis (Figure S4). Therefore, the sample size at these early time points was limited, and could not provide sufficient support for the experiments. EMs mouse models were established and maintained for 7, 14, 21, and 28 days (n = 6 per time point) as described above. Peritoneal cavities of both healthy control and EMs mice at the indicated time points were lavaged with 1 mL phosphate‐buffered saline (PBS). The collected peritoneal lavage fluid was analyzed by flow cytometry to characterize immune cell subsets based on the established gating strategy.

#### 2.1.3 Tail Vein Injection

Twenty mice were randomly selected as donors and divided into two groups (n = 10 per group): a treatment group receiving tail vein injections of AAV9 vectors encoding mouse TMEM215 or ANXA2 shRNA (AAV9‐shTMEM215/ANXA2; Taitool, Shanghai), and a control group receiving AAV9 vectors encoding negative control shRNA (AAV9‐shCtrl; Taitool, Shanghai). On days 1 and 7, each mouse received 10 µL of virus (total viral load: 1 × 10^1^ ^1^ V.G./mouse) diluted in 200 µL saline via tail vein injection. Beginning on day 10, mice received intramuscular injections of 17β‐estradiol‐3‐benzoate for three consecutive days. On day 14, eight mice per group were randomly selected as donors, and their uterine tissues were transplanted to successfully establish EMs models in twelve recipient mice after four days. On day 18, the recipient mice were divided into four groups (n = 6 per group): AAV9‐shCtrl + saline, AAV9‐shCtrl + LPS, AAV9‐shTMEM215/ANXA2 + saline, and AAV9‐shTMEM215/ANXA2 + LPS. Mice received daily intraperitoneal injections for 10 consecutive days to ensure adequate drug absorption. On day 31, all mice were euthanized, and ectopic lesions were collected. Lesion dimensions were measured using a vernier caliper, and volumes were calculated using the ellipsoid formula: V = (length × width^2^) × 0.52.

All animal experiments were reviewed and approved by the Institutional Animal Care and Use Committee of the Second Affiliated Hospital of Harbin Medical University.

### 2.2 Hematoxylin and Eosin (H&E) Staining

At the conclusion of the experiment, animals were sacrificed, and EMs lesions were excised and rinsed with 10 mL cold PBS. Tissues were then fixed in 4% paraformaldehyde, embedded in optimum cutting temperature (OCT) compound, and sectioned at 5 µm thickness. To assess global histological alterations, sections were stained using an HE staining kit (Beijing Solarbio Science & Technology, China) according to the manufacturer's protocol.

### 2.3 Immunohistochemical Staining (IHC)

Tissues were fixed, embedded, and sectioned as described previously for IHC analysis. Following antigen retrieval, sections were incubated overnight at 4°C with primary antibodies (Table S1). After washing, sections were incubated with an HRP‐conjugated secondary antibody, and immunoreactivity was visualized using DAB chromogen (CWBIO, Beijing, China). Finally, sections were counterstained with hematoxylin. Images were captured, and staining intensity was analyzed using ImageJ software by investigators blinded to group assignment.

### 2.4 Immunofluorescence (IF)

For IF analysis, cells were seeded onto glass coverslips placed in 24‐well plates and cultured overnight to facilitate attachment. Cells were fixed in 4% paraformaldehyde (Solarbio, P1110) for 15 min at room temperature, permeabilized with 0.1% Triton X‐100 (Solarbio, 9002‐93‐1) for 10 min, and blocked with 3% bovine serum albumin (BSA; Sigma, A9418) for 1 h at room temperature. Cells were subsequently incubated with primary antibodies (Table S1) overnight at 4°C, followed by incubation with appropriate fluorophore‐conjugated secondary antibodies for 1 h at room temperature in the dark. Cell nuclei were counterstained with DAPI (Beyotime, China) according to the manufacturer's instructions. Fluorescence images were obtained using a fluorescence microscope (Nikon, Japan), and fluorescence intensities were quantified with ImageJ software (NIH, USA) independently by two investigators in a blinded manner.

### 2.5 Western Blot (WB)

Total cellular proteins were extracted using RIPA lysis buffer (P0013E; Beyotime, China), separated via SDS‐PAGE, and transferred onto PVDF membranes (EMD Millipore, Billerica, MA, USA). Membranes were blocked with 5% non‐fat milk in Tris‐buffered saline with Tween‐20 (TBST), incubated overnight at 4°C with primary antibodies, and subsequently incubated with corresponding secondary antibodies (Proteintech, USA). Detailed antibody information is provided in Table S1. Protein bands were visualized using enhanced chemiluminescence (ECL) solution (P0018S; Beyotime, China).

### 2.6 Measurement of Circulating NETs‐Related Markers

Serum samples from patients with EMs and healthy controls were collected and centrifuged at 3,000 × g for 10 min to remove cellular debris. Serum myeloperoxidase (MPO) concentrations were quantified using a Human Myeloperoxidase ELISA Kit (Cat. No. JLC_Y8543, Jingkang Bio, China) according to the manufacturer's instructions. Absorbance at 450 nm was measured using a microplate reader, and concentrations were calculated based on a standard curve. Serum cell‐free double‐stranded DNA (cf‐dsDNA) levels were quantified using a 1× dsDNA HS Assay Kit (Cat. No. 12642ES60, YEASEN, Shanghai, China) according to the manufacturer's instructions. Fluorescence was measured using a microplate reader with black plates at an excitation wavelength of 480 nm and an emission wavelength of 520 nm. A standard curve was generated using serial dilutions of the supplied dsDNA standard, and cf‐dsDNA concentrations were calculated accordingly. Serum MPO and cf‐dsDNA were jointly used as circulating NETs‐related markers.

### 2.7 Cell Culture

Primary ectopic endometrial stromal cells (EESCs) were isolated from ovarian endometriotic lesions of 21 patients and cultured according to the following method. Ectopic endometrial tissues were minced into fragments of approximately 1 mm × 1 mm and enzymatically digested using 4% type IV collagenase (Sigma, USA) at 37°C for 40–60 min. The resulting cell suspension was filtered to remove debris and epithelial cells, washed three times to eliminate erythrocytes, and resuspended in DMEM containing 15% fetal bovine serum (FBS; Biological Industries, Israel) and 1% penicillin–streptomycin (Gibco, USA). Cells were seeded onto culture dishes and maintained in an incubator (37°C, 5% CO_2_). Culture medium was replaced after cell attachment. EESCs were passaged using 0.25% trypsin–EDTA (Gibco, USA) upon reaching approximately 80% confluency. Primary cells were identified using immunofluorescence staining with antibodies against Vimentin (Proteintech, Cat# 60330‐1‐Ig) and E‐cadherin (Proteintech, Cat# 60902‐1‐Ig), showing strong Vimentin positivity and absence of E‐cadherin expression, confirming the mesenchymal phenotype and high purity of the isolated EESCs (Figure S3C). Unless otherwise specified, n in primary cell experiments represents independent samples derived from different patients, and the cultured EESCs were used between the 2nd and 5th generations.

### 2.8 Isolation and Culture of Human Neutrophils

Human neutrophils were isolated according to the following protocols. After obtaining informed consent, peripheral blood (5 mL) anticoagulated with EDTA was layered onto Polymorphprep (Axis‐Shield, Oslo, Norway) and centrifuged at 500 × g for 30 min. The neutrophil‐rich layer was collected, washed with PBS, and centrifuged (450 × g for 5 min). Red blood cells were lysed using RBC lysis buffer (Solarbio, Beijing, China). Purified neutrophils were resuspended in RPMI 1640 medium (Gibco, USA) supplemented with 1% HEPES buffer (Gibco, USA).

### 2.9 NETs Induction and Purification

Isolated neutrophils were incubated with 500 nm PMA (P8139; Sigma‐Aldrich) for 3 h. After incubation, the supernatant was removed, and neutrophils were gently washed three times with 2 mL chilled PBS. Subsequently, adherent NETs were collected by repeated pipetting using 1.5 mL chilled PBS and centrifuged at 1000 × g for 15 min. The resulting supernatant was collected, and DNA concentrations within NETs were quantified using spectrophotometry. To ensure adequate removal of PMA‐derived contaminants, a PMA‐only mock control without neutrophils was included. Western blot analysis showed that the PMA‐only mock control did not produce the downstream effects on mitophagy and anoikis resistance observed in the NETs group (Figure S5C).

### 2.10 NET‑DNA Purification and Biotinylation

NET‐DNA was fragmented into approximately 500‐bp lengths using sonication and purified using a MicroElute DNA Clean‐up Kit (D6296; OMEGA). To evaluate the purity of the NET‐DNA preparation, protein concentration was measured by BCA assay, residual protein contamination was assessed by silver staining, and histone H3 retention was examined by Western blot (Figure S5A–E). NanoDrop analysis showed that the A260/A280 ratio of the purified DNA samples was close to 1.8, which is within the standard range for DNA purity. Purified NET‐DNA was then biotinylated with a DNA labeling kit (89818; Thermo Fisher Scientific).

### 2.11 DNA Pull‐Down

Biotinylated NET‐DNA was incubated with cell membrane proteins at 4°C overnight in the presence of a protease inhibitor. The following day, the mixture was incubated with streptavidin–agarose beads (HY‐KO208, MCE) for 2 h. The beads were washed three times with IP washing buffer, and the protein–DNA complex was eluted with IP elution buffer. Subsequently, the eluted proteins were resolved by silver staining and analyzed via mass spectrometry.

### 2.12 Transwell Assay

Washed EESCs (3 × 10^5^/mL), resuspended in DMEM without FBS, were seeded into the 8 µm‐pore Transwell upper chamber (Corning, USA), while DMEM containing 15% FBS was added to the lower chamber. After 48 h of treatment identical to the cell proliferation assay, the chambers were washed, fixed, and stained. Membranes were imaged under a light microscope, and migratory EESCs on the lower surface were counted to quantify migration capacity.

### 2.13 Wound Healing Assay

EESCs were seeded into six‐well plates at 2 × 10^5^/well. Once a confluent monolayer formed, vertical scratches were created using 200 µL pipette tips, and detached cells were removed with sterile PBS (VivaCell, China). FBS‐free DMEM was added, and cells were treated as described for the proliferation assay. Scratch widths were photographed and measured at 0, 24, and 48 h. Wound closure rate (%) = (Scratch distance at 0 h − Scratch distance at 48 h)/Scratch distance at 0 h.

### 2.14 5‐Ethynyl‐2’‐Deoxyuridine (EdU) Assay

EESCs proliferation was assessed using the EdU assay. EESCs were seeded in 24‐well plates (2 × 10^4^/mL) and assigned to various treatment groups after attachment, with three replicate wells per group. Treatments were performed as in the CCK‐8 assay. After 48 h, the EdU assay was conducted using an EdU Cell Proliferation Kit with Alexa Fluor 488 (Beyotime, China). Following incubation with EdU working reagent at 37°C for 4 h, cells were fixed, washed, and permeabilized. They were then incubated with the Click Additive Solution and Hoechst nuclear dye, protected from light. EdU incorporation rate (%) = EdU‐positive cells (green)/Hoechst‐positive cells (blue). Experiments were performed in triplicate.

### 2.15 Autophagic Flux Measurement (mCherry‐EGFP‐LC3B)

The tandem fluorescent mRFP‐GFP‐LC3B adenovirus (Ad‐LC3; Hanbio Biotechnology, China) was used to monitor autophagic flux in EESCs. As described previously [21], cells were cultured to ∼80% confluence before infection. At a multiplicity of infection (MOI) of 300, Ad‐LC3 produced optimal transduction with minimal cytotoxicity. After 48 h, cells were imaged using a confocal laser scanning microscope. Because GFP fluorescence is quenched in acidic lysosomes while mRFP remains stable, puncta with distinct signals reflect different autophagic stages: yellow puncta (mRFP^+^/GFP^+^) indicate autophagosomes, and red puncta (mRFP^+^/GFP^−^) represent autolysosomes.

### 2.16 Organelle Co‐Staining With ER‐Tracker, MitoTracker, and LysoTracker

LysoTracker Red (Beyotime, China), ER‐Tracker (Beyotime, China), and MitoTracker Green (Thermo Fisher, USA) were used to stain lysosomes, endoplasmic reticulum (ER), and mitochondria, respectively, according to the manufacturers’ instructions. Nuclei were counterstained with Hoechst 33342 (Beyotime, China). Cells were then imaged using a confocal microscope (Nikon Eclipse Ti; Nikon, Japan).

### 2.17 Intracellular Calcium Detection

Cytosolic and mitochondrial Ca^2+^ levels in EESCs were examined using Fluo‐4 AM (2 mm; Beyotime, China) and Rhod‐2 AM (5 mm; MCE, USA), respectively. Fluo‐4 AM was diluted in PBS to 0.5–5 µm, and cells were incubated at 37°C for 30 min, followed by washing and an additional 20–30 min incubation. For Rhod‐2 AM, cells were incubated with a 5–10 µm working solution at room temperature for 30 min and washed twice with PBS. Fluorescence was observed using a confocal laser scanning microscope (Nikon, Japan) with excitation/emission wavelengths of 488/525 nm for Fluo‐4 AM and 549/578 nm for Rhod‐2 AM.

### 2.18 Measurement of Mitochondrial Membrane Potential (Ψm, JC‐1 Staining)

The JC‐1 staining working solution (Solarbio, China) was added to a 6‐well plate and incubated at 37°C in a cell incubator for 30 min. After incubation, the supernatant was removed, and cells were washed twice with JC‐1 Buffer. Images were captured using a fluorescence microscope at excitation and emission wavelengths of 490 and 525 nm, respectively. JC‐1 aggregates present in healthy mitochondria emitted red fluorescence, whereas JC‐1 monomers present in damaged mitochondria emitted green fluorescence.

### 2.19 Analysis of Mitochondrial ROS

To evaluate mitochondrial ROS, cells were incubated with 5 µm MitoSOX Red (Thermo Fisher, USA) for 15 min. Fluorescence images were captured using a microscope at 555 nm excitation.

### 2.20 RNA Extraction and Quantitative Real‐Time PCR (RT–qPCR)

Total RNA was extracted from frozen tissues or cultured cells using TRIzol reagent (Invitrogen, USA), following the manufacturer's instructions. First‐strand cDNA synthesis was performed using 500 ng of total RNA and a cDNA synthesis kit (TransGen Biotech, China). Quantitative real‐time PCR (qRT‐PCR) was conducted using the Top Green qPCR SuperMix kit (TransGen Biotech, China) and a real‐time PCR system (Toyobo, Japan). Primers were synthesized by GENEWIZ (China) (Table S1). Gene expression levels were normalized to GAPDH mRNA expression, and relative mRNA expression was calculated using the 2^−ΔΔCt^ method.

### 2.21 Construction of Anoikis and Anoikis Resistance Models

EESCs (1 × 10^6^ cells/well) were seeded in ultra‐low attachment 6‐well plates (Corning, USA) to establish anoikis and anoikis resistance models. For the anoikis model, cells were cultured under suspension conditions for 24 h, after which suspended cells were collected for subsequent analyses. For the anoikis resistance model, cells were maintained in suspension for 7 days, with medium replaced every two days. Surviving suspended cells were reseeded onto standard culture dishes; those that successfully reattached and proliferated were defined as anoikis‐resistant EESCs.

### 2.22 Co‑Immunoprecipitation (Co‑IP)

Cell proteins were extracted using IP lysis buffer (Beyotime, China) supplemented with PMSF and a protease inhibitor cocktail. After centrifugation (14 000 × g, 15 min, 4°C), the supernatant was collected, and 5 µg of corresponding primary antibody was added according to the manufacturer's guidelines. The mixture was incubated overnight at 4°C on a rotating mixer. Subsequently, protein–antibody complexes were incubated with pretreated Protein A/G Plus agarose beads (MCE, China) for 4 h at 4°C with gentle rotation. Complexes were then collected by centrifugation, washed three times with precooled IP buffer, and eluted by resuspending beads in a mixture of loading buffer and lysis buffer, followed by boiling for 10 min. The eluted complexes were subsequently analyzed by immunoblotting or liquid chromatography–tandem mass spectrometry (LC‐MS/MS).

### 2.23 Proximity Ligation Assay (PLA)

PLA was performed using the NaveniFlex Cell MR Kit (Cat. No. NC.MR.100, Navinci Diagnostics AB, Uppsala, Sweden), according to the manufacturer's instructions. EESCs cultured on glass coverslips were fixed with 4% paraformaldehyde for 15 min and permeabilized with 0.2% Triton X‐100 for 10 min at room temperature. After blocking for 1 h at 37°C, the cells were incubated overnight at 4°C with primary antibodies from different species. Subsequently, Navenibody M1 and Navenibody R2 were added and incubated for 1 h at 37°C, followed by washes and incubation with ligation and amplification enzyme mixtures provided in the kit. After nuclear counterstaining with DAPI, coverslips were mounted using antifade mounting medium. Fluorescence signals representing protein–protein interactions were visualized using a confocal microscope (Nikon, Japan) equipped with DAPI and Cy5 filters.

### 2.24 Silver Staining

Following DNA pull‐down, membrane‑associated proteins bound to NET‑DNA were separated by SDS–PAGE and visualized using a Fast Silver Stain Kit (P0017S, Beyotime Biotechnology, Shanghai, China). Gels were fixed in 40% ethanol and 10% acetic acid for 30 min, rinsed with 30% ethanol, sensitized with sodium thiosulfate and sodium acetate for 30 min, and washed with deionized water. Gels were incubated in a silver nitrate solution with formaldehyde for 20 min, rinsed briefly, and developed in sodium carbonate, formaldehyde, and sodium thiosulfate until the desired band intensity appeared. The reaction was stopped with 0.5 m EDTA and 5% acetic acid. Silver‑stained gel bands were excised and analyzed by LC–MS/MS at Aopeen Biotechnology (Guangzhou, China).

### 2.25 Membrane and Cytosol Protein Extraction Kit

Membrane fractions were isolated from cell pellets using the Membrane and Cytosol Protein Extraction Kit (Beyotime Biotechnology, Shanghai, China) and the Pierce Cell Surface Protein Isolation Kit (A44390, Thermo Fisher Scientific). Cell pellets were resuspended in Reagent A containing PMSF and incubated on ice for 10–15 min. Cells were homogenized on ice using a Dounce homogenizer 30–50 times and centrifuged at 700 g for 10 min at 4°C to remove nuclei and intact cells. The supernatant was centrifuged at 14 000 g for 30 min at 4°C to pellet membranes. Pellets were resuspended in Reagent B, vortexed for 5 s, and incubated on ice for 5–10 min. After centrifugation at 14 000 g for 5 min at 4°C, the supernatant was collected as the membrane protein fraction. Membrane proteins were used for downstream assays, including DNA pulldown and silver staining.

### 2.26 Flow Cytometry

#### 2.26.1 Flow Cytometric Analysis of Apoptosis

After treatment, EESCs were washed with PBS and digested with trypsin (with or without EDTA) at room temperature. Digestion was stopped when cells detached with gentle pipetting. Cells were collected, centrifuged at 1000 × g for 5 min, washed, and counted. 5–10 × 10^4^ cells were resuspended in 195 µL Annexin V‑FITC buffer, followed by 5 µL Annexin V‑FITC and 10 µL PI (Beyotime, Cat. No. C1062M, Shanghai, China). Cells were mixed gently and incubated in the dark for 10–20 min, then kept on ice. Flow cytometry was performed immediately. Annexin V‑FITC emitted green fluorescence, and PI emitted red fluorescence. Data were analyzed using FlowJo.

#### 2.26.2 Flow Cytometric Sorting

After peritoneal lavage and counting, 2 × 10^6^ cells per sample were collected and stained with Aqua Live/Dead dye (Life Technologies, USA). Cells were incubated with anti‑mouse CD16/32 (Elabscience, China) to block Fc receptor binding. Cells were then stained with a standard antibody panel for 30 min at room temperature (details in Table S1). After staining, cells were washed with PBS and fixed with 0.4% paraformaldehyde. Data were acquired using a BD LSRII cytometer with BD FACSDiva, and compensation was performed at the start of each experiment. Data were analyzed using FlowJo v10 (TreeStar, USA).

### 2.27 Ethical Statement

This study involving human tissue samples was approved by the Ethics Committee of the Second Affiliated Hospital of Harbin Medical University (Approval No. YJSKY2024‐495). All animal experimental procedures were approved by the same institution (Approval No. YJSDW2024‐265) and were conducted in strict accordance with the Animal Research: Reporting of In Vivo Experiments (ARRIVE) guidelines and institutional regulations for the care and use of laboratory animals.

### 2.28 Clinical Samples

This study included 21 patients diagnosed with EMs by laparoscopy and histopathology at the Second Affiliated Hospital of Harbin Medical University between October 2023 and August 2025. Normal endometrial tissues were collected from 15 patients with other benign gynecological diseases, without a history of EMs or adenomyosis (Table 1). Inclusion criteria for EMs included patients with an intraoperative diagnosis of ovarian‐type EMs, confirmed by the EMs diagnostic guidelines and histopathologically verified postoperatively, as well as women with normal ovulation and regular menstrual cycles who had not received steroid hormone treatment in the past 6 months. Exclusion criteria for EMs included steroid hormone use within 6 months prior to surgery, gynecological malignancies, concurrent systemic malignancies or benign tumors, autoimmune diseases, infertility, and pregnant or lactating women. Pain‐related phenotypes, including dysmenorrhea, were not systematically recorded and therefore were not included in the present analysis. Based on medical history and clinical records, all samples were collected outside the menstrual phase. The study was approved, and written informed consent was obtained from all participants.

**TABLE 1:** Clinical characteristics of control women and patients with endometriosis.

	Control (n = 15)	Endometriosis (n = 21)	P value
Age (years)	33.73 ± 6.61	37.14 ± 7.07	0.099
BMI (kg/m^2^)	23.37 ± 4.12	23.44 ± 0.56	0.923
CA125 level (U/mL)	18.68 ± 12.46	64.42 ± 80.15	0.036
Menstrual average cycle (days)	29.35 ± 1.25	29.83 ± 2.25	0.458
Menstrual duration (days)	5.34 ± 1.28	5.88 ± 1.36	0.237
r‐AFS stage			
Stage I (minimal)		0/21	
Stage II (mild)		4/21	
Stage III (moderate)		10/21	
Stage IV (severe)		7/21	

### 2.29 Cell Transfection

Small interfering RNAs targeting TMEM215 (siTMEM215‑2, siTMEM215‑3), ANXA2 (siANXA2‑1, siANXA2‑3), and BiP (siBiP‑1, siBiP‑3), as well as the si‑NC control, were synthesized by Hanbio Biotechnology (China). The ANXA2 overexpression plasmid (ANXA2‑OE‑FLAG), its truncated mutants [I: del1–31aa; II: del32–104aa; III: del105–175aa; IV: del191–265aa; V: del266–339aa], the TMEM215‑OE‑HA plasmid, the BiP‑OE‑His plasmid, and corresponding empty vectors (Con‑FLAG, Con‑HA, Con‑His) were synthesized by GENE Biotechnology (China). EESCs were seeded in 96‑well or 6‑well plates and transfected at ∼70% confluence. siRNAs were diluted in Opti‑MEM (Gibco, USA). DNA and P3000 reagent (Invitrogen, USA) were diluted separately in Opti‑MEM. Lipofectamine 3000 (Invitrogen, USA) was diluted in Opti‑MEM, mixed with DNA–P3000, and incubated for 20 min. The mixture was added to plates, and the medium was replaced with fresh complete medium after 4–6 h.

### 2.30 Transmission Electron Microscopy (TEM)

Samples were fixed with 3% glutaraldehyde, post‐fixed with 1% osmium tetroxide, dehydrated in graded acetone solutions, and embedded in Epon 812 resin. Ultrathin sections were prepared using a diamond knife, stained with uranyl acetate and lead citrate, and examined using a transmission electron microscope (JEM‐1400‐FLASH, JEOL, Japan). TEM images were analyzed to assess mitochondrial morphology, mitophagic structures, and the integrity of mitochondria–endoplasmic reticulum contacts (MERCs). MERCs were quantified as the proportion of ER closely apposed (≤30 nm) to mitochondria relative to the total ER perimeter.

### 2.31 Statistical Analysis

All in vitro experiments were performed in three independent biological replicates. For experiments using primary human EESCs, each replicate represented an independent patient‐derived cell preparation. Statistical analyses were conducted using GraphPad Prism, and data are presented as mean ± SD or median with interquartile range, as appropriate. For non‐normally distributed data between two groups, the Mann–Whitney U test was used. Comparisons between two groups were performed using unpaired Student's t‐tests, while differences among multiple groups were analyzed using one‐way or two‐way ANOVA, followed by Bonferroni's post‐hoc multiple comparison tests. Spearman correlation analysis was used to calculate the r and p values for data with abnormal distribution. Pearson correlation coefficients (R values) were used to assess spatial colocalization between markers, with higher R values indicating stronger colocalization. Non‐significant differences were designated as “ns” (p ≥ 0.05), whereas *p < 0.05, **p < 0.01, ***p < 0.001, and ****p < 0.0001 indicated statistical significance.

## 3 Results

### 3.1 Abundant Infiltration of NETs in Endometriotic Lesions

**FIGURE 1:**
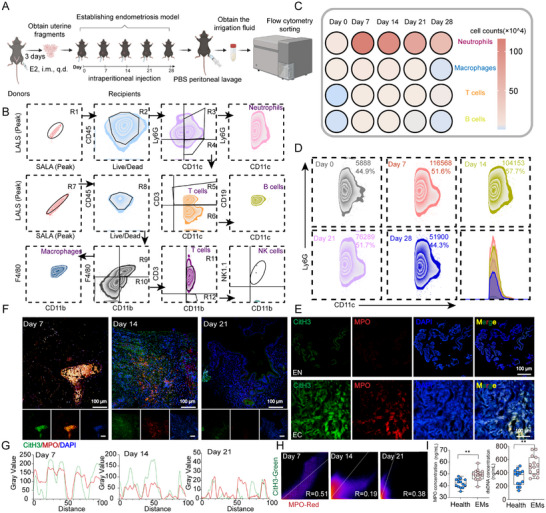
Abundant NETs infiltration in endometriotic lesions. (A) Experimental strategy for the establishment of the mouse model of endometriosis, including the collection of peritoneal lavage fluid and immune cell analysis. The schematic was created with BioRender.com. (B) Representative gating strategy for identifying neutrophils, macrophages, T cells, B cells, and NK cells in the peritoneal lavage fluid of mice with endometriosis by flow cytometry. (C) Dynamic changes in the composition of peritoneal immune cells in normal mice and mice at days 7, 14, 21, and 28 after induction of endometriosis (n = 6 per group). (D) Representative flow cytometry plots illustrating dynamic neutrophil frequencies, peaking at day 14. (E) Representative IF images showing MPO (red) and CitH3 (green) expression in normal (EN) and ectopic endometrium (EC). (F) Immunofluorescence staining of lesion tissues collected from mice at days 7, 14, and 21 after induction of endometriosis, showing the colocalization of MPO and CitH3, indicating NETs formation. (G) Line‐scan fluorescence intensity profiles confirming spatial colocalization of MPO and CitH3 at different time points. (H) Pearson's correlation‐based colocalization analysis depicting R values for MPO–CitH3 overlap in lesion on days 7, 14, and 21. (I) Serum MPO and cf‐dsDNA levels in healthy women (n = 14) and patients with endometriosis (n = 15). Serum MPO concentrations were measured by ELISA, and serum cell‐free double‐stranded DNA (cf‐dsDNA) levels were quantified using a fluorescence‐based dsDNA HS assay (Mann–Whitney U test). Data are expressed as mean ± SD. *P < 0.05.

To validate the roles of neutrophils and NETs in EMs, an EMs mouse model was established using 8‐week‐old female C57BL/6 mice (Figure 1A, created in BioRender.com). After model induction, peritoneal lavage fluid was collected at the early (days 7–14), middle (days 14–21), and late (days 21–28) disease stages. Flow cytometry sorting was used to analyze immune cell subpopulations at different stages of EMs progression (Figure 1B). The analysis demonstrated that neutrophils predominated in the early and middle stages, whereas in the late stage, both neutrophils and macrophages declined, accompanied by increased T cells (Figure 1C). Further quantification of neutrophils within upstream immune cell populations showed that neutrophils peaked on day 14 and gradually decreased thereafter (Figure 1D), indicating that neutrophils are prominently involved during the early phase of lesion development.

In human samples, immunofluorescence (IF) staining demonstrated stronger signals for MPO and CitH3 in ectopic endometrium (EC) tissue from EMs patients compared to normal endometrium (EN) tissue from healthy controls (Figure 1E). Based on these observations, lesion tissues from the EMs mouse model were collected at different disease stages, and frozen sections were prepared for IF staining to assess NETs infiltration. Consistent with results from peritoneal lavage fluid, NETs‐associated signals were significantly increased in lesion tissues during the early disease phase (days 7–14) compared with middle and late stages, as shown by co‐localization of the NET markers myeloperoxidase (MPO) and citrullinated histone H3 (CitH3) (Figure 1F,G). Quantitative analysis further showed that the colocalization coefficient (R value) of MPO and CitH3 peaked on day 7, whereas the value on day 14 was lower than that on day 21 (Figure 1H). Although the R value decreased on day 14 and remained lower than that on day 21, MPO–CitH3 colocalization was still detectable at all examined time points. Consistently, serum analysis revealed that both MPO and cell‐free double‐stranded DNA (cf‐dsDNA) levels were significantly elevated in patients with EMs (n = 15) compared with healthy controls (n = 14) (Figure 1I). Western blot (WB) analysis further confirmed elevated expression of MPO, CitH3, and neutrophil elastase (NE) in EC tissues compared to EN tissues (Figure S1A,B).

Collectively, these results show that NETs‐associated signals are elevated during the early stage of EMs lesion development, supporting a potential involvement of NETs in early lesion progression.

### 3.2 NETs Mediate Anoikis Resistance in EESCs Through NET‐DNA

**FIGURE 2:**
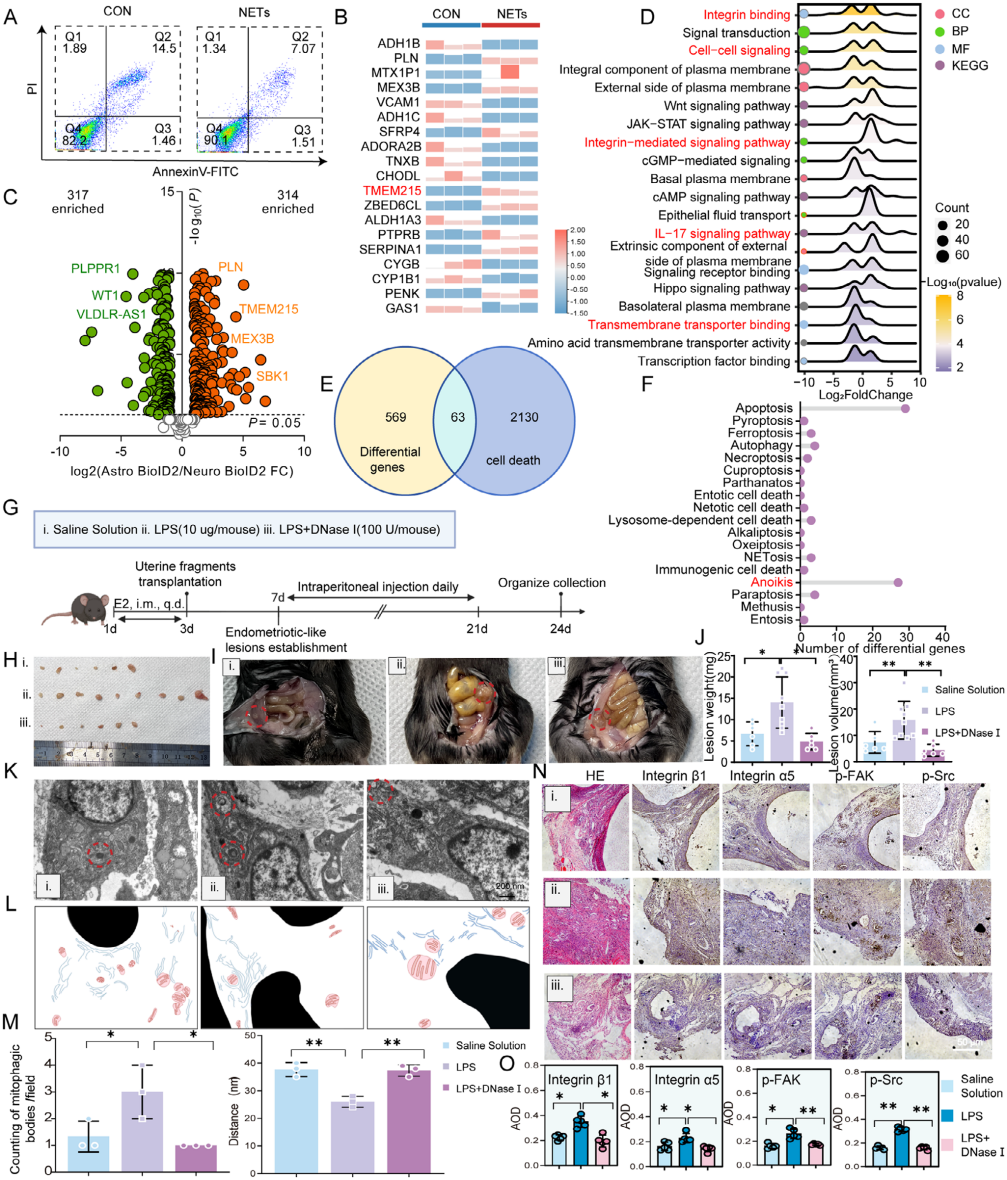
NETs promote anoikis resistance in EESCs through NET‐DNA. (A) Flow cytometry analysis of apoptosis in primary EESCs treated with control medium or NETs, as determined by PI/Annexin V staining. (B,C) Heatmap (B) and volcano plot (C) of differentially expressed genes in EESCs following NETs stimulation.(500 µg/mL, 48 h). (D) KEGG and GO enrichment analyses of the differentially expressed genes, showing enrichment in pathways related to integrin binding, cell‐cell signaling, integrin‐mediated signaling, IL‐17 signaling, and transmembrane transporter binding. (E) Venn diagram identifying 63 programmed cell death (PCD)‐related genes altered by NETs, prominently enriched for anoikis‐associated genes. (F) Distribution of genes associated with different types of cell death among the overlapping genes identified from the intersection of differentially expressed genes and cell death‐related genes, showing prominent enrichment of anoikis‐related genes. (G) Experimental design schematic for in vivo treatments involving daily intraperitoneal injections of saline, LPS (NET inducer), or LPS + DNase I (NET‐DNA degradation), and tissue collection. The schematic was created with BioRender.com. (H,I) Representative gross images of endometriotic lesions (H) and representative images of lesion establishment in mice (I) from the saline, LPS, and LPS + DNase I groups. (J) Quantification of lesion number, volume, and weight (n = 6 per group; one‐way ANOVA followed by Bonferroni test). (K,L) Representative transmission electron microscopy images of lesion tissues (K) and redrawn schematic images of mitochondria and endoplasmic reticulum based on the TEM images (L), which were used to quantify the distance between mitochondria and endoplasmic reticulum, in the saline, LPS, and LPS + DNase I groups. Red dashed circles indicate representative mitophagic structures. Scale bar = 200 nm. (M) Quantification of the number of mitophagic bodies per field and the distance between mitochondria and endoplasmic reticulum in the saline, LPS, and LPS + DNase I groups (n = 3 per group; one‐way ANOVA followed by Bonferroni test). (N,O) Representative HE staining and immunohistochemical staining of Integrin β1, Integrin α5, p‐FAK, and p‐Src (N), and corresponding AOD quantification (O) in lesion tissues from the saline, LPS, and LPS + DNase I groups (n = 6 per group; one‐way ANOVA followed by Bonferroni test). Scale bar = 50 µm. Data are expressed as mean ± SD. *P < 0.05; **P < 0.01.

To investigate the mechanistic role of NETs in the pathogenesis of EMs, neutrophils were isolated from the peripheral blood of healthy volunteers and induced in vitro to form NETs. These NETs were then applied at various concentrations to primary EESCs (Figure S1C). EdU staining, Transwell assays, wound‐healing assays, and PI/Annexin V staining were performed to evaluate cell proliferation, migration, and apoptosis (Figure S1D–F). NETs treatment significantly reduced apoptosis while promoting proliferation and migration of EESCs (Figure 2A), indicating that NETs are associated with enhanced survival‐related phenotypes in EESCs. To further explore the molecular mechanisms involved, EESCs were cultured for 48 hours with or without NETs (500 µg/mL), followed by RNA‐seq analysis. PCA showed a clear transcriptional distinction between the two groups (Figure S1G). Using criteria of P < 0.05 and |log_2_FC| > 1.5, 314 upregulated and 317 downregulated genes were identified, including multiple genes involved in cell adhesion and survival regulation (Figure 2B,C). KEGG and GO enrichment analyses of the differentially expressed genes indicated that these genes were enriched in pathways related to integrin binding, cell‐cell signaling, integrin‐mediated signaling, IL‐17 signaling, and transmembrane transporter binding (Figure 2D). To intersect these differentially expressed genes with programmed cell death (PCD)–related gene sets, we compared them with the Gene Ontology database (http://www.geneontology.org/). This analysis identified 63 significantly altered PCD‐related genes, among which anoikis‐associated genes were particularly prominent (Figure 2E,F). These findings support a potential role of NETs in regulating anoikis‐related pathways through cell adhesion and survival signaling. Since NETs consist of DNA scaffolds coated with proteases and cytokines, we next investigated which component mediated these observed effects. Purified NET‐DNA (5 ng/µL) alone significantly recapitulated the pro‐proliferative and pro‐migratory effects induced by intact NETs (Figure S1D–F), supporting NET‐DNA as an important effector component. To validate these results in vivo, an EMs mouse model was established and treated daily via intraperitoneal injection with saline, LPS (10 µg/mouse, a NET inducer), or LPS combined with DNase I (100 U/mouse, NET‐DNA degradation) for 14 days (Figure 2G, created in BioRender.com). Compared to saline‐treated mice, those administered LPS showed significantly increased lesion volumes and weights, while DNase I treatment markedly mitigated these effects (Figure 2H–J). Transmission electron microscopy (TEM) was employed to assess ultrastructural alterations in lesion tissues. As shown in Figure 2K, LPS‐induced lesions displayed increased autophagosome formation (highlighted by red dashed circles) and reduced mitochondria‐ER contact (MAM), which was further quantified in the schematic (Figure 2L). TEM quantification of the distance between mitochondria and the endoplasmic reticulum (ER) revealed significant alterations in the LPS and LPS + DNase I groups (Figure 2M), suggesting that NET‐DNA is associated with changes in mitophagy‐related ultrastructure and mitochondria‐ER interactions. To further characterize pathological alterations, histomorphological and IHC evaluations were performed. H&E staining revealed that lesions from the LPS group had pronounced tissue edema, disrupted glandular architecture, and stromal hyperplasia, all of which were notably alleviated by DNase I treatment. Immunohistochemistry (IHC) indicated that LPS administration enhanced local NETs infiltration and increased expression of several anoikis‐resistance markers, including integrin β1, integrin α5, p‐FAK, and p‐SRC. These LPS‐induced changes were reversed by DNase I, specifically showing a reduction in NETs infiltration and a significant decrease in the expression levels of anoikis resistance markers (Figure 2N,O). These findings demonstrate that NETs, at least in part through NET‐DNA, are associated with anoikis resistance in EESCs and may contribute to the early establishment and progression of endometriotic lesions.

### 3.3 NET‐DNA Enhances Anoikis Resistance of EESCs by Maintaining Mitophagy

**FIGURE 3:**
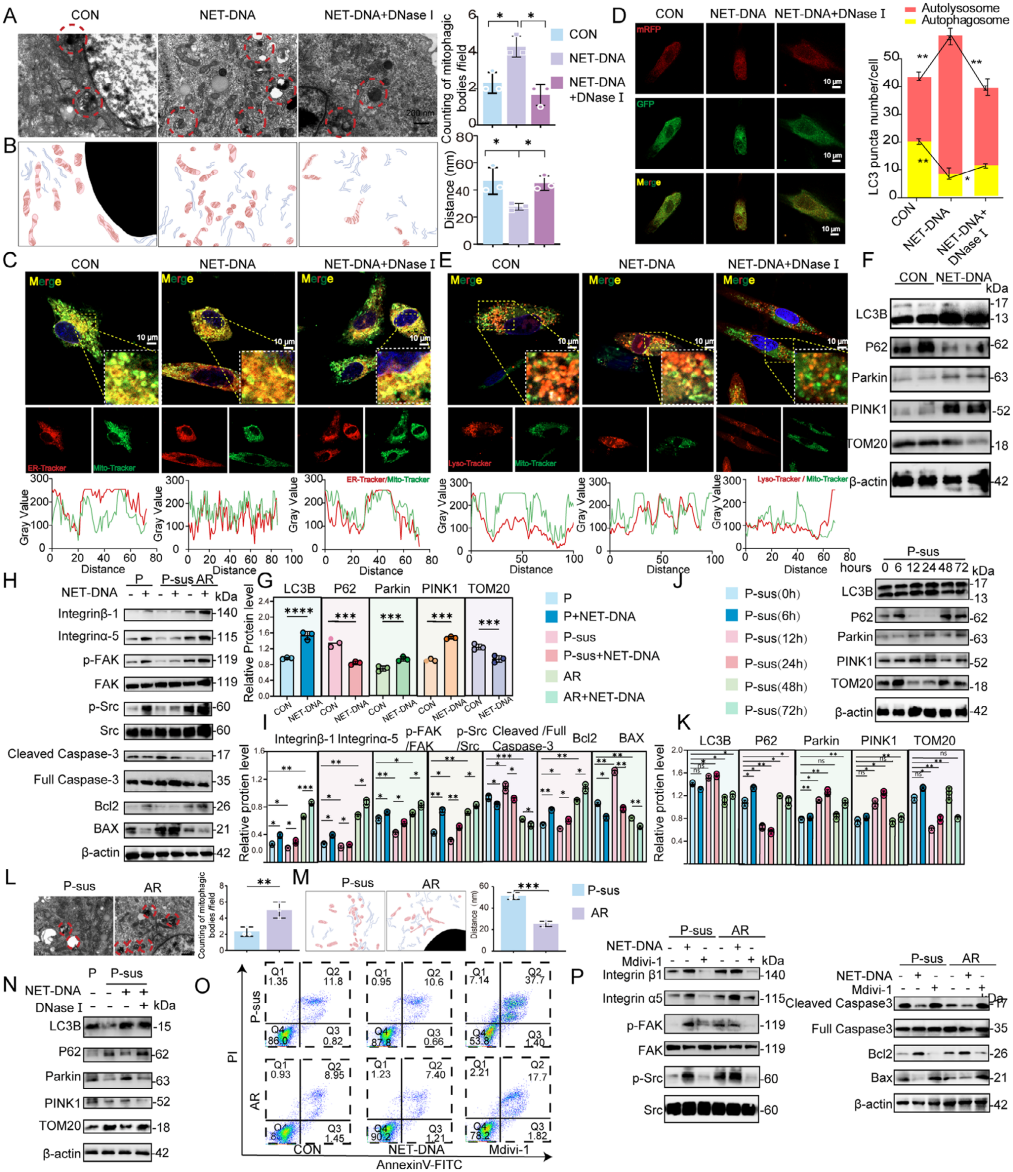
NET‐DNA promotes anoikis resistance in EESCs by sustaining mitophagy. (A,B) Representative TEM images of cells in the CON, NET‐DNA, and NET‐DNA + DNase I groups (A) and redrawn schematic images of mitochondria and endoplasmic reticulum based on the TEM images (B), which were used to quantify the distance between mitochondria and endoplasmic reticulum. Quantification of the number of mitophagic bodies per field and the mitochondria–endoplasmic reticulum distance is shown on the right (n = 3 per group; one‐way ANOVA). Red dashed circles indicate representative mitophagic structures. Scale bar = 200 nm. (C) Representative confocal microscopy images using ER‐Tracker Red and MitoTracker Green, showing that NET‐DNA treatment increased the colocalization of endoplasmic reticulum and mitochondria, whereas DNase I treatment reduced the colocalization of endoplasmic reticulum and mitochondria. Scale bar = 10 µm. (D) Representative images of autophagic flux detected by mCherry‐EGFP‐LC3B and quantification of LC3 puncta, showing that NET‐DNA treatment increased autolysosome formation, whereas DNase I treatment reduced autolysosome formation (n = 3 per group; one‐way ANOVA). Scale bar = 10 µm. (E) Representative images of LysoTracker Red and MitoTracker Green staining and line‐scan fluorescence intensity analysis, showing that NET‐DNA treatment increased the colocalization of mitochondria and lysosomes, whereas DNase I treatment reduced the colocalization of mitochondria and lysosomes. Scale bar = 10 µm. (F,G) Western blot analysis of the effects of NET‐DNA on mitophagy‐related protein expression (F) and the corresponding quantification (G) (n = 3 per group; unpaired Student's *t*‐test). (H,I) Western blot analysis of the effects of different cellular states and NET‐DNA treatment on the expression of integrin signaling‐related, apoptosis‐related, and mitophagy‐related proteins (n = 3 per group; two‐way ANOVA followed by Bonferroni test). (J,K) Western blot analysis of the dynamic changes in mitophagy‐related proteins during the transition from the attached state to the suspended state (J) and the corresponding quantification (K) (n = 3 per group; one‐way ANOVA followed by Dunnett's multiple comparisons test). (L,M) TEM images of P‐sus and AR cells (L) and redrawn schematic images of mitochondria and endoplasmic reticulum based on the TEM images (M). Quantification of the number of mitophagic bodies per field and the mitochondria–endoplasmic reticulum distance is shown on the right (n = 3 per group; unpaired Student's *t*‐test). Red dashed circles indicate representative mitophagic structures. Scale bar = 200 nm. (N) WB analysis confirming that NET‐DNA restores mitophagy‐related protein expression during anoikis, while DNase I reverses this effect (one‐way ANOVA followed by Bonferroni's multiple comparisons test). (O) Annexin V‐FITC/PI flow cytometry analysis showed that NET‐DNA reduced apoptosis in both P‐sus and AR EESCs, whereas the mitochondrial fission inhibitor Mdivi‐1 (10 µm) increased apoptosis. (two‐way ANOVA followed by Bonferroni test). (P) WB validation of autophagy and apoptosis‐related proteins corresponding to functional assays presented in P‐sus and AR cells (two‐way ANOVA followed by Bonferroni test). Data are expressed as mean ± SD. *P < 0.05; **P < 0.01; ***P < 0.001.

Transcriptomic analysis showed that NETs stimulation increased anoikis resistance in EESCs. However, TEM analysis of mouse lesions revealed a marked rise in autophagosome formation after LPS treatment, indicating increased autophagic activity in ectopic stromal cells at an early stage. Based on these findings, we next examined whether mitophagy was altered in NET‐DNA‐treated EESCs. In primary EESCs treated with NET‐DNA, TEM analysis (Figure 3A) revealed that NET‐DNA significantly increased autophagosome formation and reduced MAM distances compared to controls; notably, DNase I pretreatment reduced autophagosome formation and restored MAM distance toward control levels (Figure 3B). Consistently, ER‐Tracker and MitoTracker co‐staining and the subsequent colocalization line‐scan analysis (Figure 3C) showed that NET‐DNA treatment increased ER‐mitochondria colocalization, whereas DNase I treatment reduced ER‐mitochondria colocalization. To further monitor autophagic flux, cells expressing mCherry–EGFP–LC3 were analyzed (Figure 3D). NET‐DNA markedly increased the number of mCherry‐positive but GFP‐negative LC3 puncta, indicating enhanced autophagosome–lysosome fusion and accelerated autophagic flux. DNase I pretreatment abolished the NET‐DNA‐induced increase in mCherry‐positive/GFP‐negative LC3 puncta. NET‐DNA also increased MitoTracker–LysoTracker colocalization (Figure 3E), whereas DNase I markedly reduced MitoTracker–LysoTracker overlap, further confirming NET‐DNA–mediated mitophagy in EESCs. Western blot analysis (Figure 3F) showed that NET‐DNA treatment led to increased expression of mitophagy‐related proteins PINK1 and Parkin, decreased expression of TOM20 and P62, and reduced LC3B levels, supporting the activation of mitophagy (Figure 3G). These results indicate that NET‐DNA promotes mitophagy and is associated with reduced anoikis in EESCs. To explore mitophagy's role in anoikis resistance, anoikis (P‐sus) and anoikis‐resistant (AR) models were established. Western blot analysis (Figure 3H) and quantification (Figure 3I) showed that NET‐DNA significantly reduced apoptotic markers in both P‐sus and AR cells. Time‐course analysis (Figure 3J) and quantification (Figure 3K) showed that autophagy peaked at 24 h post‐detachment in P‐sus cells, gradually declining thereafter. TEM further demonstrated that AR cells had more mitophagosomes and more intact MAM structures than P‐sus cells, consistent with increased mitophagy and closer ER‐mitochondria contacts during anoikis resistance (Figure 3L,M). Western blot analysis (Figure 3N) confirmed that NET‐DNA restored autophagy‐related protein expression during anoikis, and this restoration was abolished by DNase I treatment. To assess the role of mitophagy in anoikis regulation, apoptosis was measured in P‐sus and AR EESCs. Annexin V/PI staining (Figure 3O) showed that NET‐DNA significantly reduced apoptosis in both P‐sus and AR cells, consistent with an association between increased mitophagy and reduced apoptosis. In contrast, treatment with the mitophagy inhibitor Mdivi‐1 increased apoptosis compared with control cells (Figure S1I). Western blot (Figure 3P; Figure S1J) confirmed corresponding changes in apoptotic and mitophagy‐associated markers. These findings indicate that NET‐DNA supports EESCs survival through protective mitophagy, thereby contributing to anoikis resistance. In addition, Western blot of AR cells under different conditions—including adherent, suspension, suspension plus NET‐DNA, suspension plus Mdivi‐1, and adherent plus NET‐DNA—further confirmed that NET‐DNA restored integrin–FAK–Src signaling and suppressed apoptosis in suspended cells, whereas mitophagy inhibition by Mdivi‐1 increased apoptosis and reduced survival signaling (Figure S1K). Collectively, these results demonstrate that NET‐DNA promotes EESCs survival by increasing mitophagy, which is associated with anoikis resistance during early endometriotic lesion development.

### 3.4 NET‐DNA Upregulates TMEM215, Regulating Mitophagy and MAM Homeostasis to Mediate Anoikis Resistance

**FIGURE 4:**
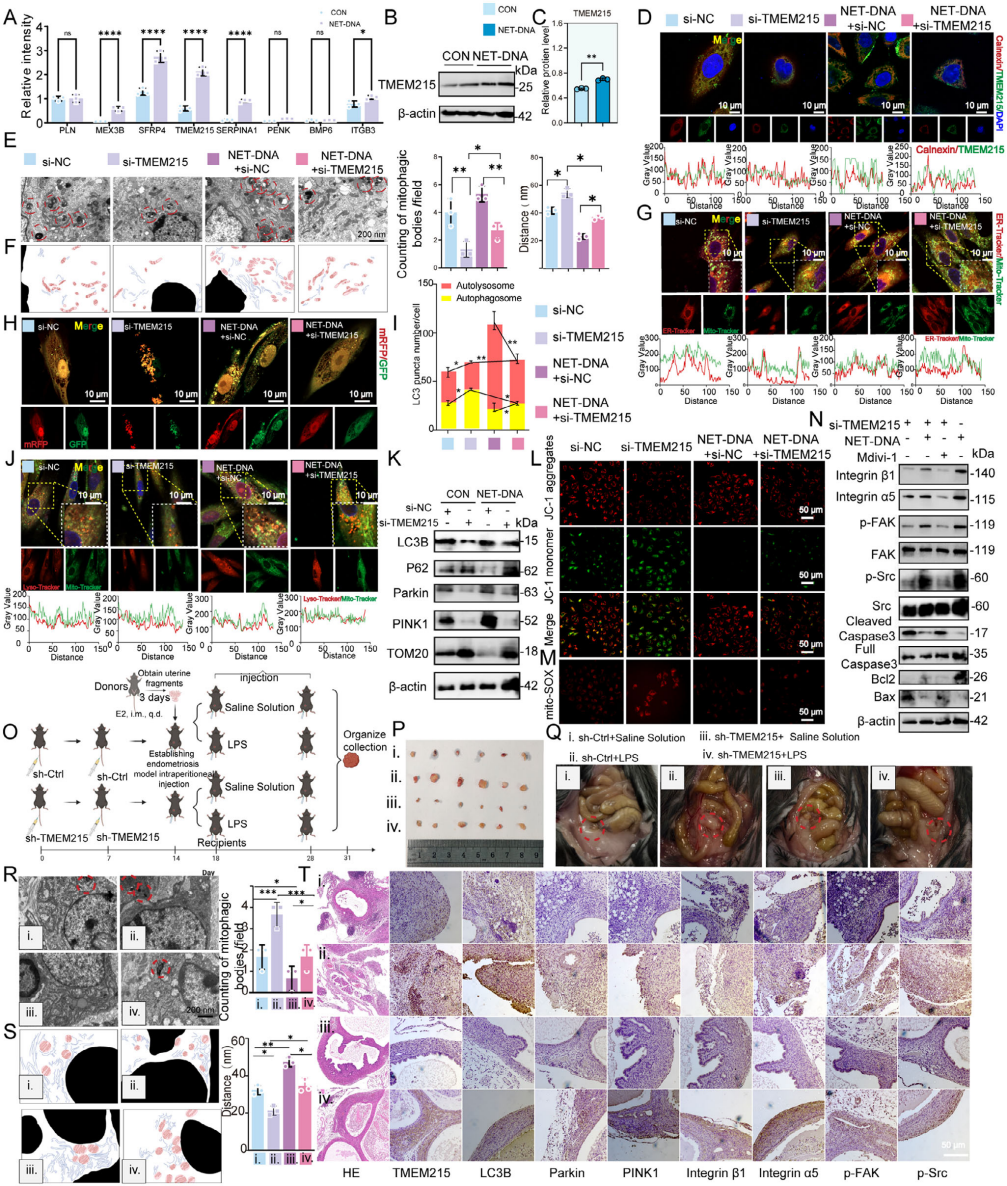
NET‐DNA induces TMEM215 expression to regulate mitophagy and MAM homeostasis, mediating anoikis resistance. (A) qRT‐PCR analysis of candidate genes following NET‐DNA treatment, showing TMEM215 as the most prominently upregulated gene (n = 3; unpaired Student's *t*‐test). (B,C) Western blot analysis of the effect of NET‐DNA on TMEM215 protein expression (B) and the corresponding quantification (C) (n = 3; unpaired Student's *t*‐test). (D) IF colocalization of TMEM215 with calnexin, indicating stable ER localization under basal and NET‐DNA conditions. Scale bar = 10 µm. (E,F) Representative transmission electron microscopy images (E) and redrawn schematic images of mitochondria and endoplasmic reticulum based on the TEM images (F) in si‐NC or si‐TMEM215 cells with or without NET‐DNA treatment, which were used to quantify the distance between mitochondria and endoplasmic reticulum. Quantification of the number of mitophagic bodies per field and the mitochondria–endoplasmic reticulum distance is shown on the right (n = 3; one‐way ANOVA followed by Bonferroni's multiple comparisons test). Red dashed circles indicate representative mitophagic structures. Scale bar = 200 nm. (G) ER‐Tracker and MitoTracker staining demonstrating enhanced ER–mitochondria colocalization induced by NET‐DNA, attenuated by TMEM215 knockdown. Scale bar = 10 µm. (H,I) Representative images of autophagic flux detected by mRFP‐GFP‐LC3B (H) and quantification of LC3 puncta (I), indicating elevated autophagic flux upon NET‐DNA treatment, reduced by TMEM215 knockdown (one‐way ANOVA followed by Bonferroni's multiple comparisons test). Scale bar = 10 µm. (J) MitoTracker and LysoTracker staining illustrating increased mitochondria–lysosome interactions after NET‐DNA exposure, weakened by TMEM215 knockdown. Scale bar = 10 µm. (K) Western blot analysis of changes in mitophagy‐related protein expression in si‐NC or si‐TMEM215 cells with or without NET‐DNA treatment (n = 3; one‐way ANOVA followed by Bonferroni's multiple comparisons test). (L,M) JC‐1 staining (L) and MitoSOX staining (M) were used to detect mitochondrial membrane potential and mitochondrial ROS levels under the indicated conditions, respectively. (N) WB analysis of the effects of TMEM215 knockdown and Mdivi‐1 treatment on NET‐DNA‐related integrin signaling and apoptosis‐related protein expression (n = 3; one‐way ANOVA followed by Bonferroni's multiple comparisons test). (O) Schematic diagram illustrating AAV9‐shTMEM215 delivery and EMs induction protocol. The schematic was created with BioRender.com. (P,Q) Representative gross images of ectopic lesions (P) and representative images showing lesion establishment in the abdominal cavity (Q) in the sh‐Ctrl + Saline Solution, sh‐Ctrl + LPS, sh‐TMEM215 + Saline Solution, and sh‐TMEM215 + LPS groups (n = 6 per group). (R,S) Representative transmission electron microscopy images of lesion tissues from the above four groups (R) and redrawn schematic images of mitochondria and endoplasmic reticulum based on the TEM images (S), with quantification of the number of mitophagic bodies per field and the mitochondria–endoplasmic reticulum distance shown on the right (n = 3; one‐way ANOVA followed by Bonferroni's multiple comparisons test). Red dashed circles indicate representative mitophagic structures. Scale bar = 200 nm. (T) H&E and IHC staining demonstrating disrupted glandular structure and decreased expression of LC3, PINK1, Parkin, and phosphorylated integrin β1/α5, FAK, and SRC following TMEM215 knockdown (n = 6 per group). Scale bar = 50 µm. Data are expressed as mean ± SD. *P < 0.05; **P < 0.01; ***P < 0.001; ****P < 0.0001.

To investigate NET‐DNA's regulation of mitochondrial homeostasis in EESCs, candidate gene expression was analyzed. qRT‐PCR identified TMEM215 as the most significantly upregulated gene after NET‐DNA stimulation (Figure 4A), which was confirmed by Western blot (Figure 4B,C). Previous studies have shown that TMEM215 localizes to the ER. In our system, TMEM215 expression was effectively silenced by TMEM215 siRNA and robustly increased by TMEM215 overexpression constructs, confirming efficient knockdown and overexpression in EESCs (Figure S2A). Under basal and NET‐DNA‐stimulated conditions, TMEM215 consistently colocalized with the ER marker calnexin, confirming its ER residence (Figure 4D). Given TMEM215's stable ER localization, we next examined whether TMEM215 affects ER–mitochondria contacts and mitophagy in EESCs. TEM analysis (Figure 4E) showed that NET‐DNA markedly increased autophagosome formation and reduced MAM distances (Figure 4F) compared with controls. TMEM215 knockdown decreased the number of autophagosomes, increased MAM distances, and attenuated the effects of NET‐DNA. In agreement with the TEM findings, ER‐Tracker and MitoTracker double staining (Figure 4G) consistently demonstrated that NET‐DNA enhanced ER–mitochondria colocalization, partially reversed by TMEM215 knockdown. To further evaluate mitophagy activation, mitophagic flux tracing with mCherry‐EGFP‐LC3B (Figure 4H,I) indicated that NET‐DNA elevated mCherry‐positive/GFP‐negative LC3 puncta and increased their colocalization with mitochondria and lysosomes, while TMEM215 knockdown attenuated these effects. Similarly, NET‐DNA increased MitoTracker–LysoTracker colocalization (Figure 4J), while TMEM215 knockdown reduced this overlap, further confirming NET‐DNA–mediated mitophagy in EESCs. Western blot analysis (Figure 4K) confirmed that NET‐DNA upregulated PINK1, Parkin, and LC3B, and downregulated TOM20 and P62, which was reversed by TMEM215 knockdown, supporting the activation of mitophagy. Western blot quantification in Figure S2G further confirmed the mitophagy‐related protein expression in the context of TMEM215 knockdown and NET‐DNA treatment. Functionally, JC‐1 (Figure 4L) staining demonstrated that NET‐DNA increased the JC‐1 red/green fluorescence ratio, whereas TMEM215 knockdown significantly reduced this ratio, indicating mitochondrial depolarization. MitoSOX staining (Figure 4M) confirmed that NET‐DNA treatment lowered mitochondrial ROS levels, and this reduction was abolished by TMEM215 knockdown, indicating that TMEM215 is required for the NET‐DNA‐associated improvement in mitochondrial status. To further assess the role of TMEM215 in survival signaling, EESCs were treated with NET‐DNA, TMEM215 siRNA, or the mitophagy inhibitor Mdivi‐1. NET‐DNA enhanced integrin–FAK signaling and reduced apoptotic markers, indicating a prosurvival effect. TMEM215 knockdown markedly attenuated these NET‐DNA–induced signals. In TMEM215‐silenced cells, Mdivi‐1 further suppressed residual NET‐DNA–related survival signaling, supporting an association between mitophagy and TMEM215‐dependent regulation of anoikis resistance (Figure 4N; Figure S2H). To validate TMEM215 function in vivo, a mouse model was established as depicted in Figure 4O (created in BioRender.com). Mice received tail‐vein injections of pAAV9‐TMEM215‐shRNA or sh‐Ctrl before EMs induction. After lesion formation, mice were divided into four groups: sh‐Ctrl + saline, sh‐Ctrl + LPS, AAV9‐TMEM215‐shRNA + saline, and AAV9‐TMEM215‐shRNA + LPS. LPS significantly promoted lesion growth, whereas TMEM215 knockdown suppressed this growth and partially reversed LPS‐induced lesion formation (Figure 4P,Q; Figure S3A). TEM analysis (Figure 4R,S) showed abundant autophagosomes and closely arranged MAMs in LPS‐treated lesions, whereas TMEM215 knockdown reduced autophagosome numbers and increased ER–mitochondria spacing. HE staining revealed reduced glandular structures and disorganized stromal architecture in the sh‐TMEM215 group (Figure 4T). IHC confirmed that TMEM215 deficiency lowered LC3, PINK1, and Parkin expression and decreased phosphorylation of integrin β1, integrin α5, FAK, and SRC (Figure 4T).

Together, these data support that TMEM215 is required for NET‐DNA‐induced changes in ER–mitochondria contacts, mitophagy‐related signaling, and integrin–FAK–SRC activation, and is associated with reduced anoikis and increased lesion formation.

### 3.5 NET‐DNA Directly Binds to Membrane ANXA2 and Induces Its Translocation to the Cytoplasm

**FIGURE 5:**
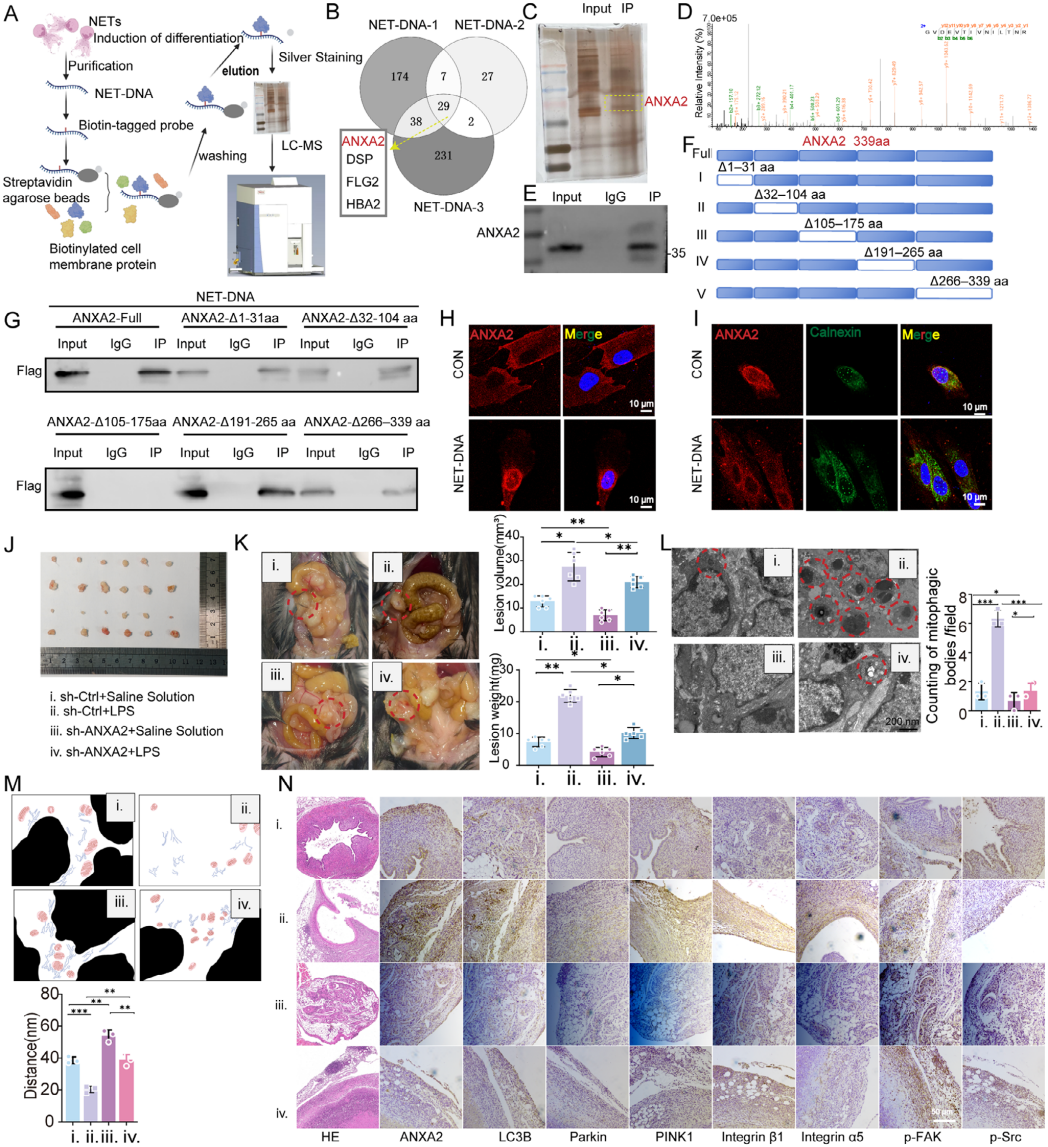
NET‐DNA directly binds to membrane ANXA2 and induces its translocation to the cytoplasm. (A) Workflow of a DNA pull‐down assay using biotin‐labeled NET‐DNA to isolate NET‐DNA–binding plasma membrane proteins from EESCs. The schematic was created with BioRender.com. (B) Venn diagram illustrating overlapping NET‐DNA–interacting proteins identified by three independent LC–MS/MS pull‐down experiments. (C) Silver staining of pull‐down eluates demonstrating protein bands enriched by NET‐DNA. (D) Representative LC–MS/MS spectrum identifying ANXA2 as a NET‐DNA–binding protein. (E) DNA pull‐down validation confirming the specific interaction between NET‐DNA and ANXA2. (F) Schematic representation of full‐length ANXA2 and its deletion mutants (Δ1–31, Δ32–104, Δ105–175, Δ191–265, Δ266–339 aa). (G) DNA pull‐down assays indicating that the 105–175 aa region of ANXA2 is required for NET‐DNA binding. (H) IF images demonstrating decreased membrane‐associated ANXA2 and increased cytoplasmic localization following NET‐DNA treatment; Calnexin marks the ER. Scale bar = 10 µm. (I) Colocalization analysis showing enhanced ANXA2–calnexin overlap in NET‐DNA–treated cells. Scale bar = 10 µm. (J,K) Representative images of ectopic lesions (J) and representative images showing lesion establishment in the abdominal cavity together with quantification of lesion volume and weight (K) in the sh‐Ctrl + Saline Solution, sh‐Ctrl + LPS, sh‐ANXA2 + Saline Solution, and sh‐ANXA2 + LPS groups (n = 6; one‐way ANOVA followed by Bonferroni's multiple comparisons test). (L,M) Representative transmission electron microscopy images of lesion tissues from the above four groups (L) and redrawn schematic images of mitochondria and endoplasmic reticulum based on the TEM images (M), with quantification of the number of mitophagic bodies per field and the mitochondria–endoplasmic reticulum distance shown on the right (n = 3; one‐way ANOVA followed by Bonferroni's multiple comparisons test). Red dashed circles indicate representative mitophagic structures. Scale bar = 200 nm. (N) H&E and IHC staining indicating reduced expression of ANXA2, mitophagy‐related proteins, and integrin signaling‐related proteins in ANXA2‐silenced lesions (n = 6). Scale bar = 50 µm. Data are expressed as mean ± SD. *P < 0.05; **P < 0.01; ***P < 0.001.

To identify potential membrane‐associated proteins interacting with NET‐DNA in EESCs, biotin‐labeled NET‐DNA was incubated with isolated plasma membrane proteins from EESCs. A DNA pull‐down assay was then performed according to the workflow shown in Figure 5A (created in BioRender.com). Silver staining and mass spectrometry identified multiple candidate proteins binding to NET‐DNA. The intersection of three independent experiments determined the final set of interacting proteins (Figure 5B–D). Among these candidates, ANXA2 was selected for further analysis because of its reported association with autophagy‐related processes. The DNA pull‐down assay further supported an interaction between NET‐DNA and ANXA2, while no visible band appeared in the negative control (Figure 5E). Based on the full‐length ANXA2 sequence (339 amino acids), several deletion mutants were generated: Δ1–31 aa (mutant I), Δ32–104 aa (mutant II), Δ105–175 aa (mutant III), Δ191–265 aa (mutant IV), and Δ266–339 aa (mutant V) (Figure 5F). DNA pull‐down analysis showed that the Δ105–175 aa region is essential for NET‐DNA binding (Figure 5G). Next, we assessed whether NET‐DNA stimulation affects ANXA2 expression and localization. WB analysis showed that NET‐DNA did not significantly alter total ANXA2 protein levels (Figure S2B). However, membrane–cytoplasmic fractionation revealed a notable decrease of ANXA2 at the plasma membrane and a corresponding increase in the cytoplasm (Figure S2C). These results support a redistribution of ANXA2 from the membrane to the cytoplasm after NET‐DNA treatment. IF further confirmed these observations: NET‐DNA–treated cells exhibited reduced ANXA2 signals at the plasma membrane and increased colocalization with the ER marker calnexin, indicating ANXA2 relocation toward ER regions (Figure 5H,I). To evaluate ANXA2's physiological role in vivo, an EMs mouse model was established as described in Figure 4O. Mice received tail‐vein injections of pAAV9‐ANXA2‐shRNA or control vector (pAAV9‐Ctrl) 14 days before disease induction. After ectopic lesion formation, animals were divided randomly into four groups: pAAV9‐Ctrl + saline, pAAV9‐Ctrl + LPS, pAAV9‐ANXA2‐shRNA + saline, and pAAV9‐ANXA2‐shRNA + LPS. LPS treatment markedly promoted lesion growth, while ANXA2 silencing significantly reduced lesion size and partially reversed the pro‐survival effects of LPS (Figure 5J,K). TEM analysis (Figure 5L) showed abundant autophagosomes and tightly arranged MAMs in lesions treated with LPS. In contrast, ANXA2 depletion led to fewer autophagosomes, swollen mitochondria, and increased MAM distances (Figure 5M). HE staining further revealed decreased glandular structures and looser stromal organization in the sh‐ANXA2 group (Figure 5N). IHC analysis demonstrated that ANXA2 knockdown significantly reduced mitophagy‐related protein expression (LC3, PINK1, and Parkin). Similar to TMEM215, the expression and phosphorylation of integrin β1, integrin α5, FAK, and SRC strongly increased in the LPS group but significantly decreased upon ANXA2 silencing (Figure 5N). Overall, these findings support that NET‐DNA binds ANXA2 and is associated with redistribution of ANXA2 from the membrane to the cytoplasm, accompanied by increased localization in ER regions. In parallel, ANXA2 knockdown attenuated LPS‐associated increases in lesion growth, mitophagy‐related markers, and integrin signaling, supporting an association of ANXA2 with ER–mitochondria contacts, mitophagy‐related changes, and anoikis resistance in EESCs.

### 3.6 NET‐DNA Enhances TMEM215 and ANXA2 Interaction in a Ca^2+^‐Dependent Manner

**FIGURE 6:**
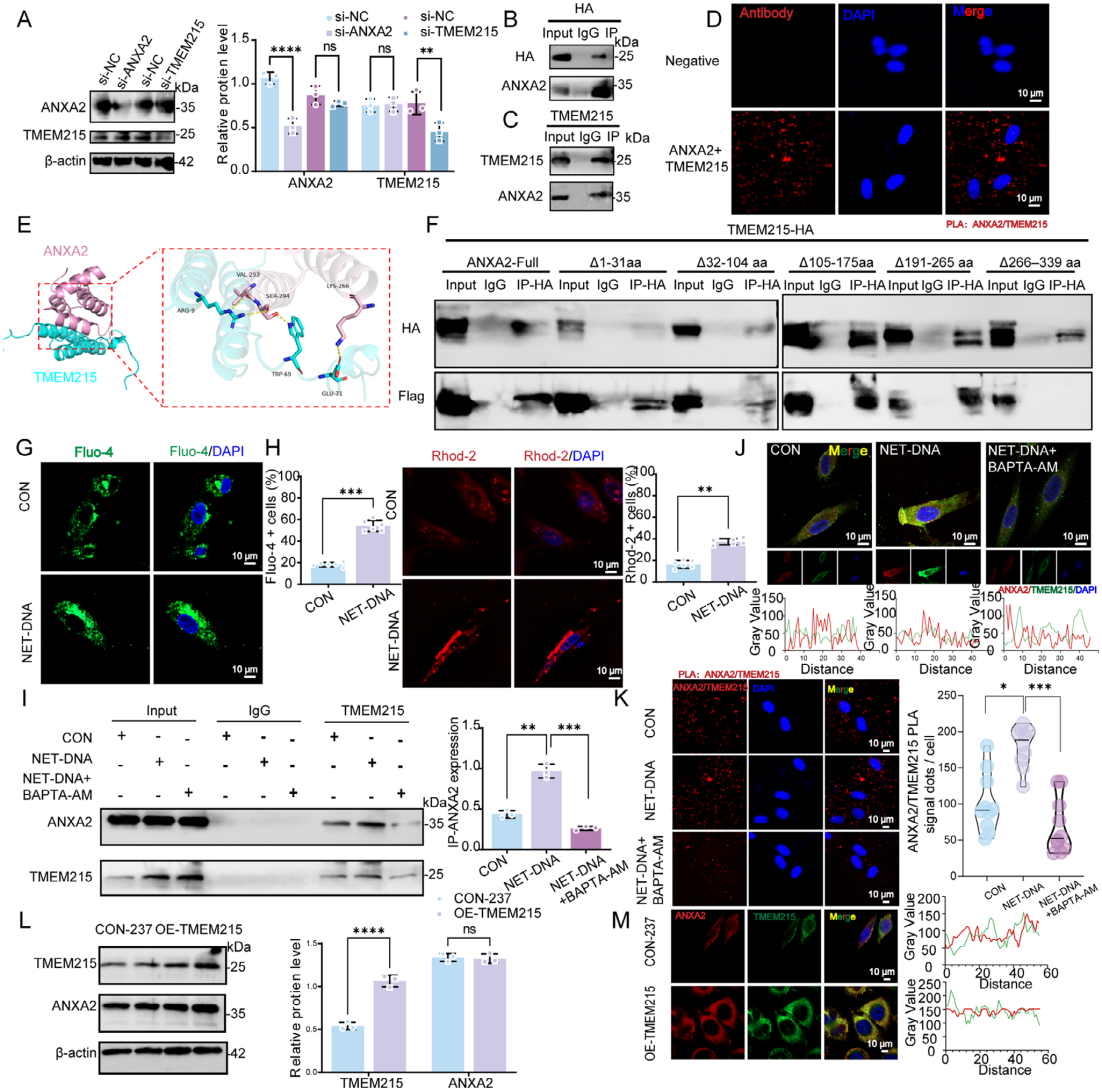
NET‐DNA enhances the TMEM215–ANXA2 interaction in a Ca^2+^‐dependent manner. (A) WB demonstrating that silencing ANXA2 or TMEM215 does not affect the protein expression of the other (n = 3; unpaired Student's *t*‐test). (B,C) Co‐IP assays in EESCs transfected with HA‐TMEM215 (B) or under endogenous conditions (C), confirming the TMEM215–ANXA2 interaction. (D) PLA assay revealing strong red signals indicative of robust TMEM215–ANXA2 interactions. Scale bar = 10 µm. (E) Molecular docking model depicting a stable ANXA2–TMEM215 heteromeric complex. (F) Pull‐down assays using ANXA2 deletion mutants showing TMEM215 preferentially binds the 266–339 aa region of ANXA2. (G,H) Fluo‐4 AM (G) and Rhod‐2 AM (H) staining indicating increased cytosolic and mitochondrial Ca^2+^ levels in cells stimulated with NET‐DNA (n = 3; unpaired Student's *t*‐test). Scale bar = 10 µm. (I) Co‐IP assays demonstrating that NET‐DNA enhances TMEM215–ANXA2 interaction, whereas the Ca^2+^ chelator BAPTA‐AM significantly reduces this interaction (n = 3; one‐way ANOVA followed by Bonferroni post‐hoc test). (J,K) IF (J) and PLA assays (K) further confirming attenuation of NET‐DNA–induced TMEM215–ANXA2 binding by BAPTA‐AM (K, n = 10; one‐way ANOVA followed by Bonferroni post‐hoc test). Scale bar = 10 µm. (L) WB analysis showing TMEM215 overexpression does not alter total ANXA2 protein levels (n = 3; unpaired Student's *t*‐test). (M) IF staining showing increased cytoplasmic accumulation of ANXA2 upon TMEM215 overexpression. Scale bar = 10 µm. Data are expressed as mean ± SD. ns, not significant; *P < 0.05; **P < 0.01; ***P < 0.001.

To clarify how TMEM215 and ANXA2 contribute to NET‐DNA‐mediated signaling, we first examined whether a hierarchical regulatory relationship exists between them. ANXA2 knockdown did not affect TMEM215 expression; likewise, silencing TMEM215 did not alter ANXA2 protein levels (Figure 6A). Because ANXA2 translocates from the plasma membrane to the cytoplasm upon NET‐DNA stimulation and colocalizes with the ER, we next examined whether TMEM215 and ANXA2 interact in EESCs. Co‐immunoprecipitation (Co‐IP) in EESCs transiently transfected with HA‐tagged TMEM215 showed strong enrichment of ANXA2, supporting their interaction (Figure 6B). This association was further validated under endogenous conditions (Figure 6C). Proximity Ligation Assay (PLA) demonstrated robust red fluorescent signals in EESCs, further supporting close association between TMEM215 and ANXA2 (Figure 6D). Molecular docking analysis predicted a potential interaction interface between ANXA2 (pink) and TMEM215 (cyan). Key residues of ANXA2 (VAL‐293, SER‐294, and LYS‐266) were predicted to form hydrogen bonds with ARG‐9, TRP‐69, and GLU‐71 of TMEM215, respectively (Figure 6E). Pull‐down assays using ANXA2 truncation mutants confirmed that TMEM215 mainly interacts with the 266–339 aa region of ANXA2 (Figure 6F). As this region is adjacent to the Ca^2+^‐binding domain, we next examined whether their interaction was associated with Ca^2+^ signaling. Calcium dynamics were assessed using Fluo‐4 AM and Rhod‐2 AM. NET‐DNA stimulation significantly increased cytosolic and mitochondrial Ca^2+^‐positive cells (Figure 6G,H). Subsequent Co‐IP experiments revealed that NET‐DNA enhanced the TMEM215–ANXA2 interaction, with increased ANXA2 co‐precipitated by TMEM215. Treatment with the calcium chelator BAPTA‐AM weakened this interaction (Figure 6I). IF (Figure 6J) and PLA (Figure 6K) analyses confirmed that BAPTA‐AM reduced TMEM215–ANXA2 binding. NET‐DNA treatment was also associated with increased TMEM215 expression, redistribution of ANXA2 from the membrane to the cytoplasm/ER‐associated regions, enhanced ER–mitochondria contacts, and increased mitochondrial Ca^2+^ signals. Previous studies reported that in chronic pathological cardiac conditions, TMEM215 downregulation coincides with cytosolic and mitochondrial Ca^2+^ overload. These findings suggest that altered Ca^2+^ status may be linked to changes in TMEM215‐associated ER–mitochondria regulation. WB analysis confirmed that HA‐TMEM215 overexpression did not alter total ANXA2 levels (Figure 6L), whereas IF showed enhanced ANXA2 cytoplasmic localization (Figure 6M). Collectively, these findings support that NET‐DNA induces cytosolic Ca^2+^ elevation and promotes TMEM215–ANXA2 interaction in a calcium‐associated manner. This interaction is accompanied by redistribution of ANXA2 from the membrane to the cytoplasm, enhanced ER–mitochondria contacts, and increased mitochondrial Ca^2+^ uptake, and may contribute to EESCs survival and anoikis resistance under detachment conditions.

### 3.7 TMEM215–ANXA2 Axis Promotes Anoikis Resistance by Maintaining MAM Integrity and Regulating Mitophagy

**FIGURE 7:**
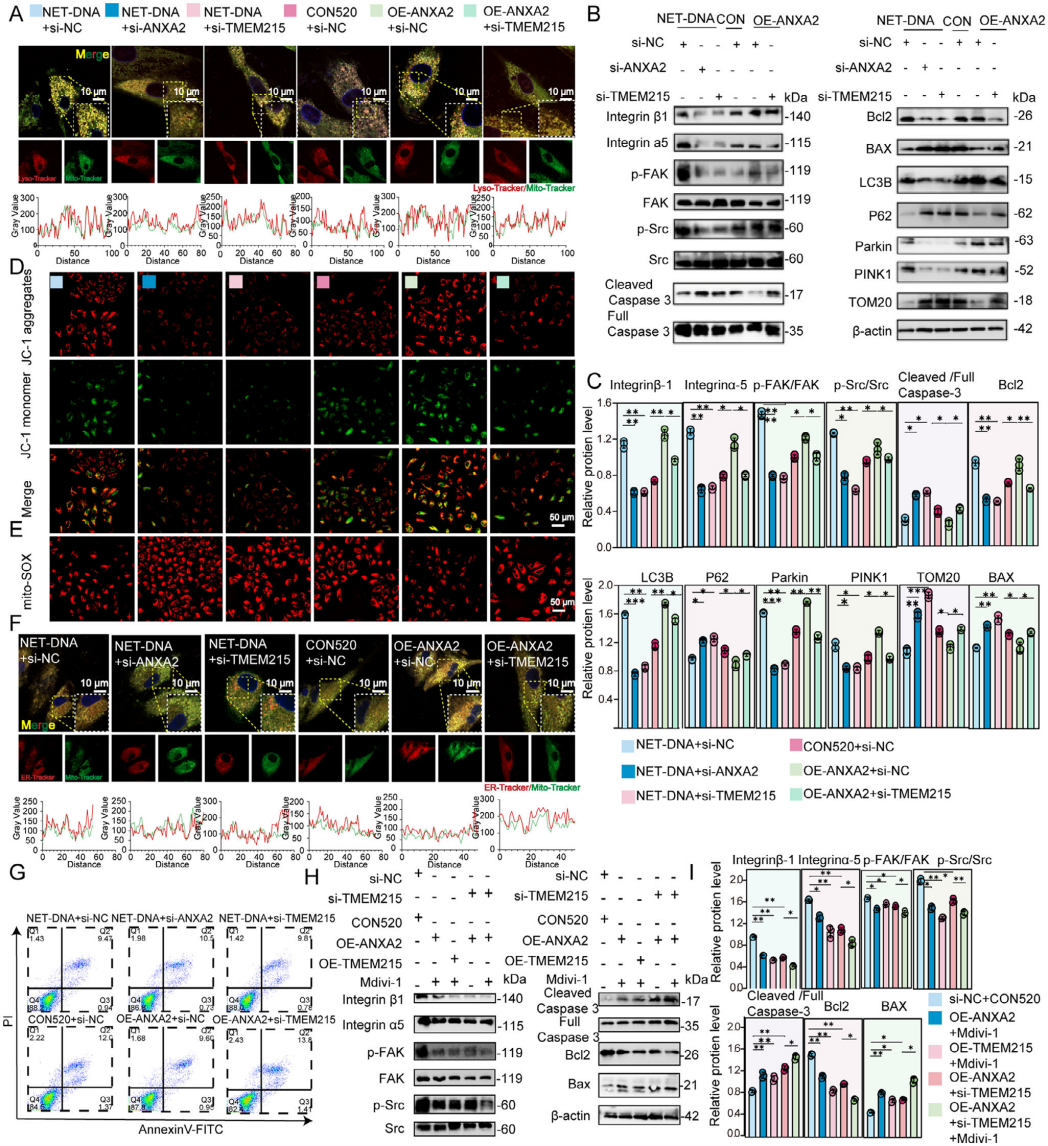
TMEM215–ANXA2 axis promotes anoikis resistance by maintaining MAM integrity and regulating mitophagy. (A) Representative images of LysoTracker Red and MitoTracker Green co‐staining and line‐scan fluorescence intensity analysis, showing changes in mitochondria‐lysosome colocalization under NET‐DNA stimulation and ANXA2 or TMEM215 intervention. Scale bar = 10 µm. (B,C) Western blot analysis of the expression changes of integrin signaling pathways, apoptosis‐related proteins, and mitophagy‐related proteins under NET‐DNA stimulation and ANXA2 or TMEM215 intervention (B), along with corresponding quantification analysis (C) (n = 3; one‐way ANOVA followed by Bonferroni post‐hoc test). (D, E) JC‐1 staining (D) and MitoSOX staining (E) to detect changes in mitochondrial membrane potential and mitochondrial ROS levels under NET‐DNA stimulation and ANXA2 or TMEM215 intervention. Scale bar = 50 µm. (F) ER‐Tracker and MitoTracker co‐staining demonstrating enhanced ER–mitochondria contacts after NET‐DNA stimulation, disrupted upon ANXA2 or TMEM215 knockdown. Scale bar = 10 µm. (G) Flow cytometry showing that NET‐DNA suppresses detachment‐induced apoptosis, whereas apoptosis rates are restored after ANXA2 or TMEM215 knockdown (one‐way ANOVA followed by Bonferroni post‐hoc test). (H,I) Western blot analysis of the effects of Mdivi‐1 intervention on the expression of integrin signaling pathway and apoptosis‐related proteins induced by ANXA2 and TMEM215 (H), along with corresponding quantification analysis (I) (n = 3; one‐way ANOVA followed by Bonferroni post‐hoc test). Data are expressed as mean ± SD. *P < 0.05; **P < 0.01.

To clarify how the TMEM215–ANXA2 complex regulates mitochondrial homeostasis and anti‐apoptotic capacity upon NET‐DNA stimulation, EESCs were treated with NET‐DNA, subjected to ANXA2 or TMEM215 knockdown, or underwent ANXA2 overexpression. The efficiency of ANXA2 silencing and overexpression was confirmed prior to functional assays (Figure S2D). Mitophagy‐related changes were first assessed. Co‐staining with LysoTracker Red and MitoTracker Green showed that NET‐DNA strongly increased mitochondria–lysosome colocalization and autophagosome formation, while ANXA2 or TMEM215 depletion completely reversed these effects (Figure 7A). Western blot analysis (Figure 7B) and its quantification (Figure 7C) further demonstrated that NET‐DNA elevated LC3‐II, PINK1, and Parkin levels, and decreased TOM20 and P62 expression. ANXA2 or TMEM215 knockdown reversed these changes. Next, mitochondrial function was evaluated. JC‐1 staining (Figure 7D) indicated that NET‐DNA increased the JC‐1 red/green fluorescence ratio, whereas ANXA2 or TMEM215 silencing markedly reduced ΔΨm. MitoSOX (Figure 7E) analysis of mitochondrial oxidative stress revealed that NET‐DNA decreased mitochondrial ROS production; this protective effect was weakened by TMEM215 or ANXA2 depletion. ER‐Tracker and MitoTracker co‐staining further demonstrated that NET‐DNA promoted close ER–mitochondria contacts, whereas ANXA2 or TMEM215 knockdown increased the ER–mitochondria distance (Figure 7F). Regarding cell survival, flow cytometry showed that NET‐DNA significantly inhibited apoptosis induced by detachment, but ANXA2 or TMEM215 knockdown restored apoptotic rates (Figure 7G; Figure S3B). WB results confirmed these observations, as NET‐DNA reduced cleaved Caspase‐3 and increased Bcl‐2 expression. ANXA2 or TMEM215 depletion completely reversed these changes. Additionally, treatment with Mdivi‐1 abolished the protective effects of ANXA2 or TMEM215 overexpression, indicated by increased cleaved Caspase‐3 and decreased Bcl‐2 levels (Figure 7H,I). These results support that the TMEM215–ANXA2 axis is associated with increased ER–mitochondria contacts and mitophagy‐related activity. This axis is also associated with preservation of mitochondrial membrane potential and reduced detachment‐induced apoptosis. These effects were attenuated by mitophagy inhibition or disruption of TMEM215/ANXA2 expression, supporting an association of the NET‐DNA–ANXA2–TMEM215 axis with EESCs survival and ectopic lesion development.

### 3.8 TMEM215, ANXA2, and BiP Form a Ternary Complex

**FIGURE 8:**
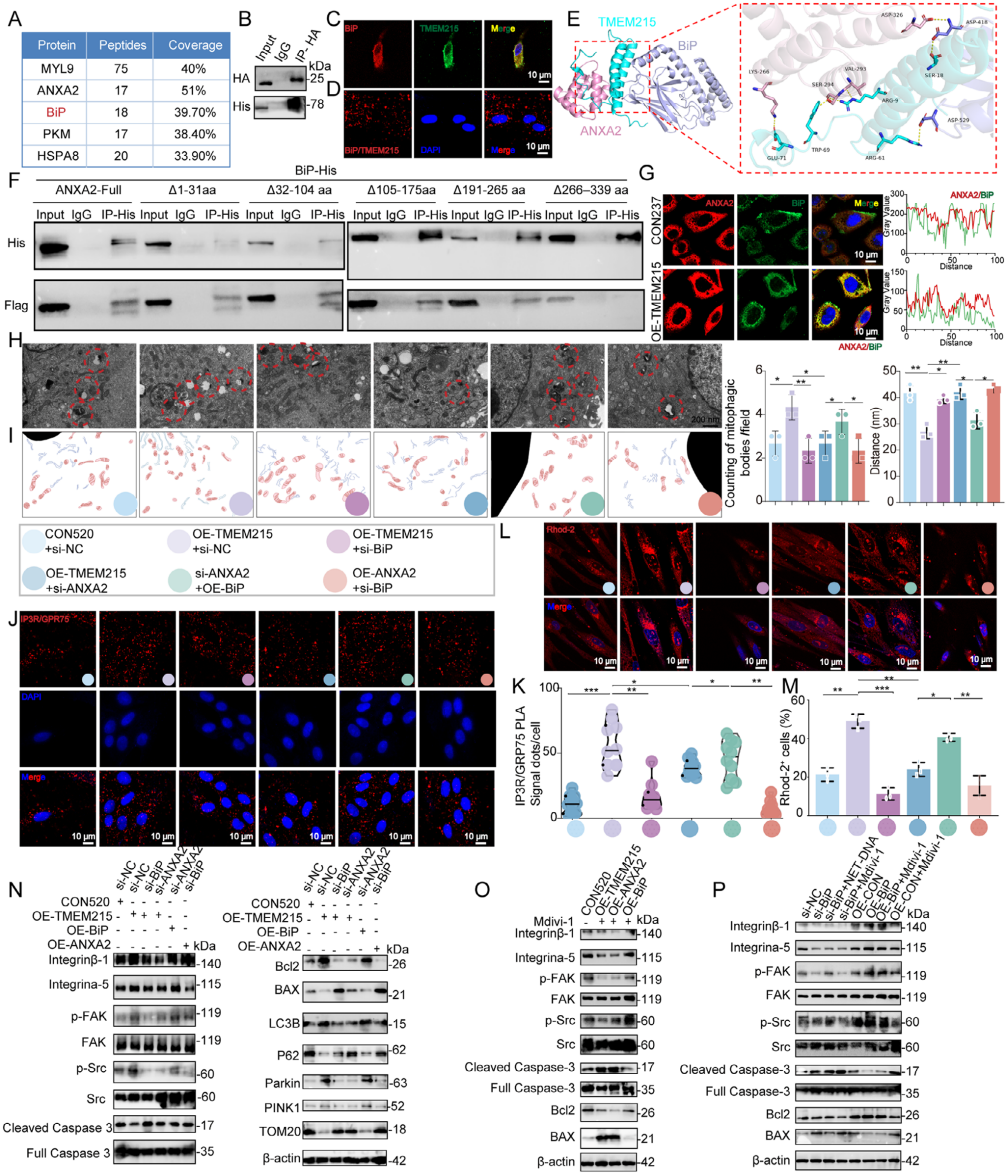
TMEM215, ANXA2, and BiP form a ternary complex. (A) IP‐MS analysis of TMEM215 immunoprecipitates identifying the molecular chaperone BiP (GRP78) as a potential interacting partner. (B) Co‐IP assay with His‐tagged BiP confirming the interaction between TMEM215 and BiP. (C,D) IF colocalization (C) and PLA assays (D) demonstrating strong cytoplasmic proximity between TMEM215 and BiP. Scale bar = 10 µm. (E) Molecular docking illustrating stable binding interfaces supporting the formation of a TMEM215–ANXA2–BiP ternary complex. (F) Pull‐down assays using ANXA2 truncation mutants identifying the 266–339 aa region as essential for BiP binding. (G) IF showing that TMEM215 overexpression markedly enhances ANXA2–BiP colocalization. Scale bar = 10 µm. (H) TEM images showing that TMEM215 overexpression increases autophagosome formation, while knockdown of ANXA2 or BiP disrupts MAMs architecture and reduces mitophagosome numbers (n = 3). Scale bar = 200 nm. (I) Schematic of ER–mitochondria contact based on TEM images, showing enhanced contact in TMEM215 overexpressing cells, whereas ANXA2 or BiP knockdown weakens this contact (n = 3). (J,K) PLA staining (J) and quantification of PLA signal intensity (K) showing that TMEM215 overexpression enhances ER–mitochondria coupling signals, with more red signals of IP3R–GRP75 colocalization, while ANXA2 or BiP knockdown decreases these signals. Data are shown as the number of signal dots per cell (n = 10; one‐way ANOVA with Bonferroni post‐hoc test). Scale bar = 10 µm. (L,M) Rhod‐2 staining (L) and Rhod‐2 quantification (M) showing decreased mitochondrial Ca^2+^ levels following ANXA2 or BiP depletion (n = 3). Scale bar = 10 µm. (N) WB analysis demonstrating that overexpression of TMEM215, ANXA2, or BiP enhances mitophagy, whereas ANXA2 or BiP knockdown significantly impairs TMEM215‐induced mitophagy (one‐way ANOVA with Bonferroni post‐hoc test). (O) WB analysis showing that TMEM215, ANXA2, or BiP overexpression suppresses apoptosis and enhances cell survival signaling, effects dependent on mitophagy as evidenced by their reversal with Mdivi‐1 (one‐way ANOVA with Bonferroni post‐hoc test). (P) WB showing that BiP overexpression produces the corresponding phenotype and is similarly corrected by Mdivi‐1, indicating a BiP–mitophagy axis in anoikis regulation (one‐way ANOVA with Bonferroni post‐hoc test). Data are expressed as mean ± SD. *P < 0.05; **P < 0.01; ***P < 0.001.

**FIGURE 9:**
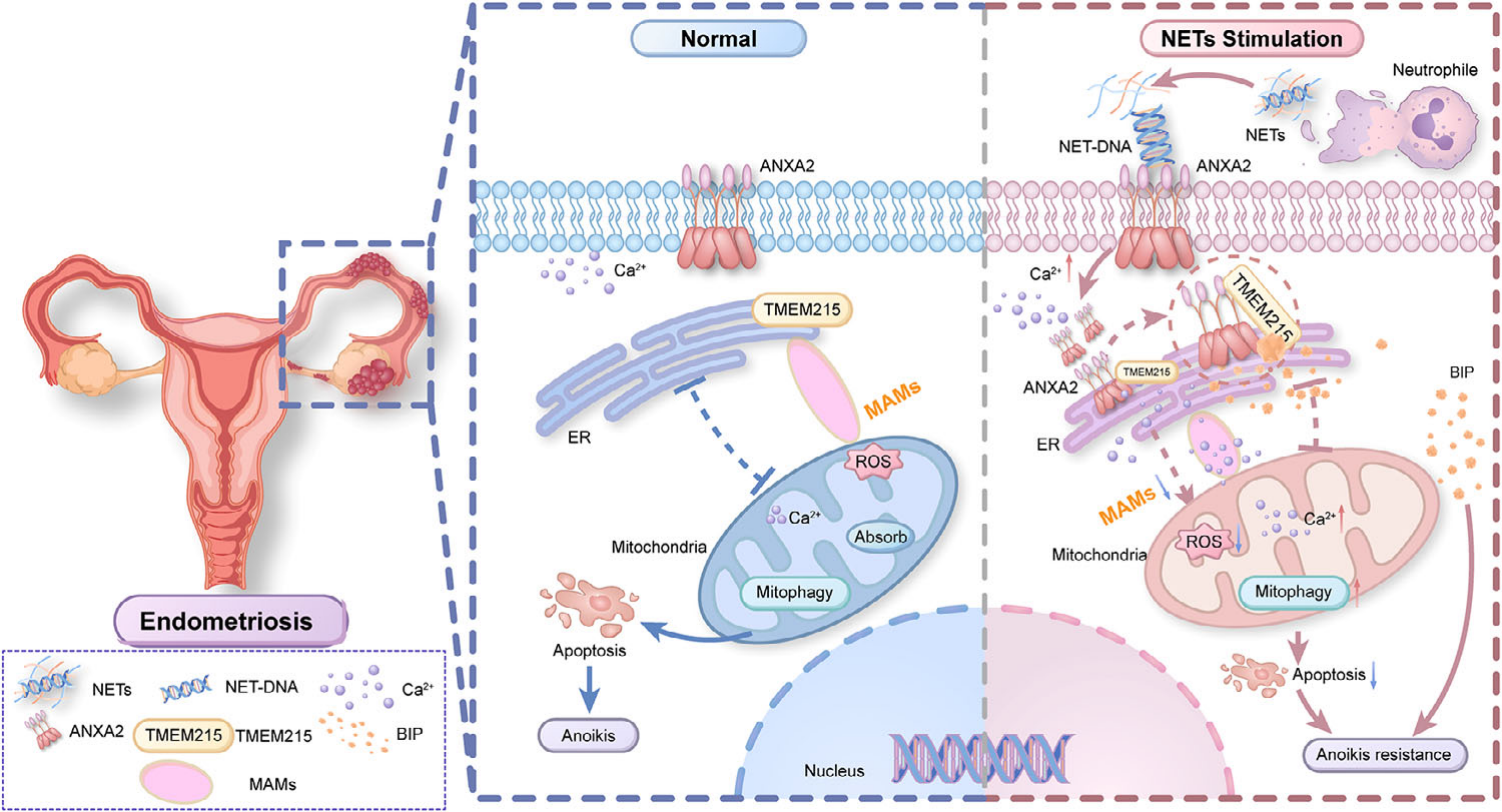
Schematic illustration of how NET‐DNA activates the ANXA2/TMEM215/BiP axis to enhance mitophagy and promote anoikis resistance in endometriosis.

To further clarify the upstream regulatory mechanisms involving TMEM215 in NET‐DNA signaling, IP‐MS analysis of TMEM215 immunoprecipitates identified the molecular chaperone BiP (GRP78) as a potential interacting partner (Figure 8A). Co‐immunoprecipitation experiments using a His‐tagged BiP construct confirmed the interaction between TMEM215 and BiP, showing clear binding bands in precipitates (Figure 8B). IF (Figure 8C) colocalization and PLA (Figure 8D) consistently demonstrated strong cytoplasmic proximity signals, indicating a close association between TMEM215 and BiP within the same cellular compartment. Molecular docking simulations further illustrated potential binding interfaces among TMEM215, ANXA2, and BiP, supporting the possibility of TMEM215–ANXA2–BiP complex formation (ANXA2: pink, BiP: purple, TMEM215: cyan) (Figure 8E). Notably, although ANXA2 overexpression or knockdown did not alter total BiP expression, it significantly affected their colocalization patterns (Figure S2E), suggesting the interaction depends on spatial proximity rather than transcriptional regulation. Pull‐down assays with ANXA2 truncation mutants identified the 266–339 aa region as essential for BiP binding (Figure 8F). In cells overexpressing TMEM215, colocalization of ANXA2 and BiP was markedly increased (Figure 8G), supporting a role for TMEM215 in promoting their spatial association. The knockdown and overexpression efficiencies of BiP were first confirmed (Figure S2F). To clarify how TMEM215, ANXA2, and BiP cooperatively regulate MAM integrity and mitophagy, we first examined ultrastructural alterations by TEM (Figure 8H). TMEM215 overexpression increased autophagosome formation and tightened ER–mitochondria contacts, whereas ANXA2 or BiP knockdown increased ER–mitochondria distance and reduced mitophagosome abundance (Figure 8I). We next assessed protein–protein proximity at MAMs using PLA. TMEM215 overexpression markedly enhanced IP3R–GRP75 interactions, whereas ANXA2 or BiP depletion diminished these PLA signals, further indicating impaired MAM stability (Figure 8J,K). Consistently, Rhod‐2 staining revealed reduced mitochondrial Ca^2^^+^ levels following ANXA2 or BiP knockdown (Figure 8L,M). Finally, Western blot analysis (Figure 8N) and its quantification (Figure S3D) showed that TMEM215, ANXA2, or BiP overexpression enhanced mitophagy, whereas ANXA2 or BiP silencing significantly impaired TMEM215‐induced mitophagy. At the cellular level, overexpressing TMEM215, ANXA2, or BiP reduced cleaved Caspase‐3 and increased Bcl‐2 expression, enhancing resistance to anoikis. These protective effects were abolished by the autophagy inhibitor Mdivi‐1 (Figure 8O; Figure S3E). Notably, blocking mitophagy with the mitochondrial fission inhibitor Mdivi‐1 largely negated the anti‐apoptotic effects of TMEM215 and ANXA2. Interestingly, BiP overexpression partially reversed the inhibitory effects of Mdivi‐1 and maintained reduced apoptosis levels (Figure 8P; Figure S3F). This suggests that BiP may protect cells through mechanisms that depend on mitophagy as well as those independent of mitophagy. Based on these data, BiP appears to be associated with both mitophagy‐related changes and apoptosis regulation in EESCs. Collectively, these findings support that BiP associates with TMEM215 and ANXA2 and is linked to increased ER–mitochondria contacts, mitophagy‐related activity, and reduced detachment‐induced apoptosis. The TMEM215–ANXA2–BiP axis therefore, represents a candidate molecular link between mitophagy‐related changes and apoptosis regulation in NET‐DNA‐treated EESCs (Figure 9).

## 4 Discussion

EMs has long been considered primarily a pelvic gynecological disorder. However, accumulating evidence indicates that EMs also exhibits systemic features, including chronic inflammation and autoimmune comorbidities, affecting a significant proportion of reproductive‐age women worldwide. Its pathogenesis involves multiple factors. Recent molecular and genetic studies revealed that abnormal hormonal receptor signaling, sustained inflammatory activation, and immune dysregulation collectively drive disease onset and progression [22].

Increasing attention has recently focused on NETs, which mediate tissue damage and immune imbalance in various disorders, including pulmonary, cardiovascular, and renal diseases [23, 24, 25]. These findings suggest that NETs play an important pathogenic role in chronic inflammation‐related conditions. Given the inflammatory microenvironment in EMs, NETs likely contribute to its pathophysiology. In this study, we systematically investigated the impact of NET‐DNA, the structural scaffold of NETs, on EMs progression and explored the underlying molecular mechanisms. Our data support that NET‐DNA enhances mitophagy‐related activity and is associated with anoikis resistance in EMs through an ANXA2–TMEM215‐associated pathway. Specifically, NET‐DNA binds ANXA2, promotes its translocation from the plasma membrane to the cytoplasm, and enhances its interaction with TMEM215. These changes were accompanied by increased ER–mitochondria contacts and improved cell survival under detachment stress. These findings provide new molecular insights into EMs pathogenesis, highlighting the importance of the NET‐DNA–ANXA2–TMEM215 axis in regulating mitophagy and anoikis resistance (Figure 9).

Neutrophils defend against pathogens through phagocytosis, degranulation, and NET formation [26, 27]. NETs consist of extracellular DNA fibers decorated with histones and granule proteins such as MPO, neutrophil elastase, and cathepsin G [26, 27]. Although NETs have crucial antimicrobial functions by trapping and neutralizing pathogens [28, 29], excessive or persistent NET formation can exacerbate autoimmune and inflammatory responses, aggravating noninfectious diseases [30]. Consistent with previous reports [31], our results showed significantly elevated circulating levels of NET biomarkers (Serum MPO and cf‐dsDNA) in EMs patients compared with healthy controls. IF analysis revealed notably increased NET formation in EC tissues relative to EN tissues. By constructing a time‐course EMs mouse model (days 7, 14, 21, and 28), we observed a clear shift in immune cell dynamics: neutrophils dominated the peritoneal cavity during the earlier phase that could be consistently analyzed (days 7–14), while T‐cell infiltration became prominent in later stages. IF analysis of ectopic lesions confirmed strong NET formation specifically during this interval (days 7–14), aligning with the immune composition of peritoneal lavage fluid. We further examined the very early post‐implantation window (days 1–5), during which lesions were already detectable, supporting the involvement of NETs‐associated events at an early stage of lesion development. However, lesions obtained on days 1 and 3 were extremely limited, and day 5 lesions were not sufficiently stable for the full range of downstream mechanistic analyses used here. Therefore, the most robust mechanistic evidence in the present study was obtained from the day 7–14 interval, which represents the early stage of detectable lesion establishment in our model. These results echo recent studies [32, 33, 34, 35, 36] indicating that NET‐DNA can interact with membrane‐associated proteins in diseases such as hepatocellular carcinoma and cardiac injury, thereby influencing driving disease progression. However, the mechanistic role of NET‐DNA in EMs remained unclear. To address this, we established an EMs mouse model and enhanced NET formation via intraperitoneal LPS injection, followed by DNase I treatment to degrade extracellular DNA. Eliminating NET‐DNA significantly attenuated LPS‐induced lesion growth, supporting a functional contribution of extracellular DNA to lesion progression in this model. In addition, repeated washing during NET‐DNA preparation, DNase I sensitivity, and downstream protein validation together argue against major contributions from residual NET‐associated proteins, PMA‐derived components, or other inflammatory contaminants. Collectively, these findings support that NET‐DNA is more than a passive inflammatory byproduct. NET‐DNA directly influences cellular behavior in the ectopic endometrial microenvironment, triggering ANXA2 translocation, TMEM215 activation, and MAM remodeling, thus correlating with promoting mitophagy and anoikis resistance in EESCs.

Mitophagy is a selective mechanism that eliminates dysfunctional mitochondria, preventing the harmful accumulation of damaged organelles within cells [37]. Under physiological conditions, mitochondrial homeostasis depends on the balance among mitochondrial biogenesis, fusion‐fission dynamics, and mitophagy‐mediated degradation of impaired mitochondria [38, 39]. When mitophagy is impaired, mitochondrial dysfunction contributes to various pathological conditions, including neurodegeneration, heart failure, cancer, and aging [40]. Recent studies have shown a close link between EMs and mitophagy, regulated through complex signaling pathways [41, 42, 43]. Consistent with these findings, our previous data also demonstrated increased mitophagic activity in endometriotic lesions [44]. Interestingly, the role of NETs in autophagy regulation appears context‐dependent. In ischemia‐reperfusion injury models, NET formation suppresses autophagy and exacerbates mitochondrial dysfunction, causing decreased membrane potential, excessive ROS production, reduced ATP synthesis, and enhanced mitochondrial fission [45]. Similarly, in atrial fibrillation, NETs induce autophagy‐associated apoptosis in cardiomyocytes by promoting mitochondrial depolarization and oxidative stress [46]. By contrast, our findings indicate that in EMs, a chronic inflammatory condition, NETs exert an opposite effect. NET‐DNA significantly enhances mitophagy and improves mitochondrial function in EESCs, evidenced by increased mitochondrial membrane potential, tighter ER–mitochondria contacts, elevated mitochondrial Ca^2+^ uptake, and reduced ROS levels. These results suggest that NET‐DNA–induced mitophagy and mitochondrial coupling cooperatively sustain cell survival under detachment stress. Additionally, DNase I–mediated NET‐DNA degradation significantly attenuated mitophagy, supporting NET‐DNA as an important upstream contributor to this process. Differences between our findings and those from acute cardiac injury models likely reflect distinct cellular adaptations to NETs in different pathological microenvironments. Acute, high‐intensity NET exposure results in mitochondrial damage, autophagic failure, and cell death, whereas chronic, moderate NET‐DNA stimulation in EMs may instead induce adaptive, protective mitophagy. This adaptation may support mitochondrial function and apoptotic resistance, and ultimately promotes anoikis resistance in EESCs.

To identify the membrane‐associated binding partner mediating NET‐DNA signaling, we performed biotin‐labeled NET‐DNA pull‐down assays combined with mass spectrometry. These assays identified ANXA2 as a direct NET‐DNA‐binding protein, with the 105–175 amino acid region defined as the key binding domain. ANXA2, a calcium‐dependent phospholipid‐binding protein, has crucial roles in cytoskeletal remodeling, membrane trafficking, and signal transduction [47]. It reportedly regulates autophagy and apoptosis under various stress conditions [48, 49, 50], and its membrane‐to‐cytoplasm translocation contributes to pro‐migratory and anti‐apoptotic effects in tumors and inflammatory diseases [51]. Our results identify ANXA2 as a candidate NET‐DNA‐binding protein in EESCs, providing new mechanistic insights into NET‐DNA–mediated signaling. Notably, NET‐DNA did not alter total ANXA2 expression, but induced its redistribution from the membrane to the cytoplasm, where it colocalized with ER markers and stabilized MAMs. ANXA2 knockdown disrupted NET‐DNA–induced mitophagy, decreased membrane potential, and loosened MAM architecture. Conversely, ANXA2 overexpression restored autophagic flux and enhanced cell survival. Thus, ANXA2 acts as a key mediator linking extracellular NET‐DNA exposure to intracellular mitophagy‐related responses. Unlike previously reported NET‐DNA receptors such as TLR9 or CCDC25, which primarily mediate inflammatory or death‐related signaling [34, 52], our data suggest that different NET‐DNA‐binding partners may engage distinct downstream responses depending on cellular context.

TMEM215, an ER transmembrane protein with two transmembrane domains and a luminal C‐terminal tail [53], participates in regulating membrane contacts and transmembrane signaling. In endothelial cells, TMEM215 knockdown triggers apoptosis via BiP‐mediated activation of the pro‐apoptotic protein BIK. This event is accompanied by increased MAM contacts, mitochondrial Ca^2+^ influx, and ER stress [54]. These changes occur under physiological laminar shear stress, representing a homeostatic apoptotic mechanism distinct from chronic inflammatory signaling. By contrast, our study demonstrated a distinct association of TMEM215 with survival‐related responses under NET‐DNA‐driven chronic inflammatory conditions. NET stimulation significantly increased TMEM215 expression and induced ANXA2 translocation to the cytoplasm, where the two proteins formed a detectable complex. Proteomic analysis revealed interactions of TMEM215 with both ANXA2 and BiP, with binding sites adjacent to the Ca^2+^‐binding domain of ANXA2, suggesting a link between this complex and Ca^2+^‐related signaling. Unlike the pro‐apoptotic phenotype observed in endothelial cells following TMEM215 depletion, TMEM215 upregulation in EESCs promoted mitophagy and anoikis resistance despite increased mitochondrial Ca^2+^ levels and tighter MAM contacts. This indicates context‐dependent signaling reprogramming, where TMEM215 may participate in coupling Ca^2+^ signals from pro‐apoptotic into pro‐survival cues in chronic inflammatory conditions. Because TMEM215 was also associated with BiP, it is possible that ER‐associated chaperone signaling contributes to these responses, although whether TMEM215 directly modulates BiP‐dependent apoptosis in EESCs remains unresolved. We further speculate that ANXA2 translocation plays a critical role in this process. Upon binding TMEM215, cytoplasmic ANXA2 may help organize ER‐associated signaling microdomains, ensuring controlled Ca^2+^ transfer between the ER and mitochondria. This controlled transfer activates autophagy without causing Ca^2+^ overload and apoptosis. Such adaptive remodeling of MAMs and Ca^2+^ homeostasis represents an EESCs survival response to sustained inflammatory stress. TMEM215 therefore emerges as a candidate scaffold for the ANXA2–BiP complex and a potential coordinator of Ca^2+^ signaling, autophagy, and apoptosis, although the precise molecular sequence requires further clarification.

Anoikis is a programmed cell death process triggered by loss of cell–matrix or cell–cell interactions. It involves both intrinsic (mitochondrial) and extrinsic (death receptor) pathways, ultimately activating nucleases and causing DNA fragmentation. In EMs, EESCs survive following detachment from the endometrial matrix, forming ectopic lesions and demonstrating strong anoikis resistance. In some patients, ectopic implants even occur in distant organs such as the thoracic cavity, abdominal wall, or brain [2], reflecting their exceptional survival and colonization capabilities in the absence of extracellular support. Previous studies identified multiple molecular pathways contributing to anoikis resistance, such as restoration of enhancer acetylation via p300 inhibition in ARID1A‐mutant epithelial cells [55], or promotion of anoikis through the serine protease inhibitor kallistatin [12]. Our findings extend these observations, showing that NET‐DNA activates the ANXA2–TMEM215–BiP axis, which is associated with increased mitophagy‐related activity and improved EESCs survival under detachment stress. This process stabilizes mitochondrial function, downregulates apoptotic signals, and partially sustains adhesion‐related signaling (integrin–FAK–SRC). Thus, autophagy and metabolic regulation indirectly maintain cell viability under conditions of impaired adhesion.

In summary, our findings support a model in which NET‐DNA regulates mitochondrial adaptation and energy metabolism via the ANXA2–TMEM215–BiP axis, suppressing mitochondria‐dependent apoptosis and conferring anoikis resistance to EESCs. This mitophagy‐driven survival mechanism may help explain the persistence and implantation of ectopic endometrial cells, potentially contributing to recurrence and distant metastasis‐like dissemination observed in EMs. It should be noted that BiP's dual roles in autophagy and apoptosis regulation remain incompletely characterized, partly due to the lack of specific mutants or localization tools, and require further investigation.

## 5 Conclusion

Our findings collectively suggest that NET‐DNA is associated with the ANXA2/TMEM215/BiP signaling pathway, which correlates with altered mitochondrial regulation in EESCs. This pathway was associated with ANXA2 translocation from the membrane to the cytoplasm, increased TMEM215–BiP association, and stabilized ER–mitochondria contacts. These changes were accompanied by increased mitophagy‐related activity and reduced mitochondria‐dependent apoptosis, and were associated with anoikis resistance in EESCs under detachment conditions. These results highlight the potential relevance of the NET‐DNA–ANXA2/TMEM215/BiP axis in the pathophysiology of EMs. They also provide novel molecular insights into how chronic inflammatory signaling may influence cellular stress responses and promote the persistence of ectopic cells.

## 6 Study Limitations

This study possesses significant strengths; however, certain limitations warrant attention. Firstly, while both human samples and mouse models were analysed, the clinical specimens were limited in number and obtained from a single centre. To enhance the generalisability of our findings, larger cohorts from multiple centres are required. Secondly, although we further examined the very early post‐implantation window (days 1–5), lesions obtained on days 1 and 3 were extremely limited, and day 5 lesions were not sufficiently stable for the full range of downstream molecular and ultrastructural analyses used in this study. Therefore, the most robust mechanistic evidence remains concentrated in the day 7–14 interval. Thirdly, although NET‐DNA itself is a nucleic acid component of NETs rather than an endotoxin, endotoxin levels in the purified NET‐DNA preparations were not directly measured in the current study. Repeated washing and purification, together with the PMA‐only mock control and purity assessment, reduced the likelihood that the observed effects were driven by non‐DNA contaminants; however, trace endotoxin contamination cannot be formally excluded. Fourthly, although BiP was identified as part of the TMEM215‐ANXA2‐BiP complex, which was associated with increased ER–mitochondria contacts and mitophagy‐related activity, it also appeared to exert anti‐apoptotic effects that were not fully explained by mitophagy alone. The current study did not fully resolve whether this effect reflects ER stress‐related signaling, compartment‐specific functions, or other mitophagy‐independent mechanisms. In addition, although previous studies have suggested that BiP may interact with pro‐apoptotic proteins such as Bax and BIK, these mechanisms were not directly addressed here. Therefore, the relative contributions of BiP's role in mitophagy regulation versus its potential anti‐apoptotic activity remain unclear. Future research employing BiP mutants that disrupt specific interaction domains or alter subcellular localisation will help distinguish between these mechanisms. These limitations underscore the need for more comprehensive clinical validation and further mechanistic dissection to fully elucidate BiP's complex role in NET‐DNA‐driven survival signalling in endometriosis.

## Author Contributions

**Honglin Wang**: conceptualization, methodology, investigation, data curation, formal analysis, visualization, writing – original draft. **Yanling Gou**: clinical sample collection, histology and immunohistochemistry, data curation, investigation, writing – review and editing. **Huiyan Zhang**: methodology, investigation, animal experiments, data curation, validation, writing – review and editing. **Hongli Wang**: clinical sample coordination, patient data acquisition, sample handling. **Beidi Wang**: clinical data processing, sample validation, qRT‐PCR assistance. **Jinming Liu**: TEM sample preparation, ultrastructural analysis, image quantification. **Yingying Cao**: flow cytometry, immune profiling, data analysis. **Ruru Bai**: ELISA assays, serum biomarker analysis, sample processing. **Yuxin Zhao**: bioinformatics analysis, RNA‐seq processing, pathway enrichment. **Xu Han**: statistical analysis, figure preparation, data visualization. **Chao Feng**: assistance with cell experiments and animal experiments. **Xin Huang**: manuscript correction, animal husbandry, project support. **Zongfeng Zhang**: conceptualization, supervision, project administration, funding acquisition, writing – review and editing, final approval of the manuscript.

## Conflicts of Interest

All authors declare no conflicts of interest. The statement is correct as presented.

## Supporting information




**Supporting File 1**: advs75442‐sup‐0001‐SuppMat.docx.

**Supporting File 2**: advs75442‐sup‐0002‐Data.pdf.

**Supporting File 3**: advs75442‐sup‐0003‐TableS1.docx.

## Data Availability

The data that support the findings of this study are available from the corresponding author upon reasonable request.
