# Supplementary material for: NET‐DNA Activates the ANXA2/TMEM215/BiP Axis to Promote Mitophagy‐Mediated Anoikis Resistance in Endometriosis
Source: Adv Sci (Weinh). 2026 Apr 27;13(40):e75442. doi: 10.1002/advs.75442 (PMC13335634; doi:10.1002/advs.75442)
Supplement: Supplementary file 1 — Supporting File 1: advs75442‐sup‐0001‐SuppMat.docx. [file ADVS-13-e75442-s001.docx]

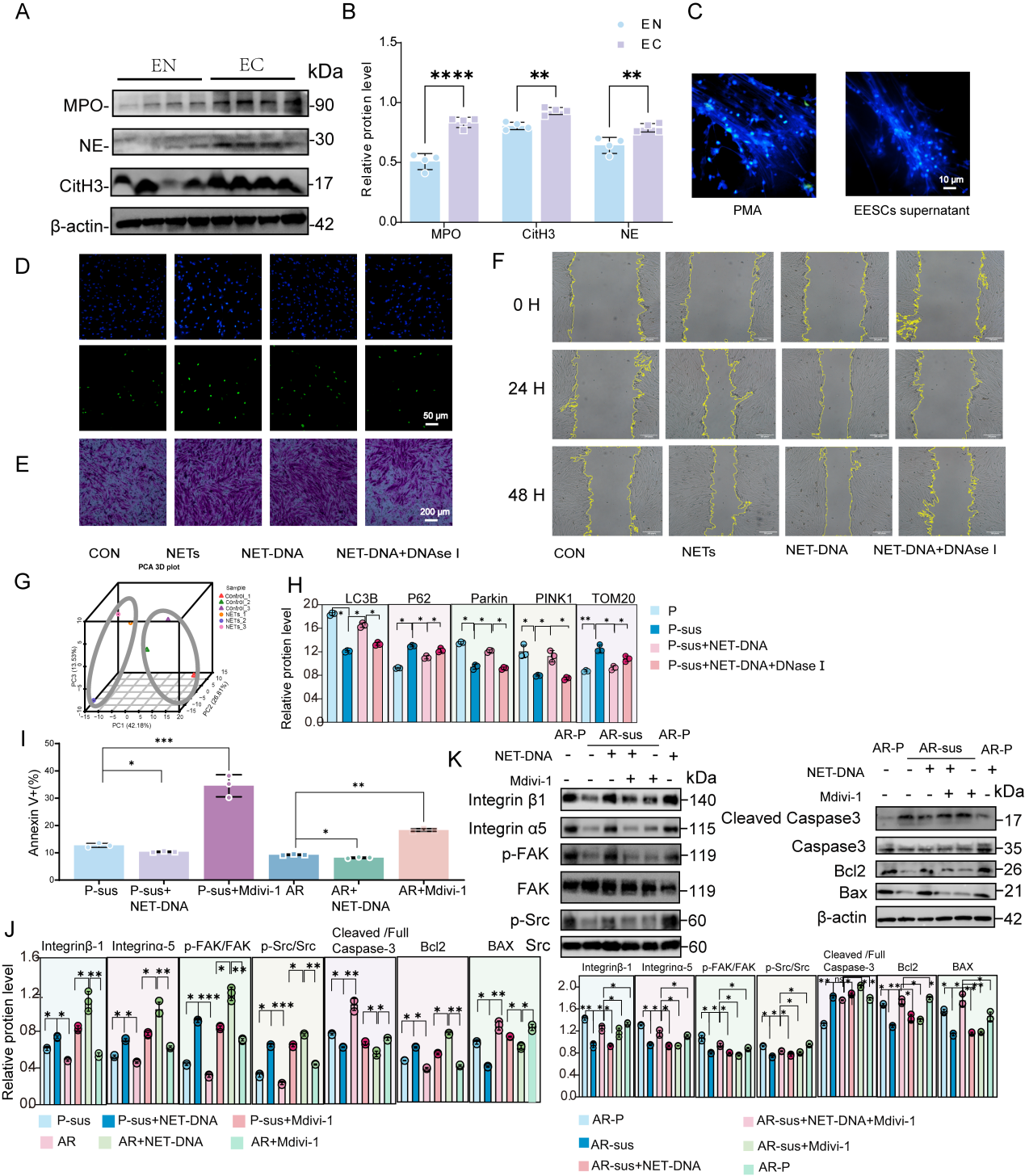


**Figure S1. Characterization of NETs and their effects on EESCs functions.**

(A) Western blot analysis showing higher expression of MPO, NE, and CitH3 in ectopic endometrium (EC) compared to normal endometrium (EN). (B) Quantification of MPO, CitH3, and NE confirming significantly elevated NET markers in EC tissues (n=4 per group, independent samples t-test). (C) Representative immunofluorescence (IF) images showing NET formation in PMA-stimulated neutrophils and in supernatants from EESCs cultured with NETs (DNA stained blue, Scale bar = 10 μm). (D) EdU staining showing that NETs and purified NET-DNA markedly enhance EESCs proliferation, whereas DNase I abolishes this effect (Scale bar = 50 μm). (E) Transwell assays showing that NETs and NET-DNA increase EESCs migration, while DNase I treatment reduces migration (Scale bar = 200 μm). (F) Wound-healing assays demonstrating that NETs and NET-DNA promote EESCs migration, and DNase I diminishes this enhancement. (G) PCA 3D plot of RNA-seq showing clear transcriptomic separation between control and NETs-treated groups. (H) Figure 3N: Western blot quantification of LC3B, P62, Parkin, PINK1, TOM20 protein levels, showing changes in autophagy and mitophagy markers after NET-DNA treatment in P-sus and AR cells, indicating that NET-DNA regulates cell function through autophagy/mitophagy pathways (n=3 per group, one-way ANOVA followed by Bonferroni post-hoc multiple comparisons test). (I) Figure 3O: Annexin V/PI flow cytometry analysis showing that NET-DNA reduces apoptosis and enhances anoikis resistance in P-sus cells, whereas Mdivi-1 increases apoptosis. AR cells exhibit intrinsic anoikis resistance, which is enhanced by NET-DNA and weakened by Mdivi-1 (n=3 per group, two-way ANOVA followed by Bonferroni test). (J) Figure 3P: Western blot analysis showing that NET-DNA restores integrin-FAK-Src signaling and suppresses apoptosis in AR cells, while Mdivi-1 reduces survival signaling and promotes apoptosis, indicating that NET-DNA-mediated protection depends on mitophagy (n=3 per group, two-way ANOVA followed by Bonferroni test). (K) Western blot showing that suspension culture decreases integrin-FAK-Src signaling and increases apoptosis in AR cells. NET-DNA restores survival signaling and suppresses apoptosis, while Mdivi-1 reduces survival signaling and promotes apoptosis, indicating that NET-DNA-mediated protection depends on mitophagy (n=3 per group, two-way ANOVA followed by Bonferroni test). All data are presented as means ± SD. **P* < 0.05, ***P* < 0.01, ****P* < 0.001.


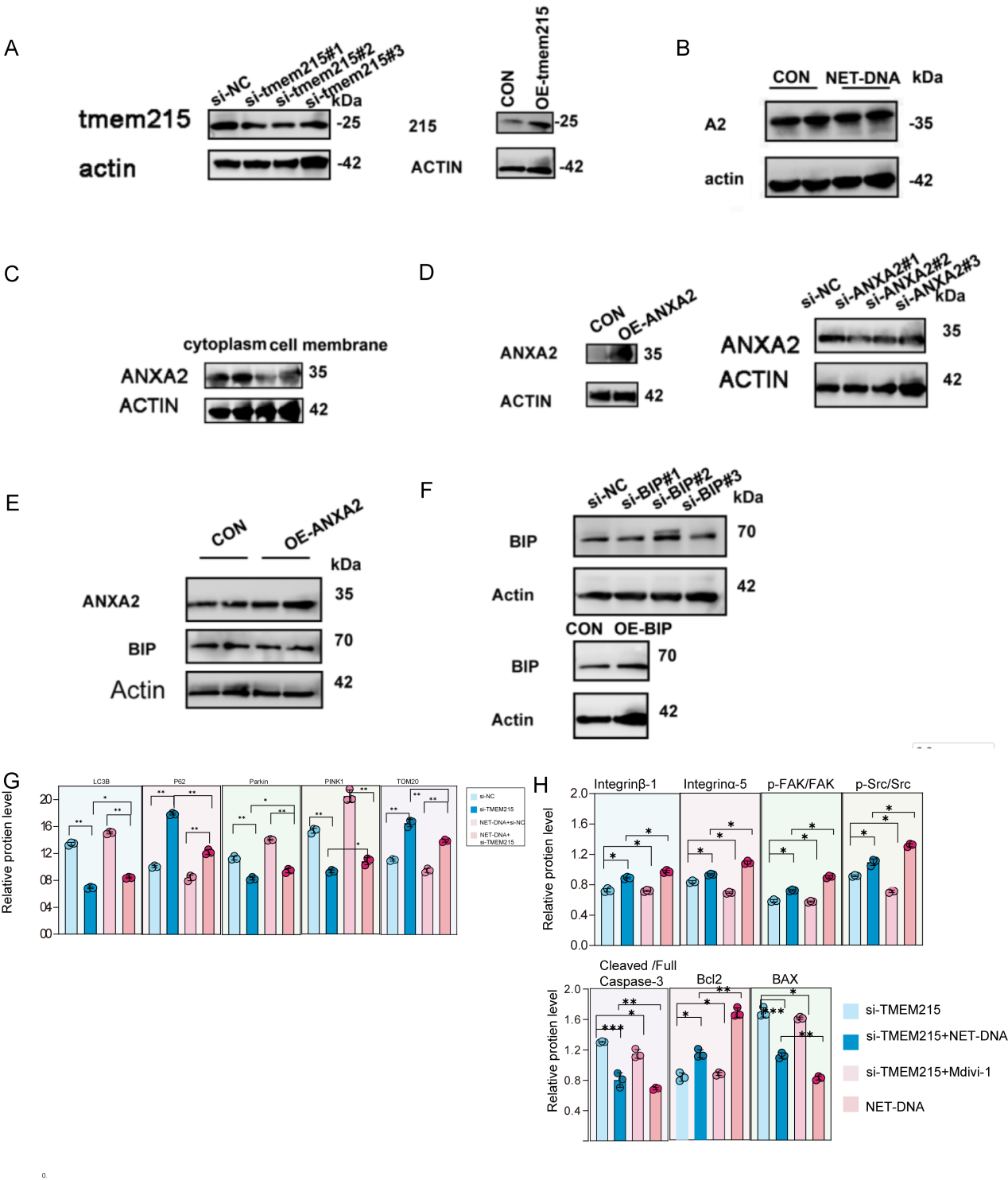


**Figure S2. Regulation of TMEM215, ANXA2, and BiP expression and localization.**

(A) WB confirming efficient TMEM215 knockdown by three siRNAs and successful overexpression by TMEM215-OE plasmid. (B) WB showing that NET-DNA does not alter total ANXA2 expression. (C) Membrane–cytoplasmic fractionation demonstrating that NET-DNA reduces membrane-localized ANXA2 and increases cytoplasmic ANXA2. (D) WB confirming effective modulation of ANXA2 expression by siRNAs and ANXA2-OE plasmid. (E) WB showing that ANXA2 overexpression does not change total BiP levels but increases ANXA2–BiP colocalization (supported by IF in main figures). (F) WB confirming efficient BiP knockdown by three siRNAs and successful BiP overexpression. (G) Figure 4K: Western blot analysis showing changes in LC3B, P62, Parkin, PINK1, and TOM20 protein levels, indicating the regulation of autophagy and mitophagy markers after different treatments (n=3 per group, one-way ANOVA followed by Bonferroni post-hoc multiple comparisons test). (H) Figure 4N: Western blot analysis showing the effect of NET-DNA, TMEM215 knockdown, and Mdivi-1 treatment on Integrin-β1, Integrin-α5, p-FAK/FAK, p-Src/Src, Cleaved Caspase-3, and BAX protein levels, indicating their impact on signaling pathways and apoptosis (n=3 per group, one-way ANOVA followed by Bonferroni post-hoc multiple comparisons test).All data are presented as means ± SD. **P* < 0.05, ***P*< 0.01, ****P* < 0.001.


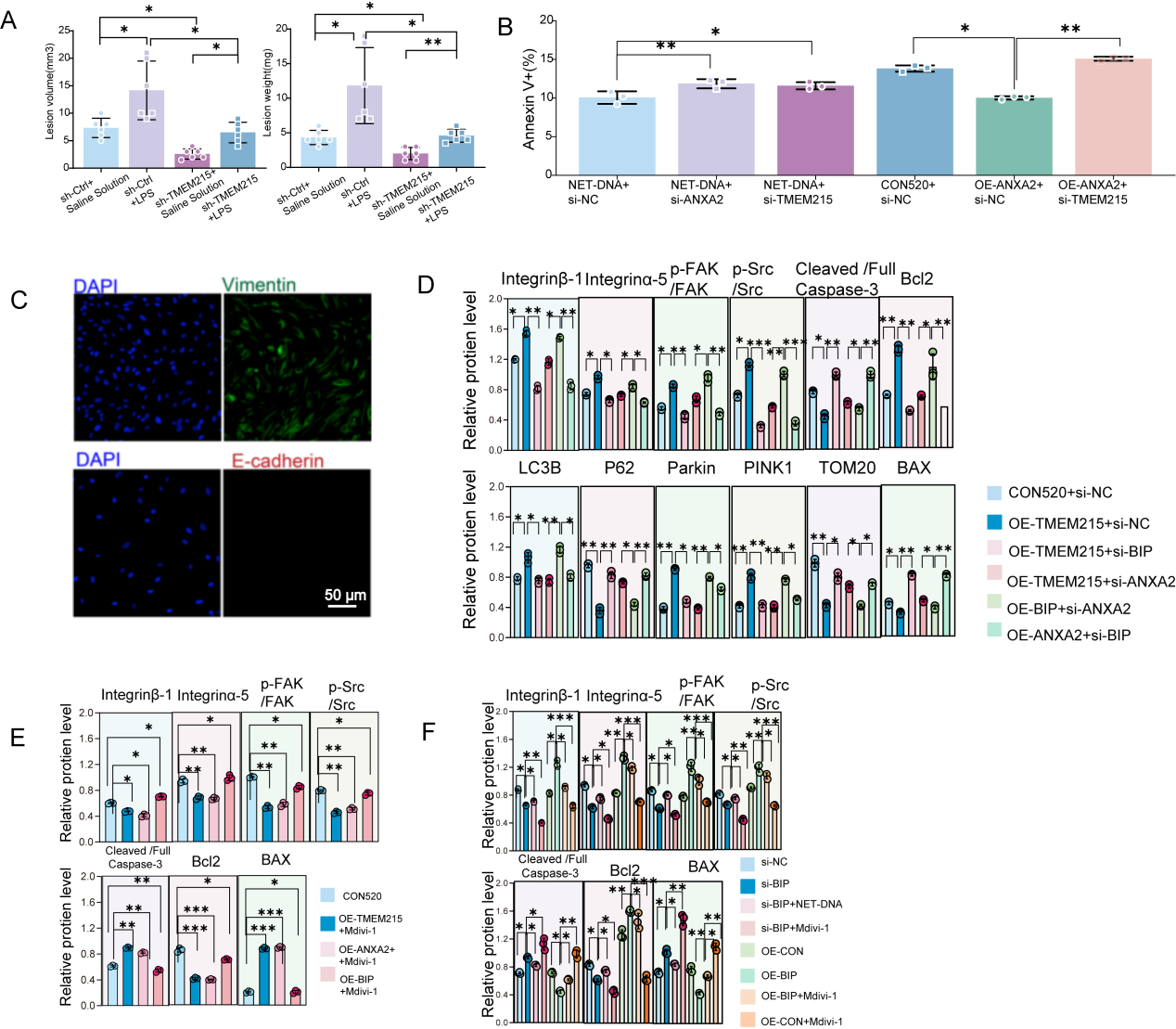


**Figure S3. In vivo validation of NET-related pathways and apoptosis regulation.** (A) Quantification of lesion volume and weight in sh-Ctrl + Saline Solution, sh-Ctrl + LPS, sh-TMEM215 + Saline Solution, and sh-TMEM215 + LPS groups (n=6 per group, one-way ANOVA followed by Bonferroni post-hoc multiple comparisons test).

(B) Flow cytometry analysis (Figure 7G) showing the effect of NET-DNA treatment and ANXA2/TMEM215 knockdown on EESC apoptosis (n=3 per group, one-way ANOVA followed by Bonferroni post-hoc multiple comparisons test). (C) Immunofluorescence staining of primary endometrial stromal cells showing strong Vimentin positivity and absence of E-cadherin signal, confirming the mesenchymal identity and high purity of the isolated stromal cells. (Scale bar = 50 μm). (D) Protein quantification (Figure 8N) showing the effects of NET-DNA, TMEM215 knockdown, and Mdivi-1 treatment on integrin-β1, integrin-α5, p-FAK/FAK, p-Src/Src, Cleaved Caspase-3, and BAX protein levels (n=3 per group, one-way ANOVA followed by Bonferroni post-hoc multiple comparisons test). (E) Protein quantification (Figure 8O) showing the effects of NET-DNA, BiP knockdown, and Mdivi-1 treatment on integrin-β1, integrin-α5, p-FAK/FAK, p-Src/Src, Cleaved Caspase-3, and BAX protein levels (n=3 per group, one-way ANOVA followed by Bonferroni post-hoc multiple comparisons test). (F) Protein quantification (Figure 8P) showing the effects of BiP knockdown and Mdivi-1 treatment on Cleaved Caspase-3, Bcl2, and BAX protein levels (n=3 per group, one-way ANOVA followed by Bonferroni post-hoc multiple comparisons test). All data are presented as means ± SD. **P* < 0.05, ***P*< 0.01, ****P* < 0.001.


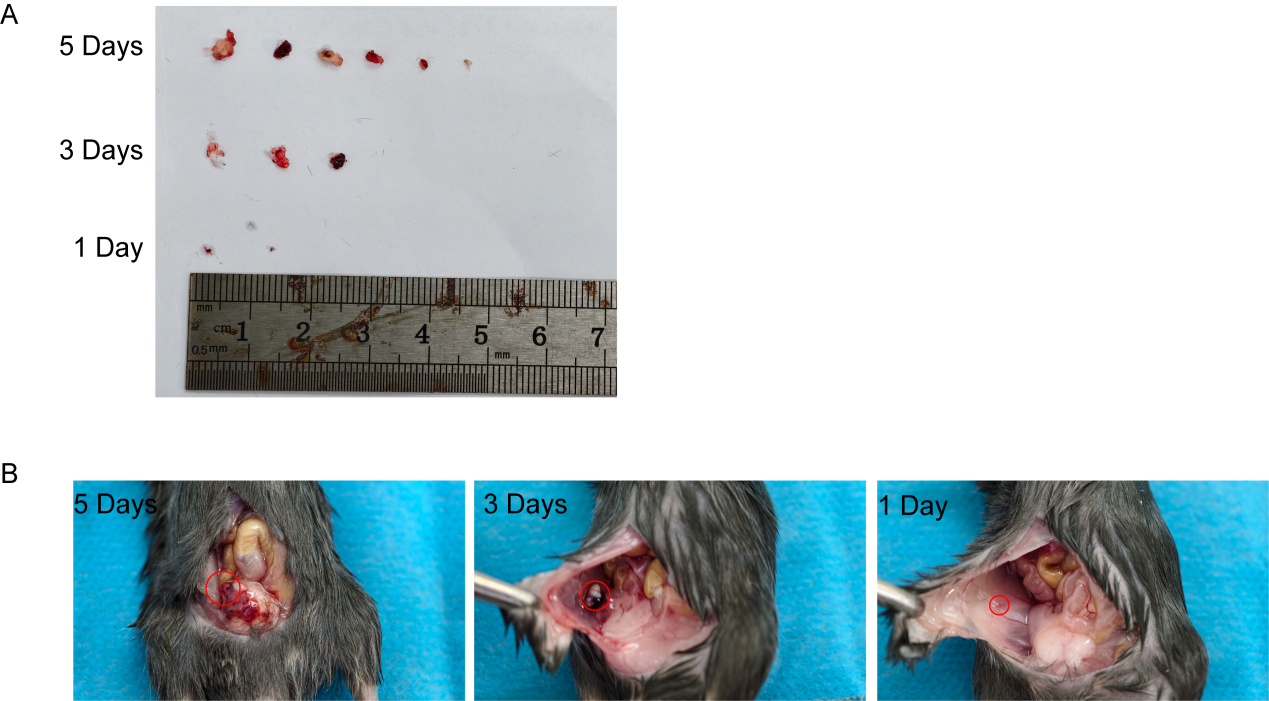


**Figure S4. In vivo evaluation of lesion formation in EMs mice at Day 1, Day 3, and Day 5.**

(A) Representative images of lesions from EMs mice at Day 1, Day 3, and Day 5 showing lesion size. (B) Photographs of lesion formation at Day 1, Day 3, and Day 5, highlighting the difficulty in obtaining sufficient lesion tissue for histological and mechanistic analysis at the early time points.


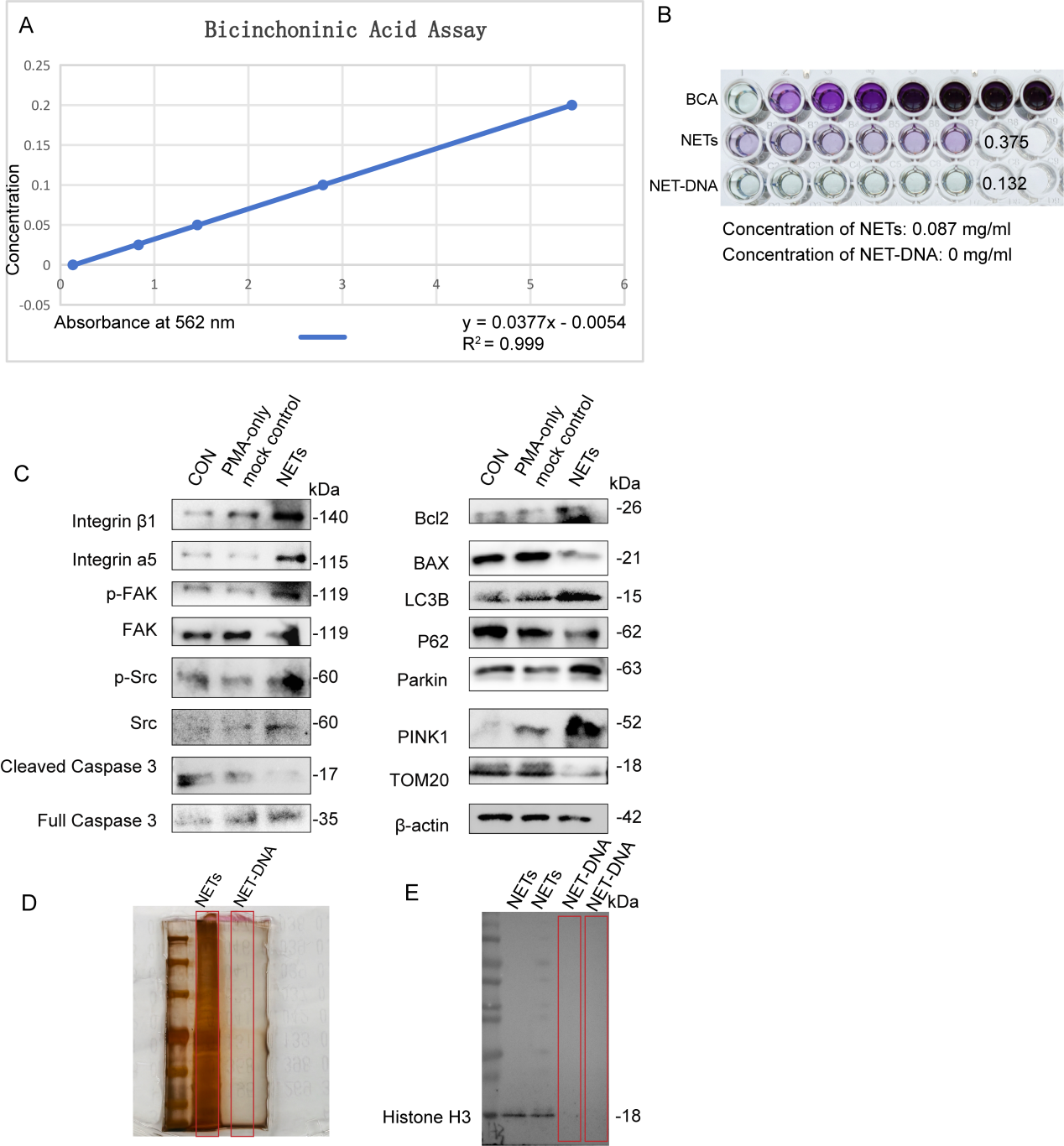


**Figure S5. Evaluation of protein concentration and the effect of NETs and NET-DNA on mitochondrial autophagy and anoikis resistance.**

(A) BCA curve showing the protein concentration measurement method. (B) Measurement of NETs and NET-DNA protein concentration, showing the concentrations of NETs (0.087 mg/ml) and NET-DNA (0 mg/ml). (C) Western blot analysis of the effects of control, PMA-only mock control, and NETs on downstream mitochondrial autophagy and anoikis resistance, showing changes in the expression of Integrin β1, Integrin α5, p-FAK, FAK, p-Src, Src, Cleaved Caspase-3, and Full Caspase-3. (D) Silver staining of NETs and NET-DNA to assess the presence of protein residues in NET-DNA. (E) Western blot analysis showing the presence of Histone H3 in NETs and NET-DNA, indicating if histone residues are present.
