# Supplementary material for: NET‐DNA Activates the ANXA2/TMEM215/BiP Axis to Promote Mitophagy‐Mediated Anoikis Resistance in Endometriosis
Source: Adv Sci (Weinh). 2026 Apr 27;13(40):e75442. doi: 10.1002/advs.75442 (PMC13335634; doi:10.1002/advs.75442)

Figure 3F First Group 1： CON CON NET-DNA NET-DNA

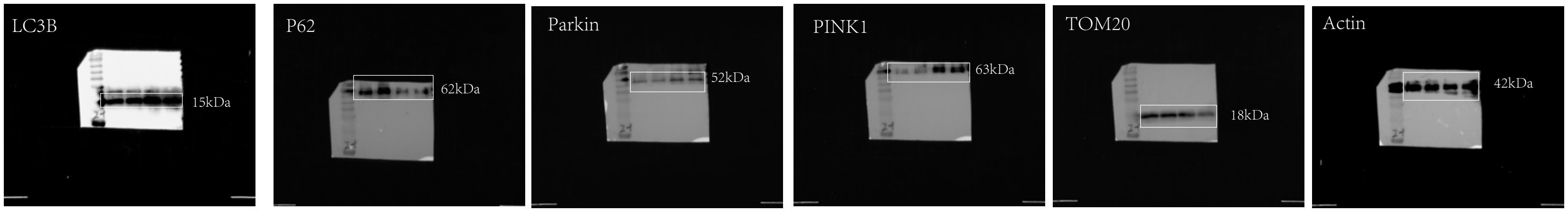

Repeat Group 2

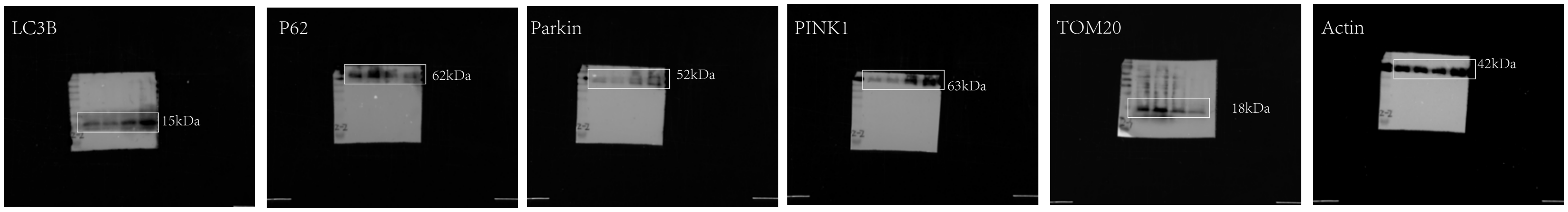

Repeat Group 3

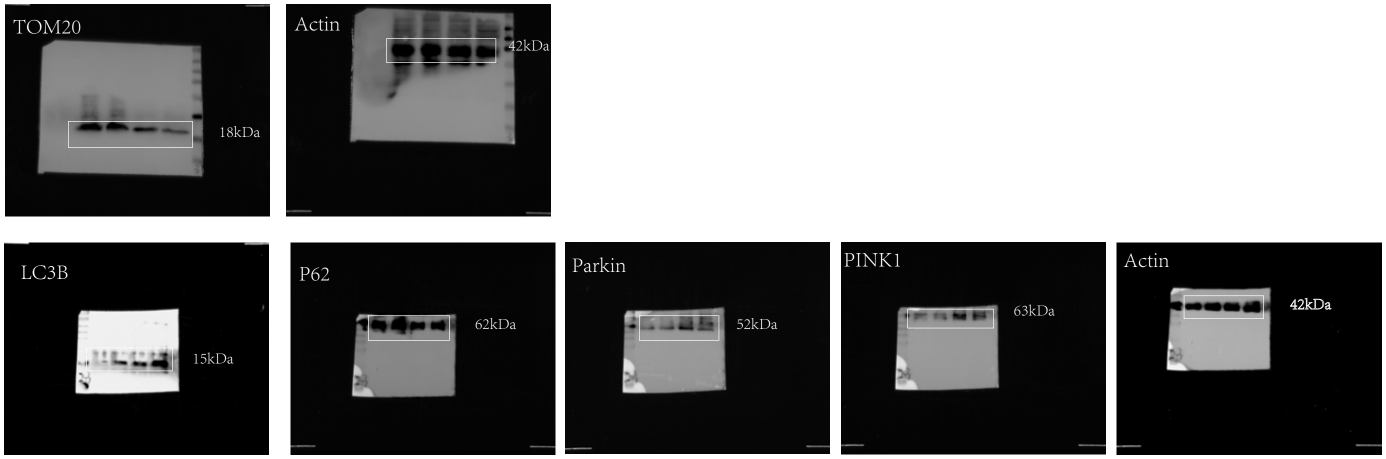

Figure 3H First Group 1： P、P+NET-DNA、sus、sus+NET-DNA、AR、AR+NET-DNA

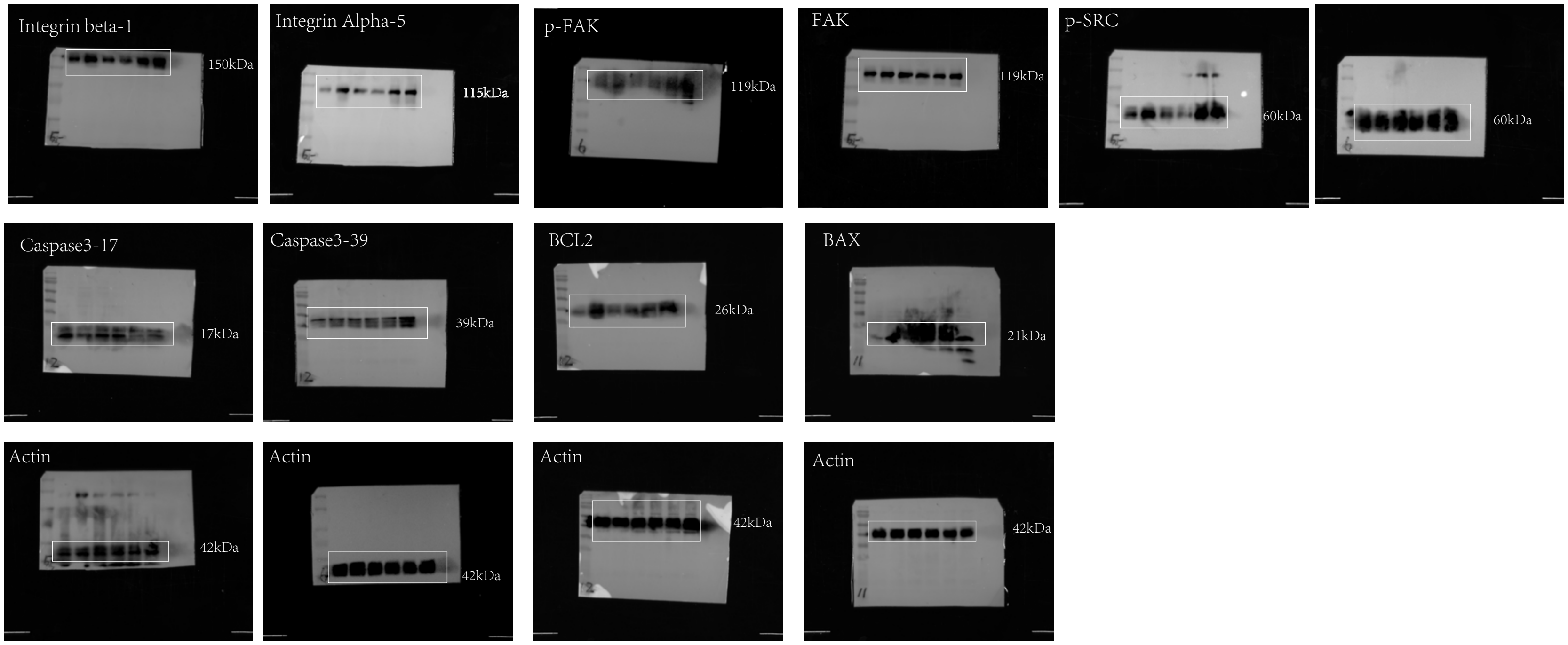

Repeat Group 2

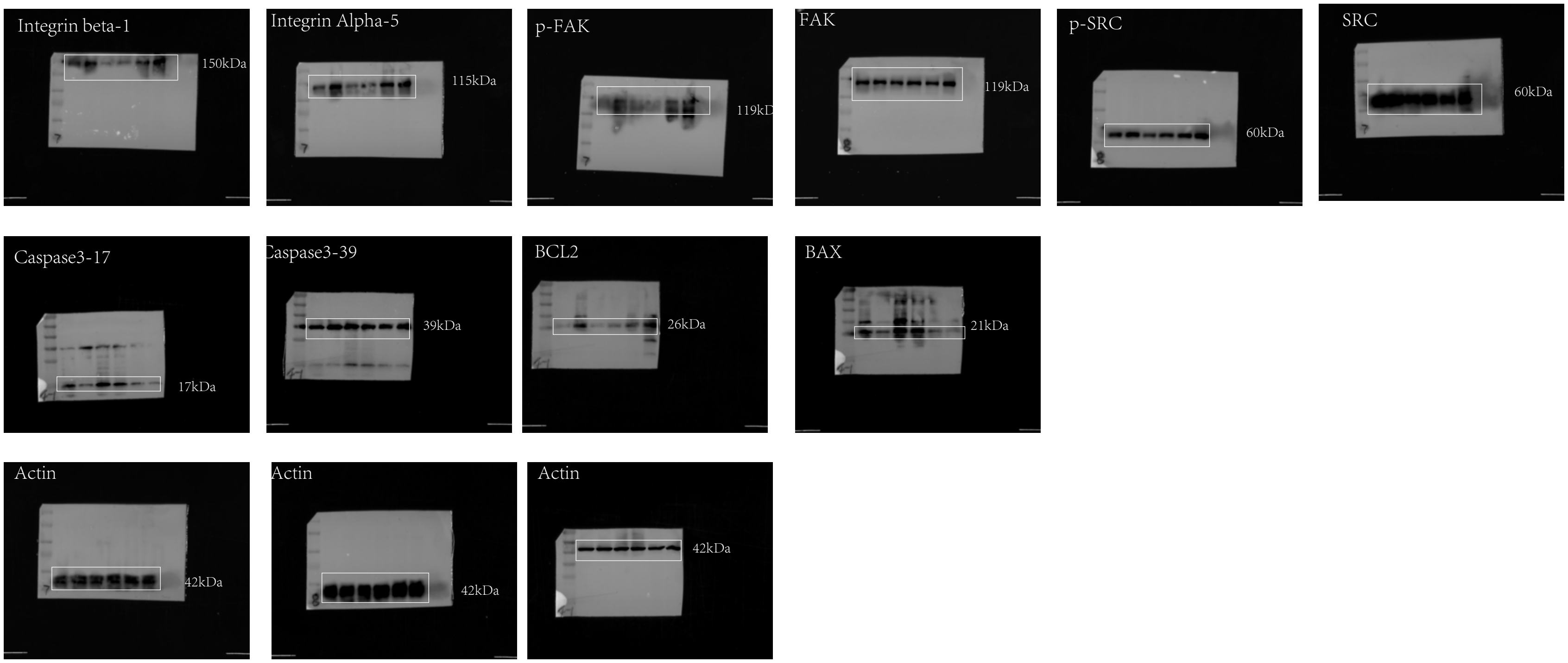

Repeat Group 3

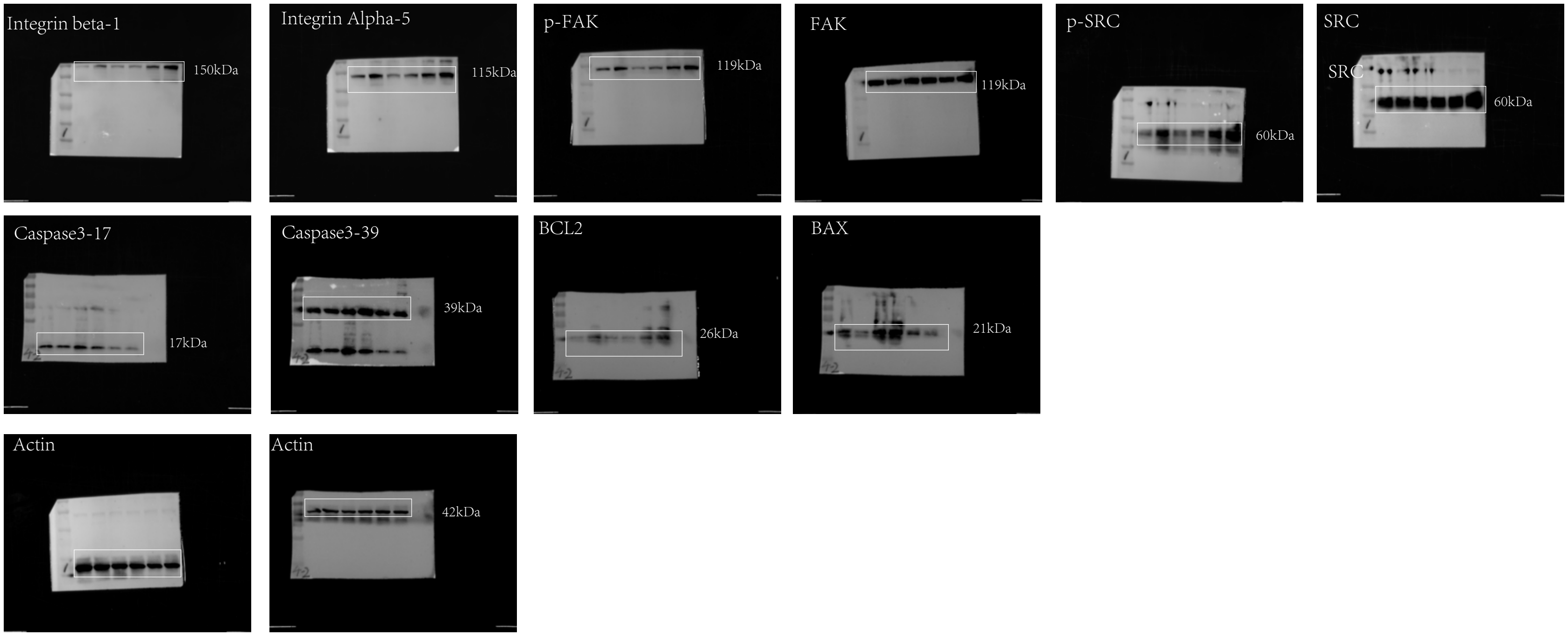

Figure 3J First Group 1： SUS 0、8、16、24、48、72h

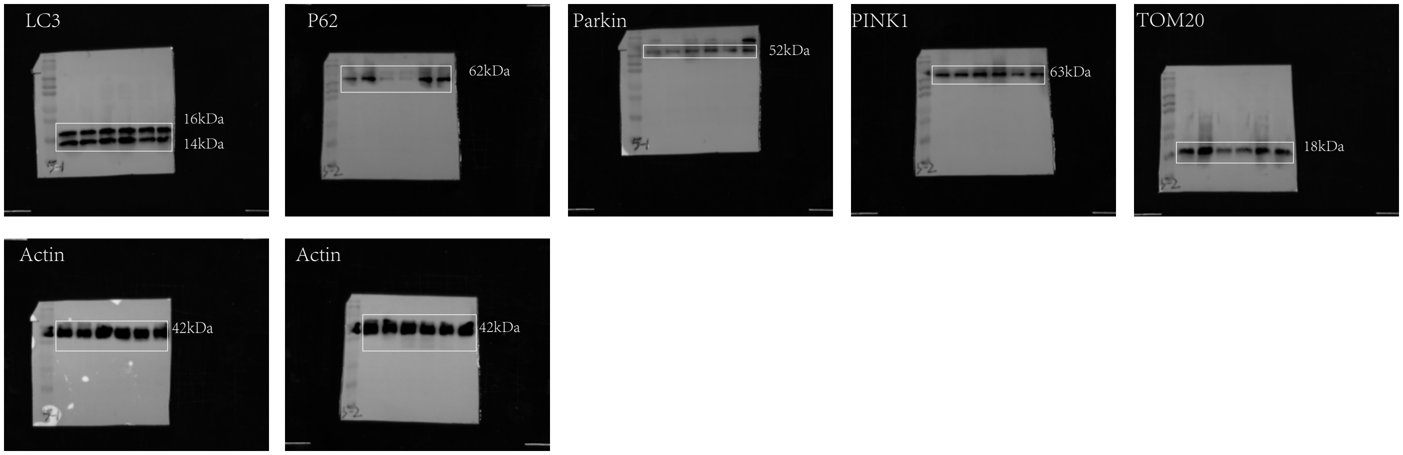

Repeat Group 2

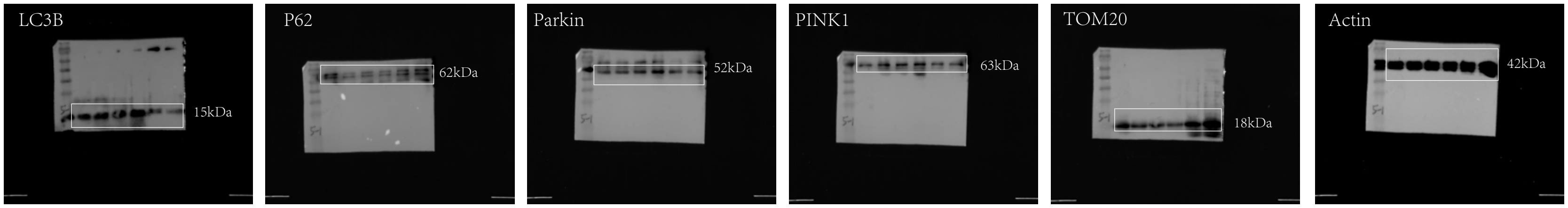

Repeat Group 3

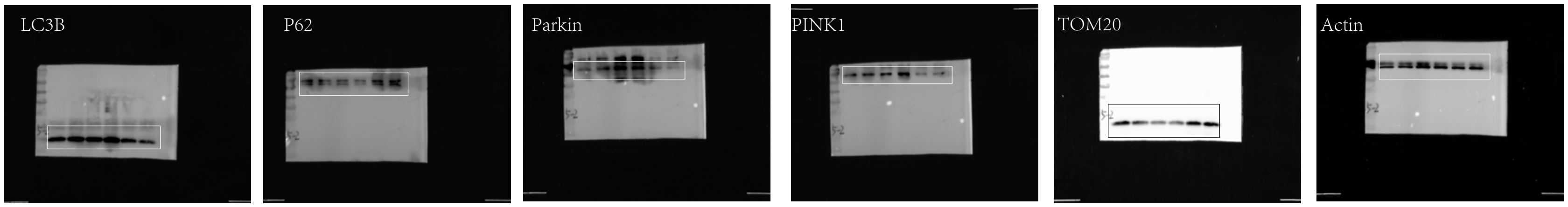

Figure 3N First Group 1： p、sus、sus+NET-DNA、sus+NET-DNA+DNaseI

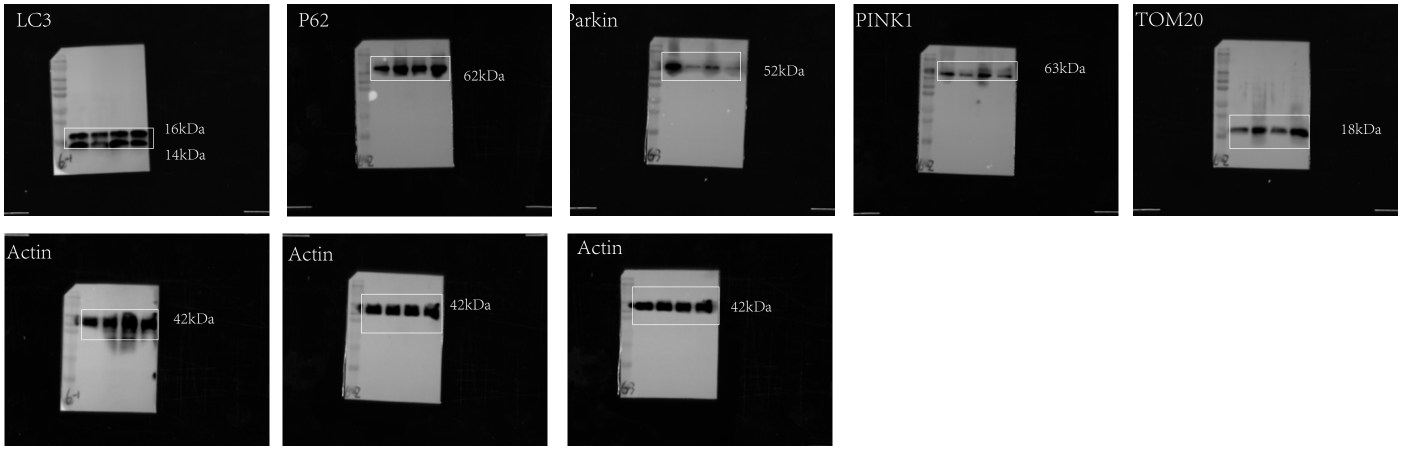

Repeat Group 2

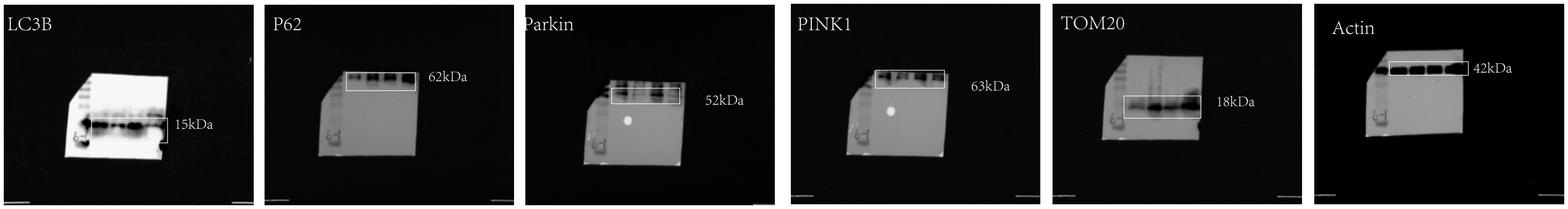

Repeat Group 3

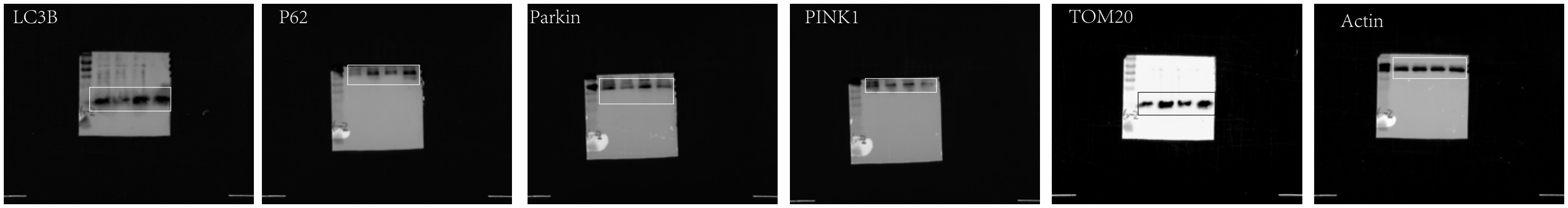

Figure 3P- First Group 1

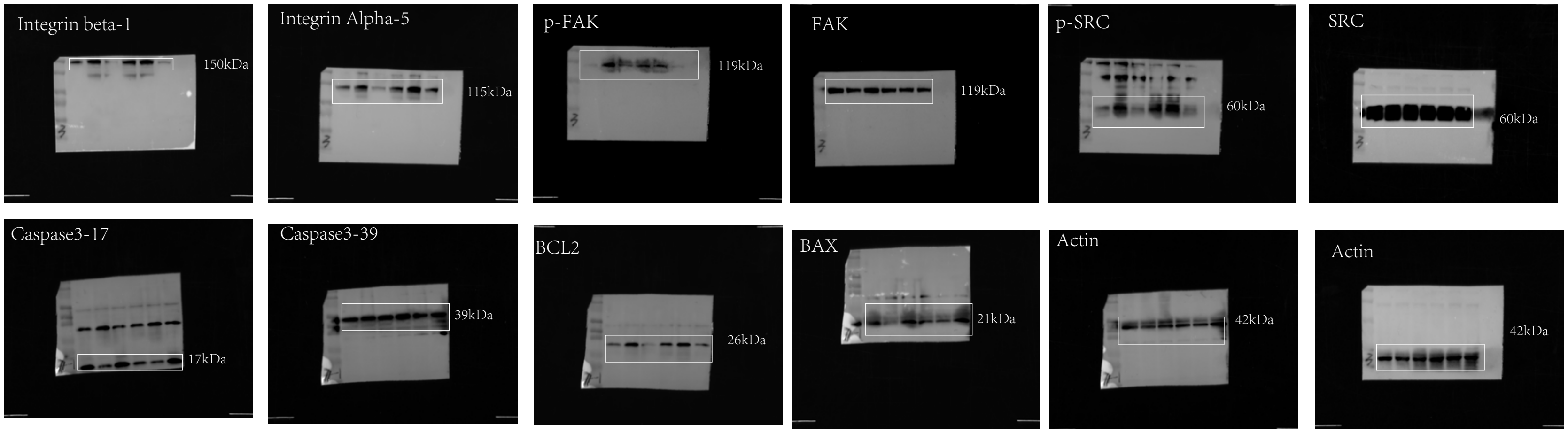

Repeat Group 2

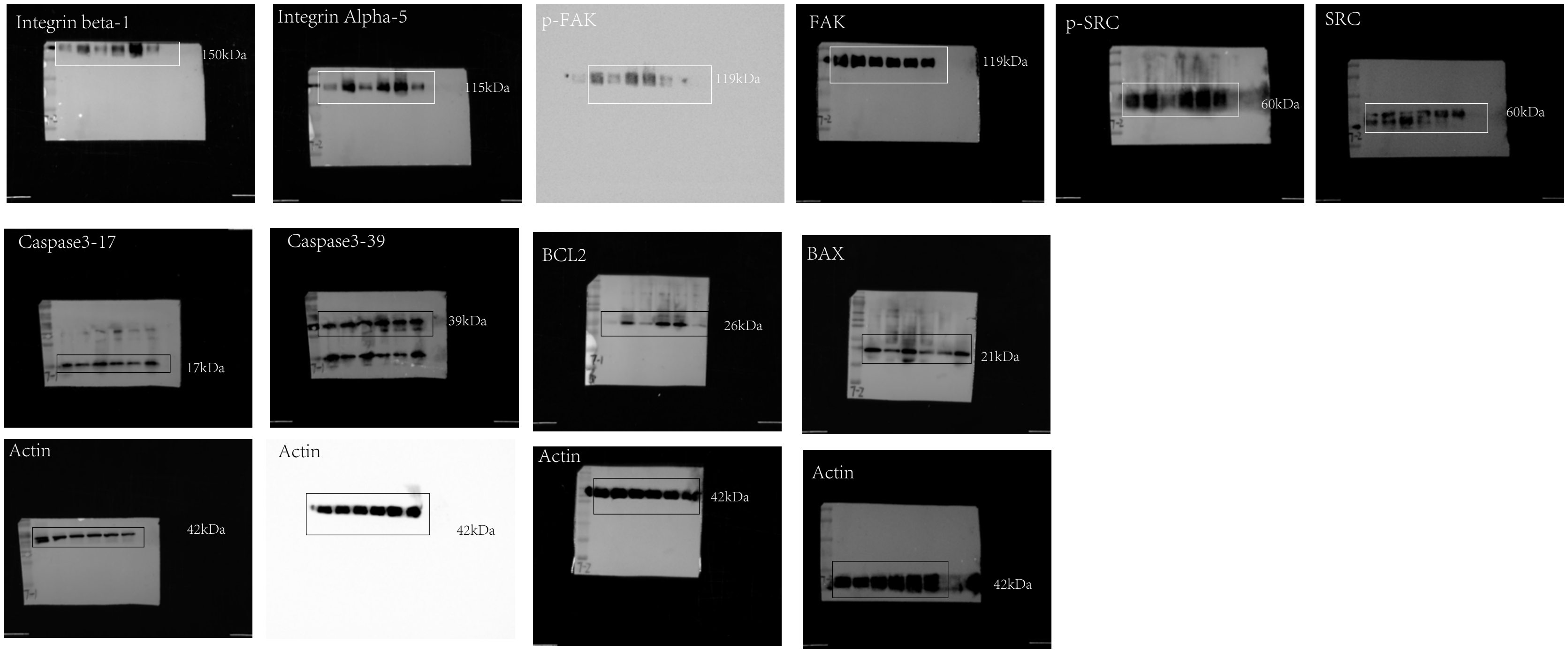

Repeat Group 3

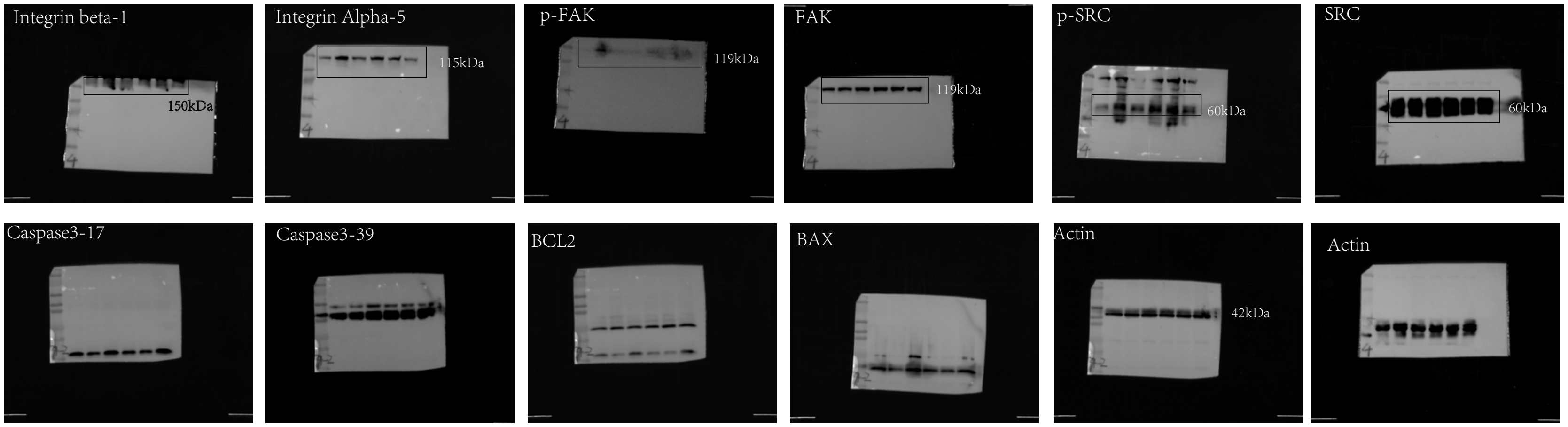

Figure 4B First Group 1： NET-DNA-TMEM215 Repeat Group 2 Repeat Group 3

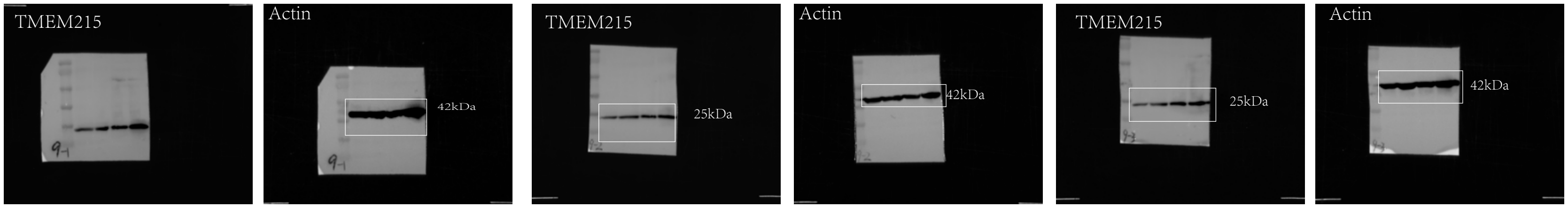

Figure 4K First Group 1

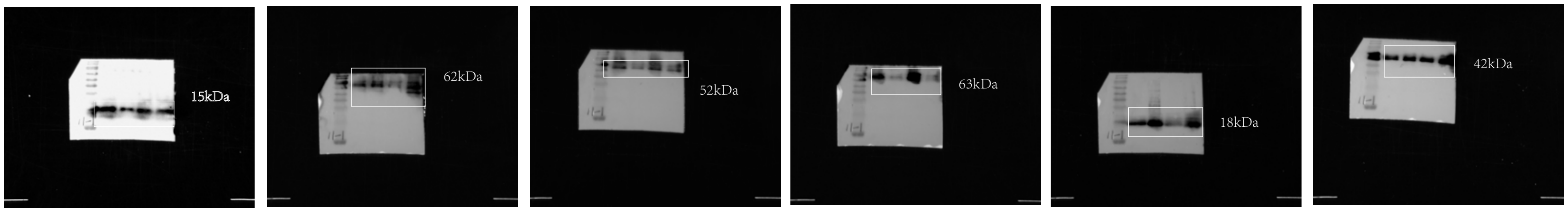

Repeat Group 2

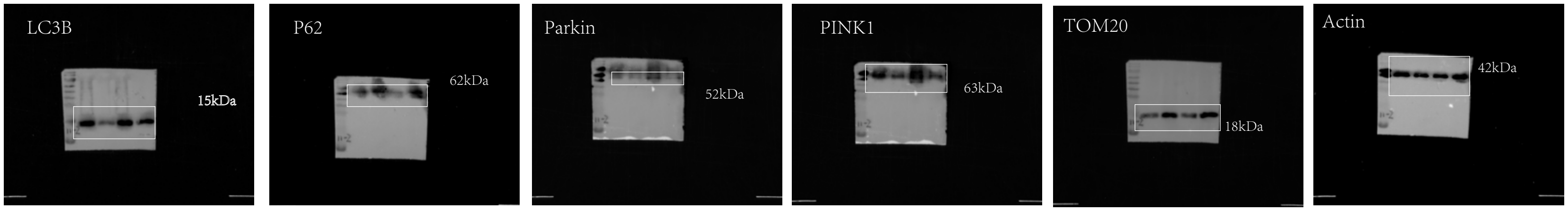

Repeat Group 3

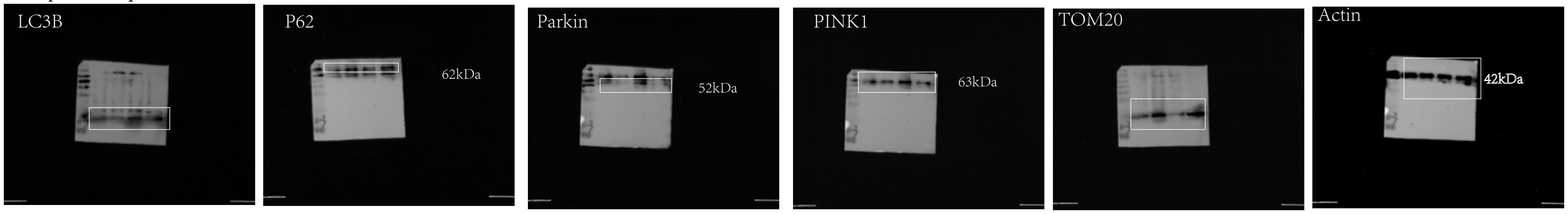

Figure 4N First Group 1

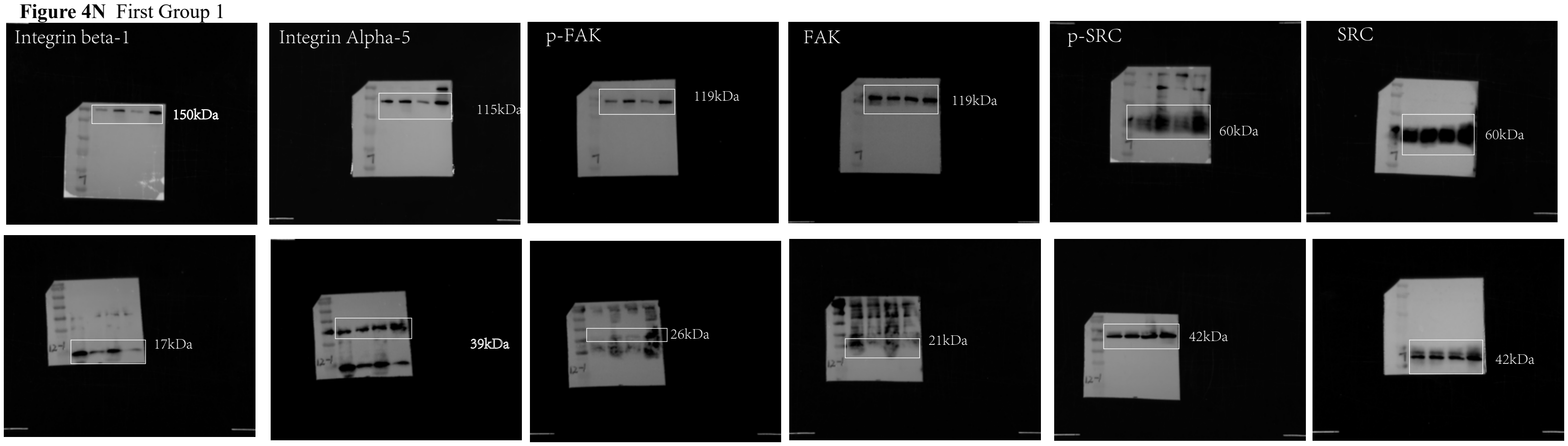

Repeat Group 2

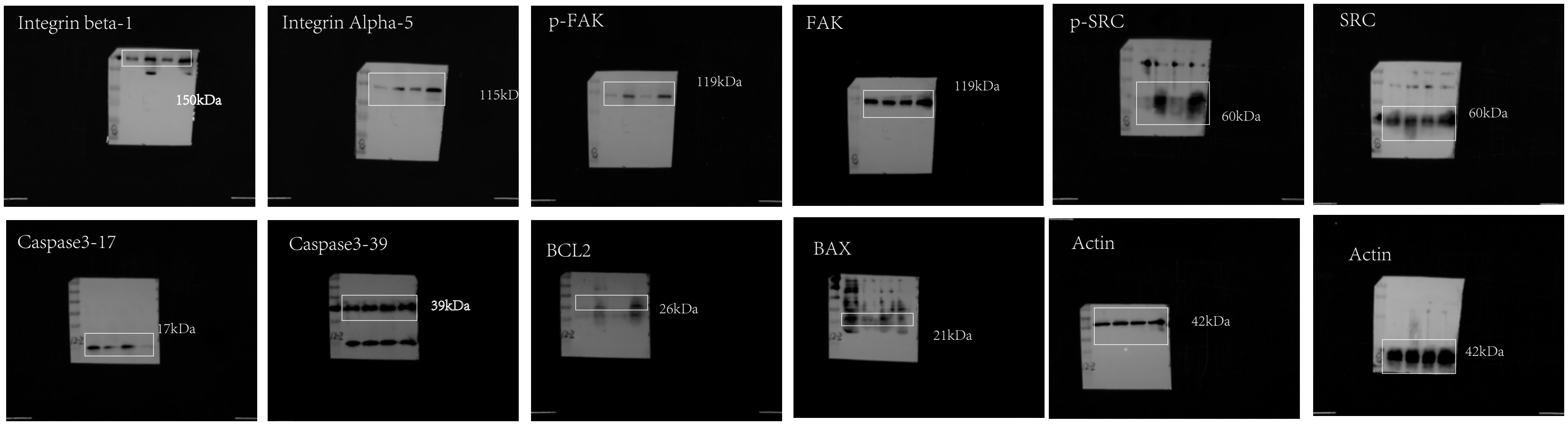

Repeat Group 3

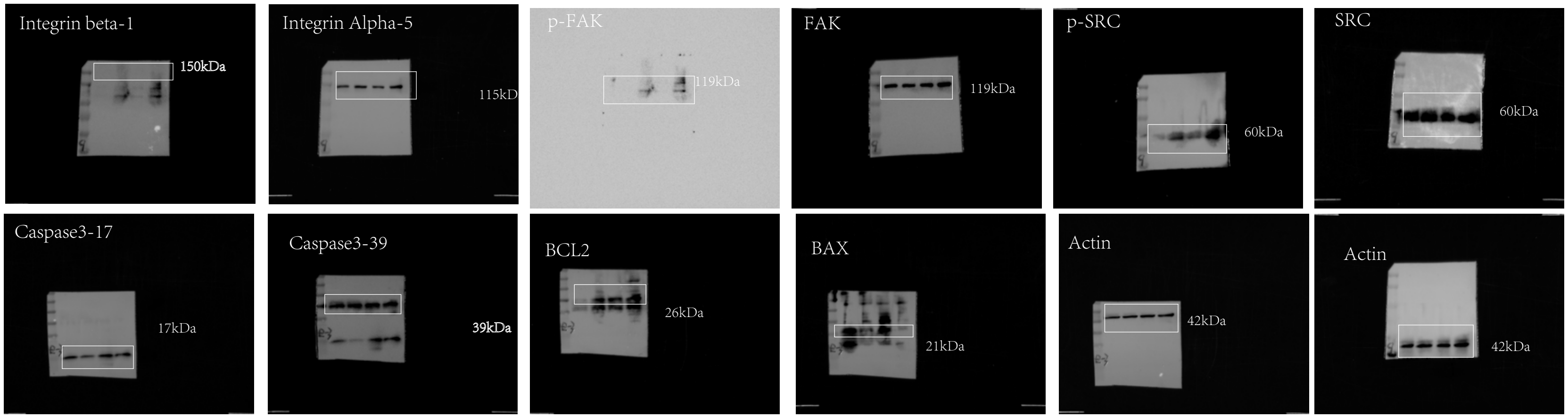

Figure 5C First Group 1 Repeat Group 2 Repeat Group 3

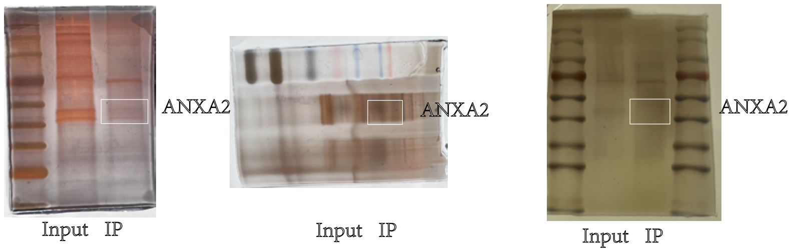

Figrue 5E First Group 1： NET-DNA pull-down Repeat Group 2 Repeat Group 3

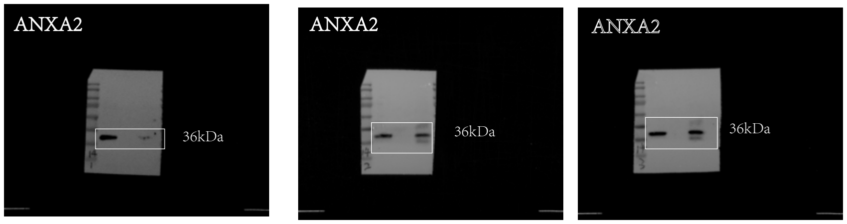

Figure 5G First Group 1： NET-DNA and ANXA2 Repeat Group 2 Repeat Group 3

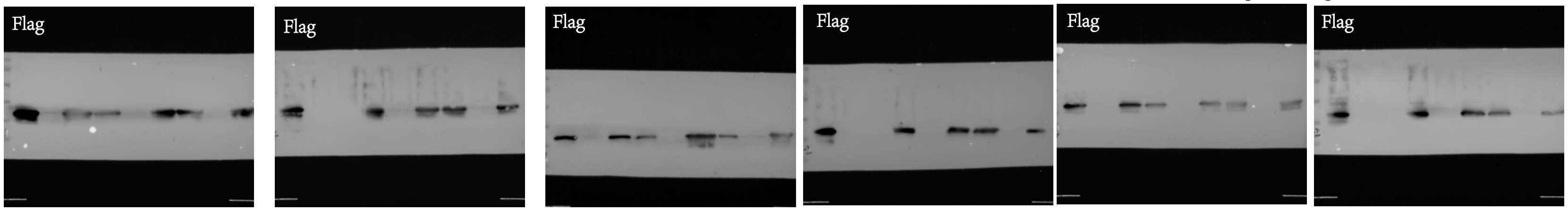

Figure 6A First Group 1

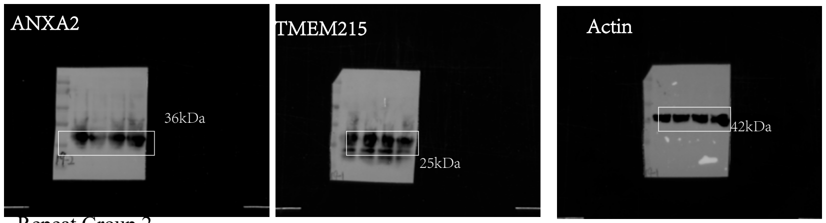

Repeat Group 2

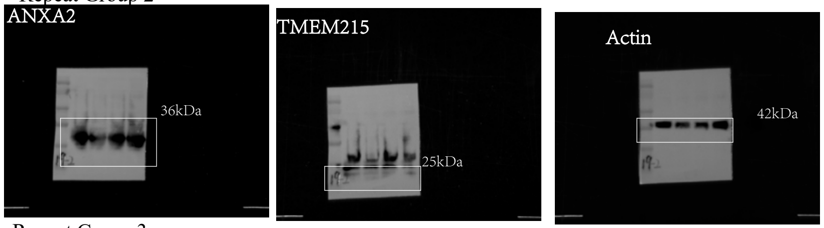

Repeat Group 3

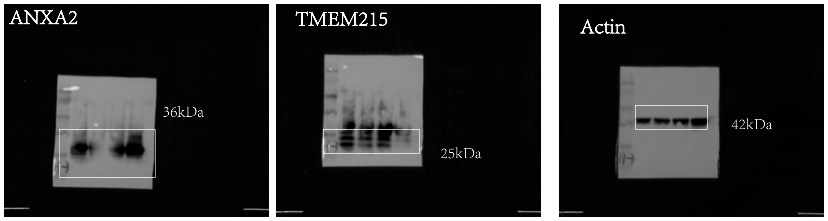

Figrue 6B/C First Group 1 Repeat Group 2 Repeat Group 3

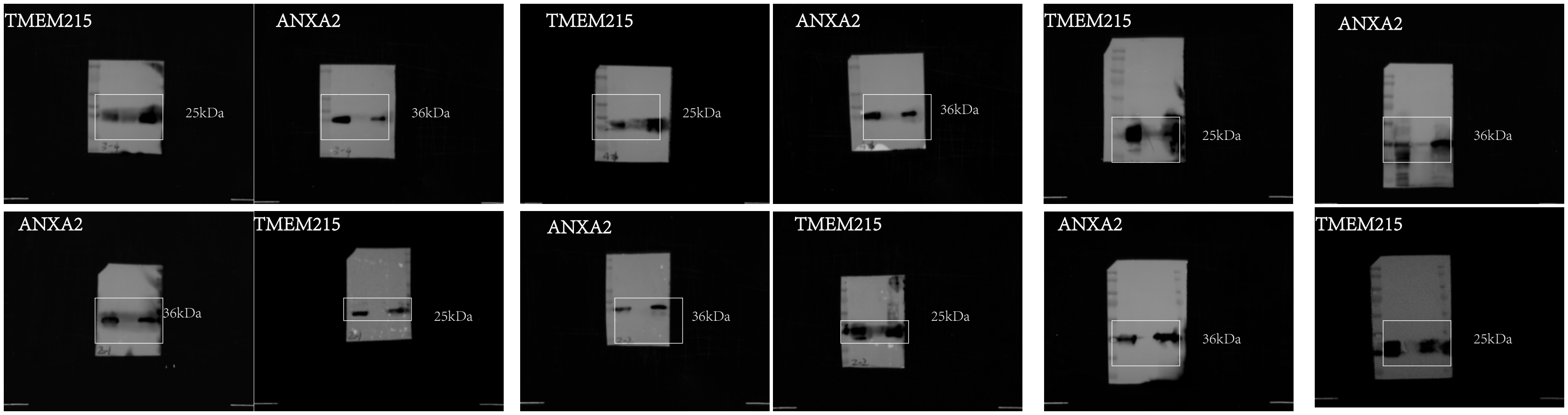

Figure 6F First Group 1

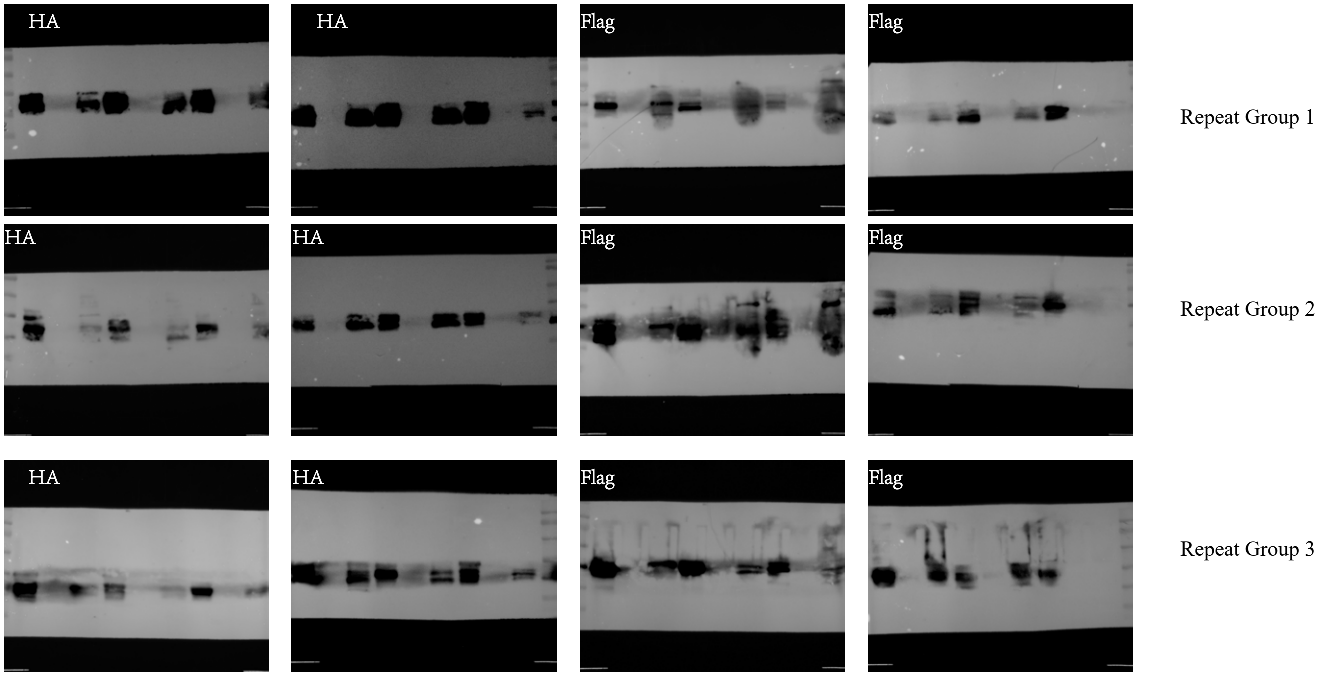

Figure 6I First Group 1

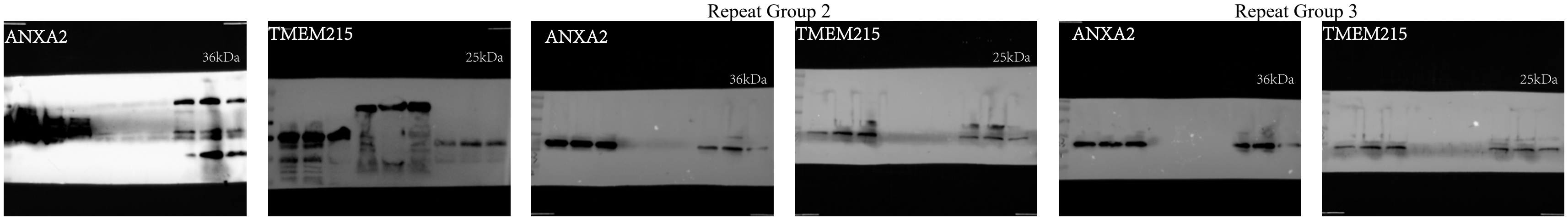

Figure 6L First Group 1

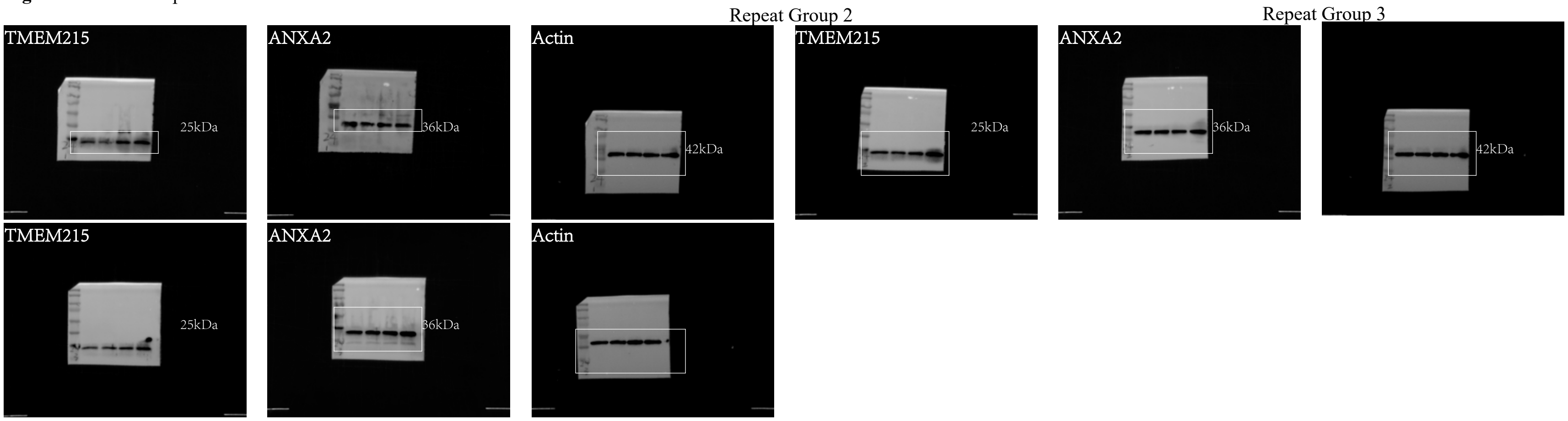

Figrue 7B First Group 1： NET-DNA+si-NC、NET-DNA+si-ANXA2、NET-DNA+si-TMEM215、CON520+si-NC、OE-ANXA2+si-NC、OE-ANXA2+si-TMEM215

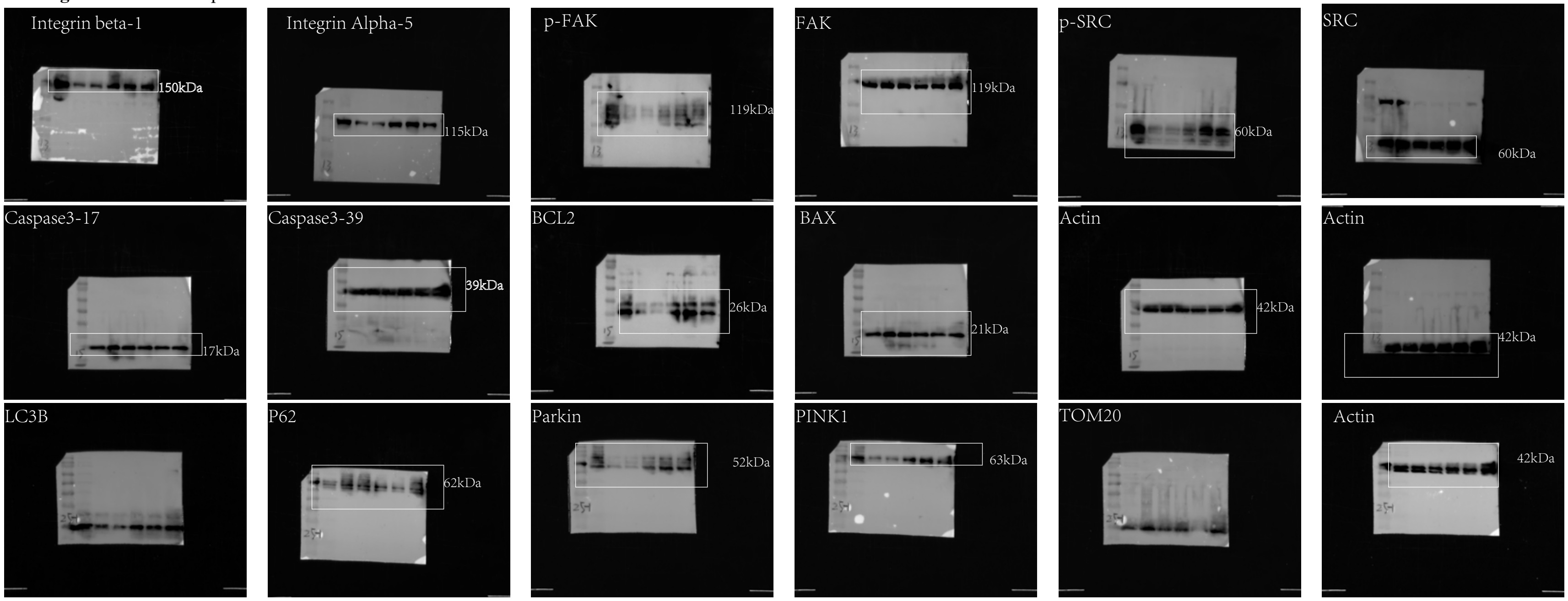

Repeat Group 2

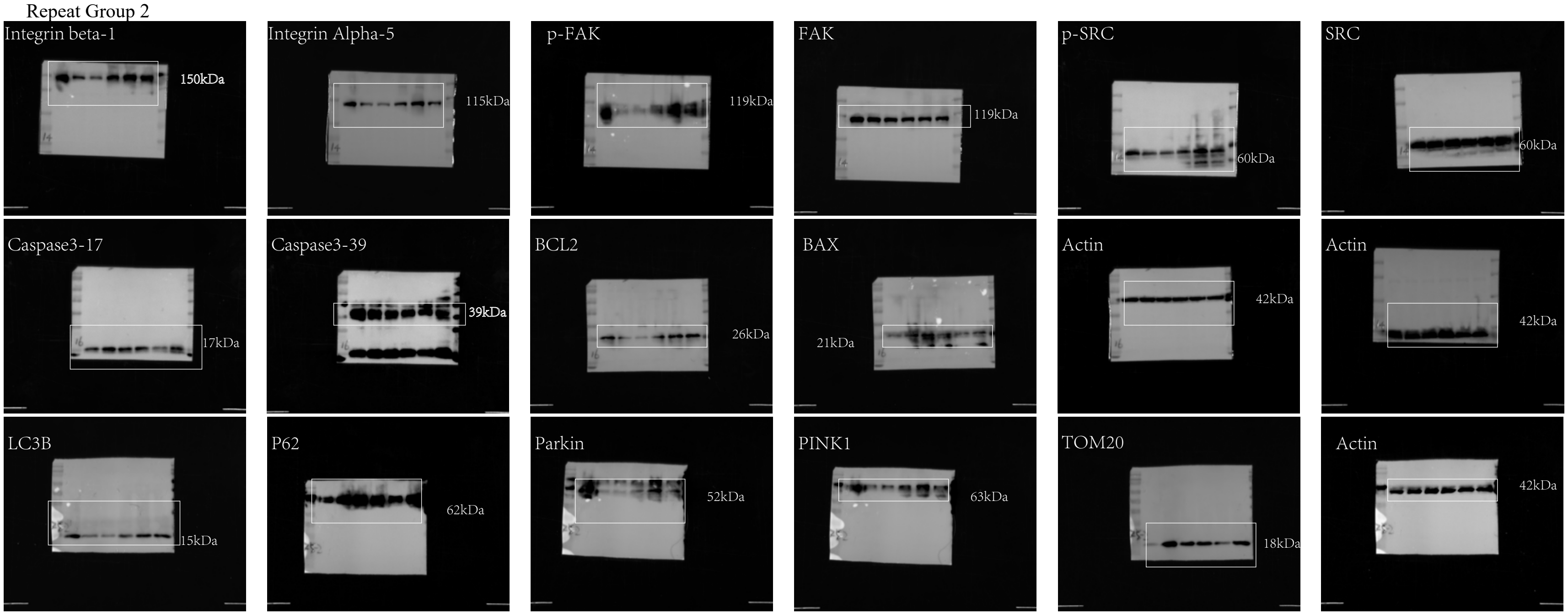

Repeat Group 3

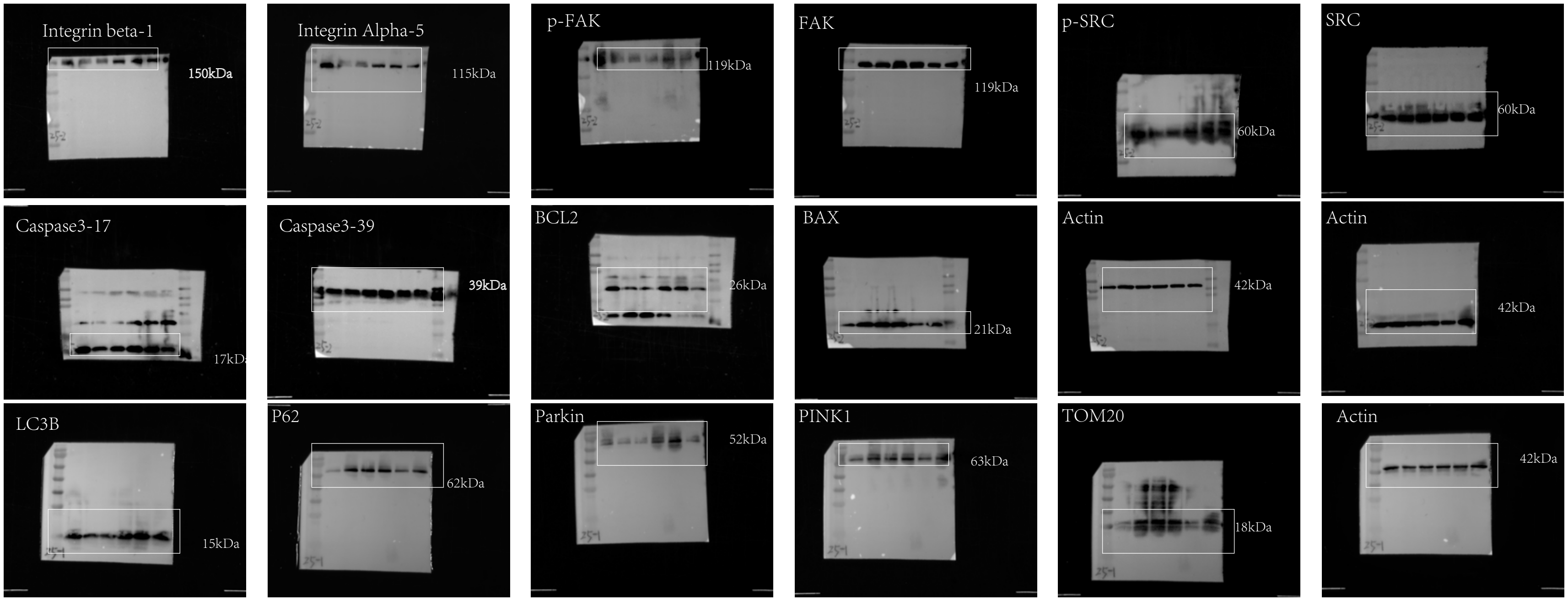

Figrue 7H First Group 1

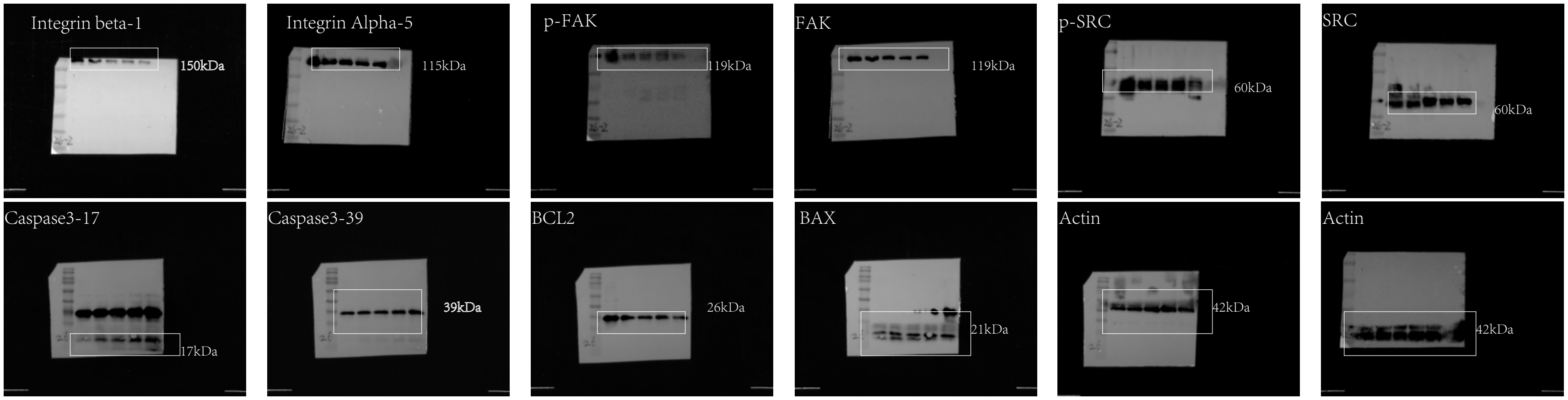

Repeat Group 2

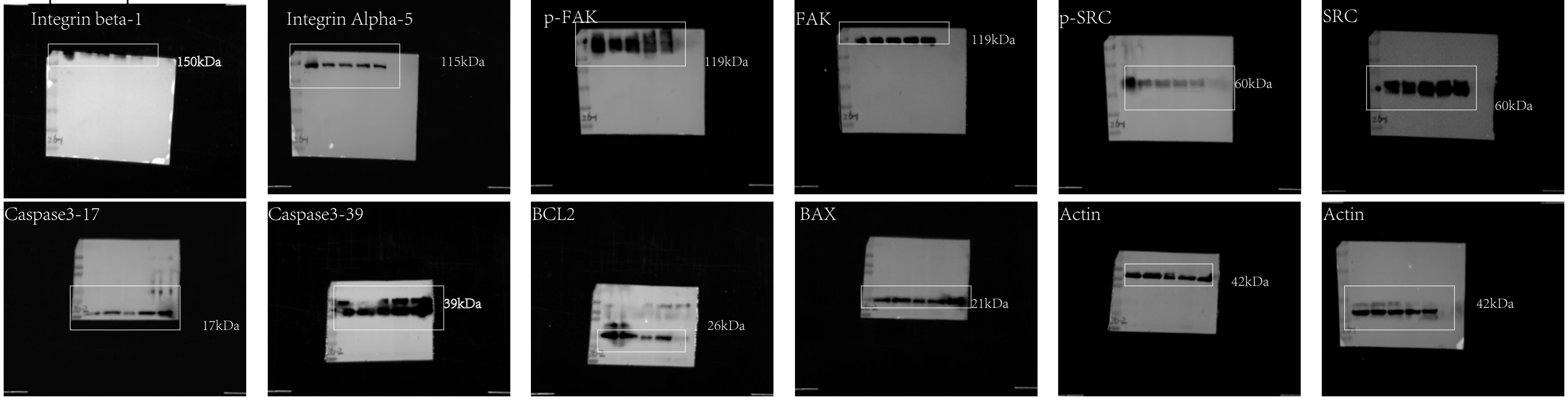

Repeat Group 3

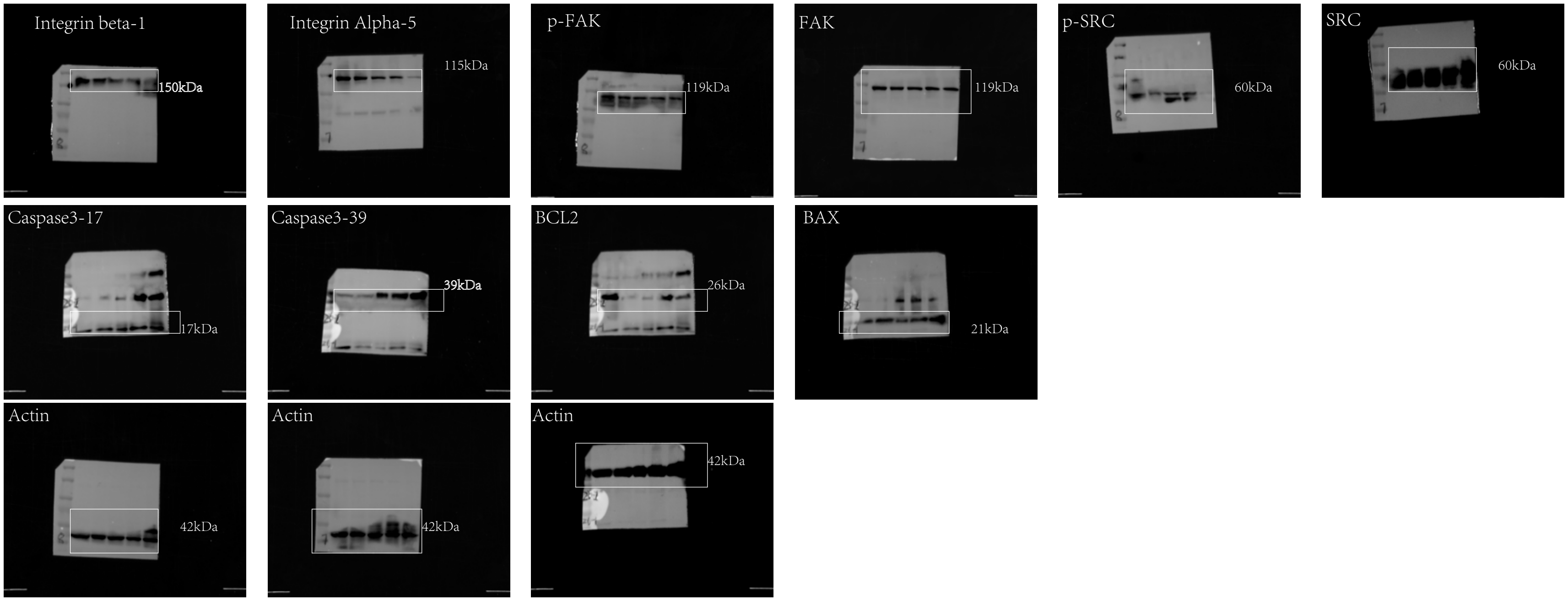

Figure 8B First Group 1

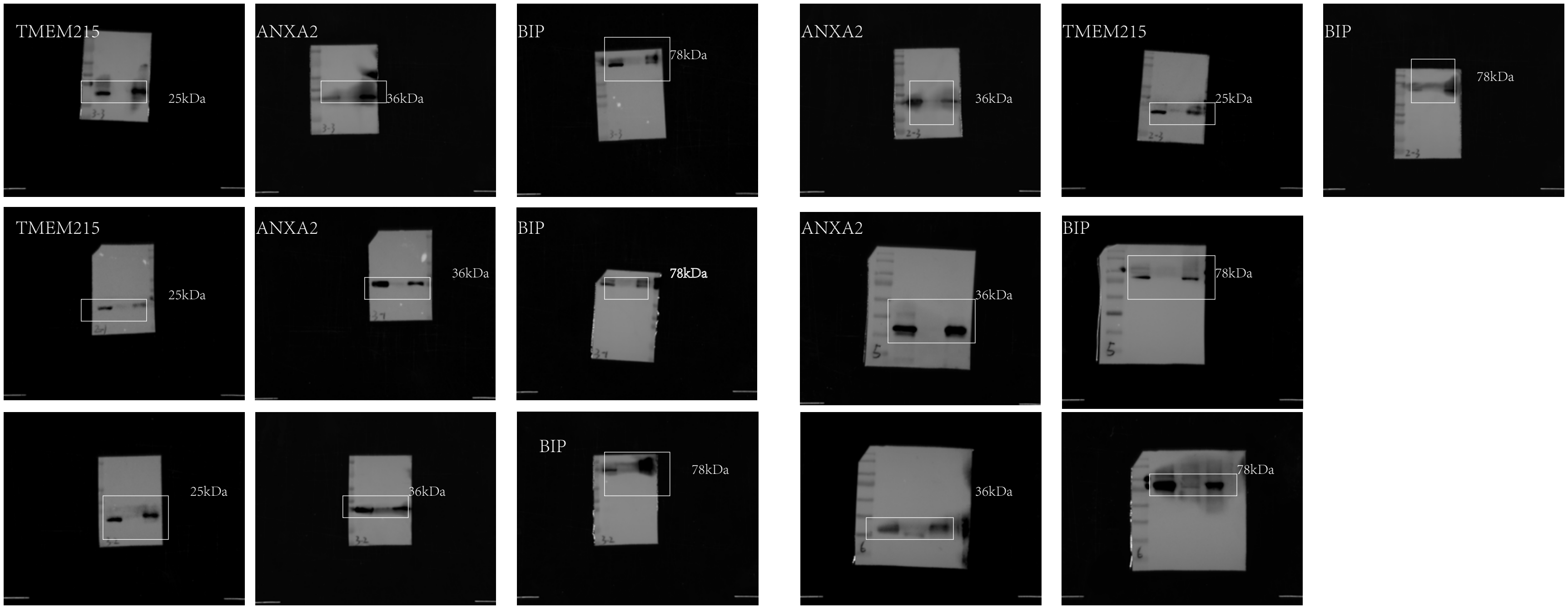

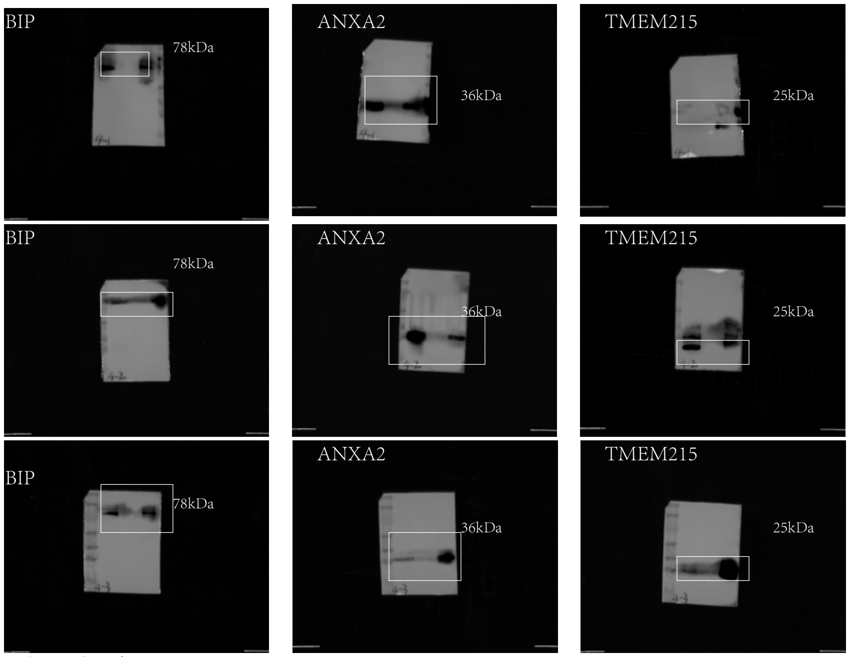

Figure 8F First Group 1 Repeat Group 2 Repeat Group3

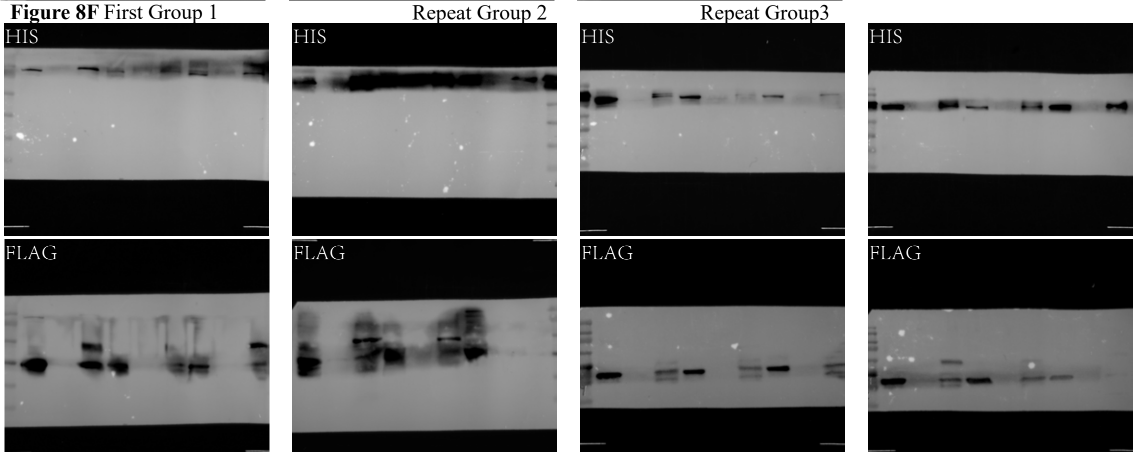

Figure 8N First Group 1 ： CON、OE-TMEM215、OE-TMEM215+si-BIP、OE-TMEM215+si-ANXA2、si-ANXA2+OE-BIP、OE-ANXA2+si-BIP

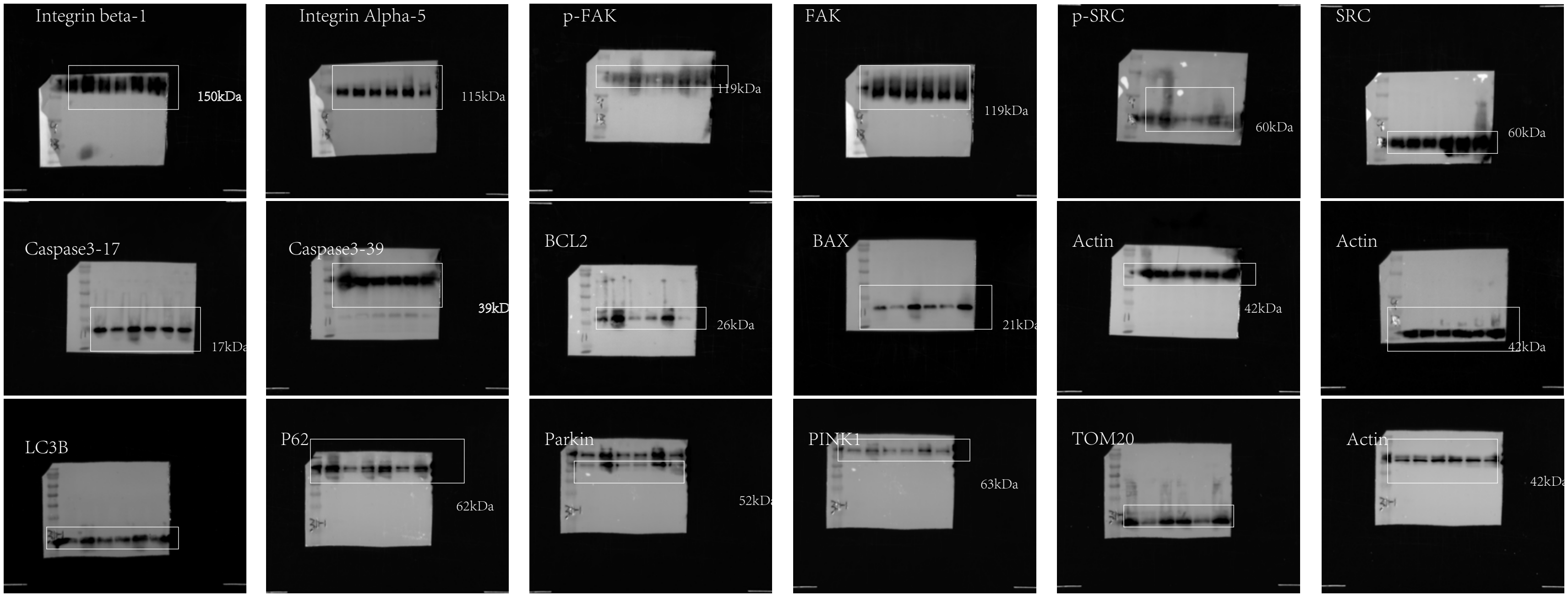

Repeat Group 2

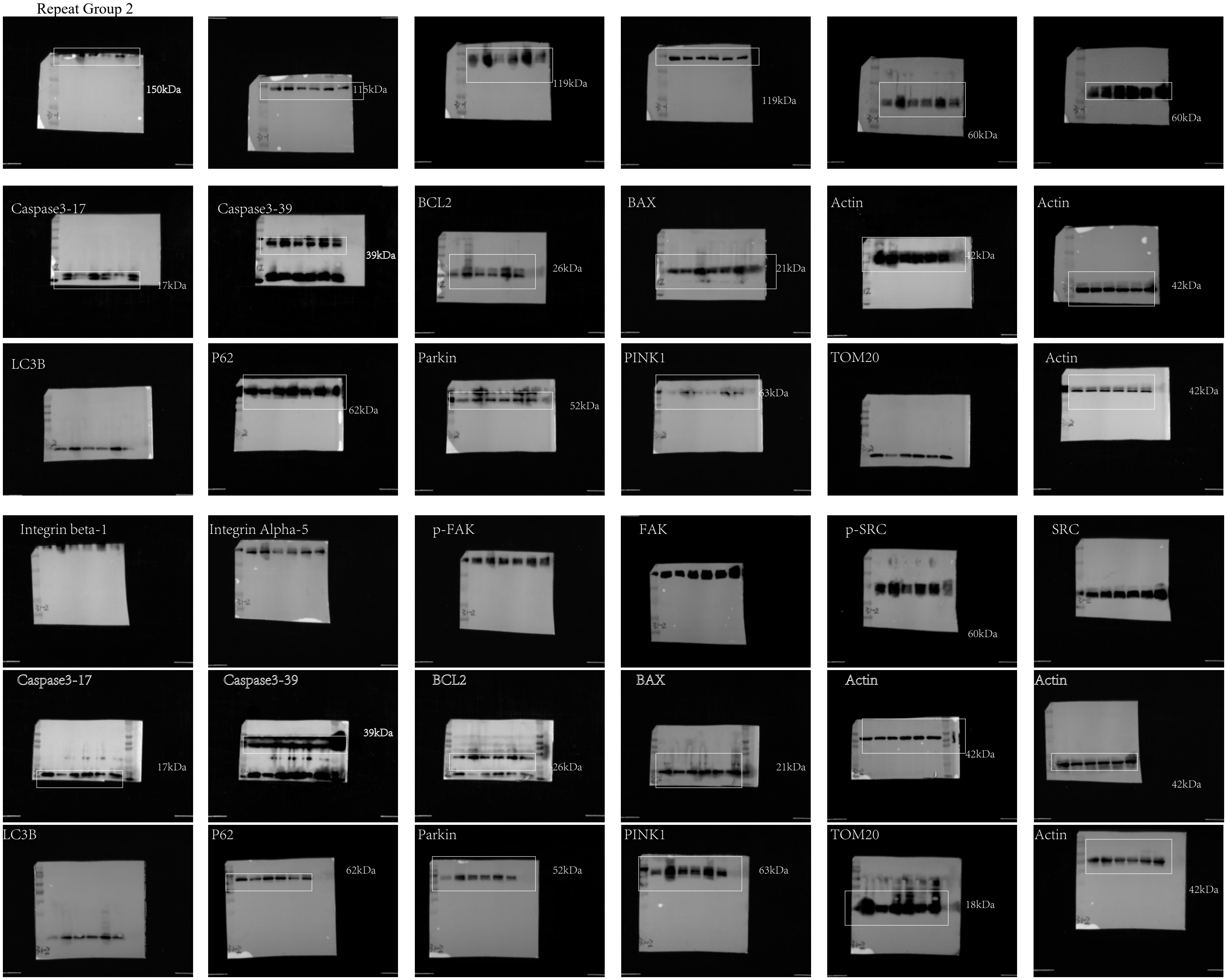

Figure 8O First Group 1

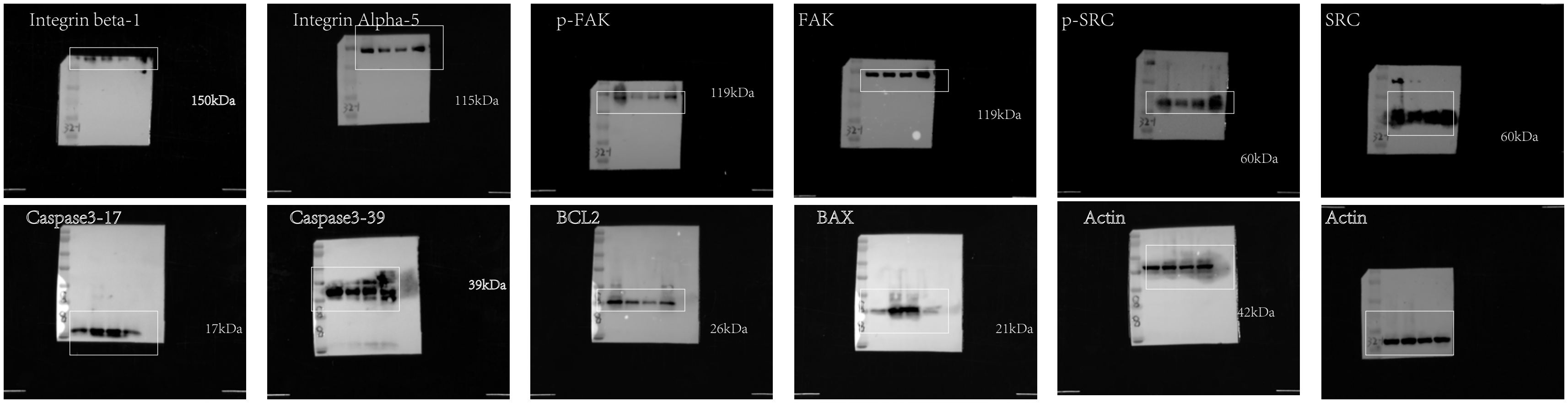

Repeat Group 2

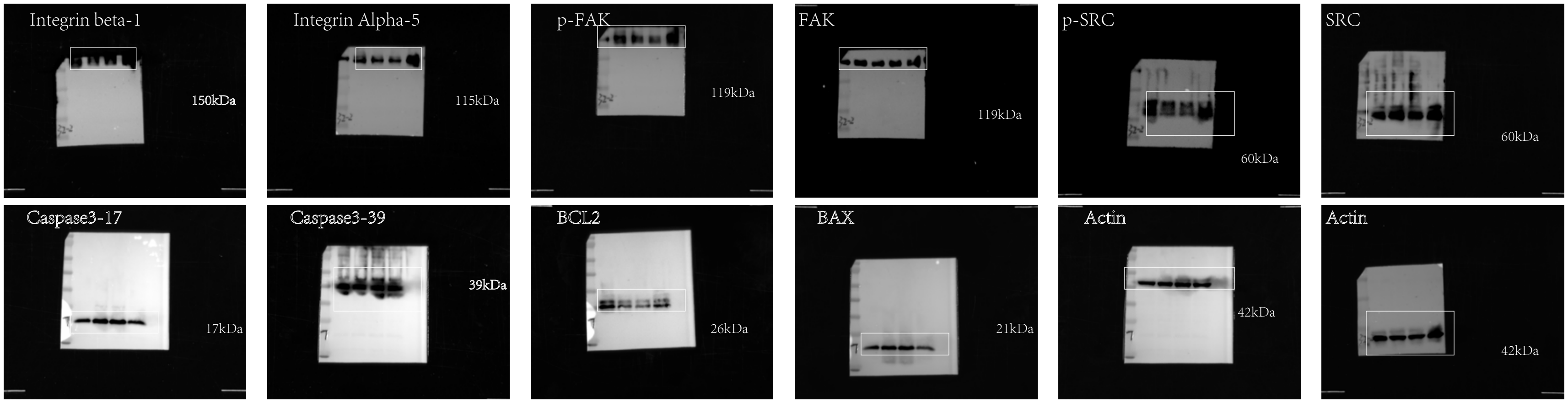

Repeat Group 3

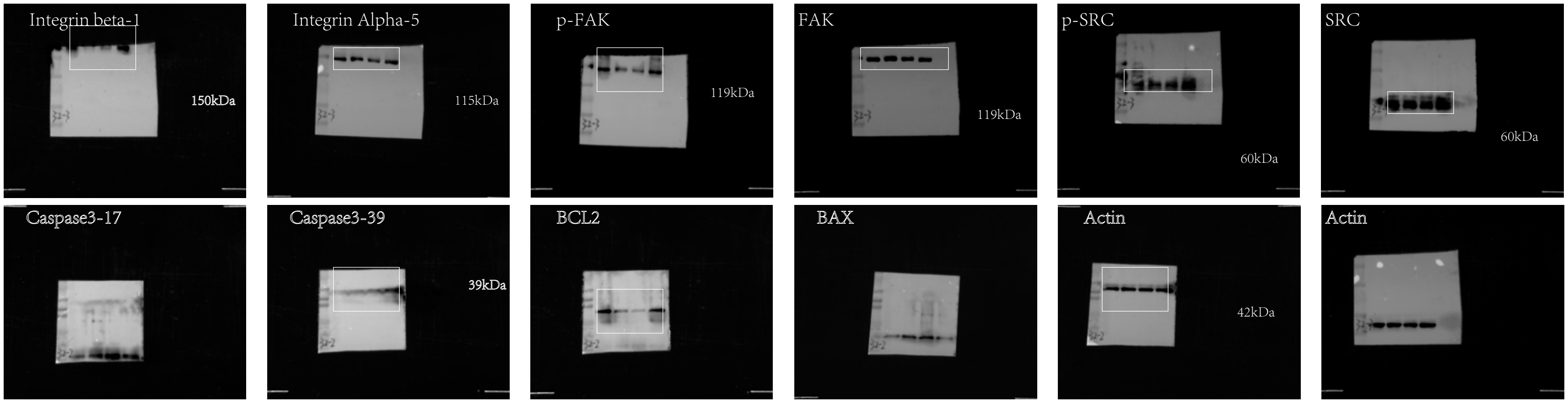

Figure 8P First Group 1

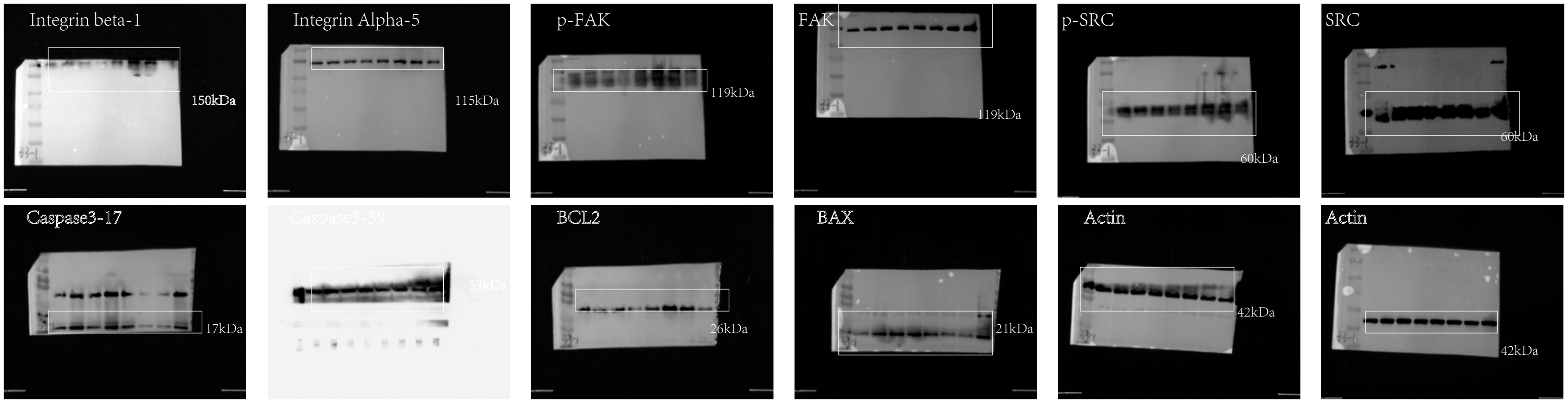

Repeat Group 2

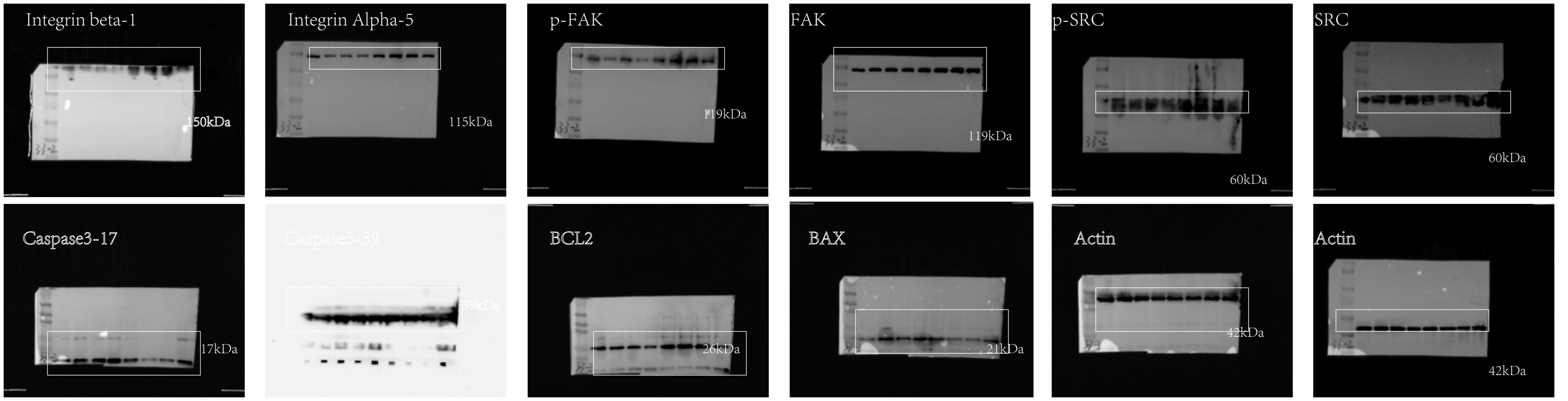

Repeat Group 3

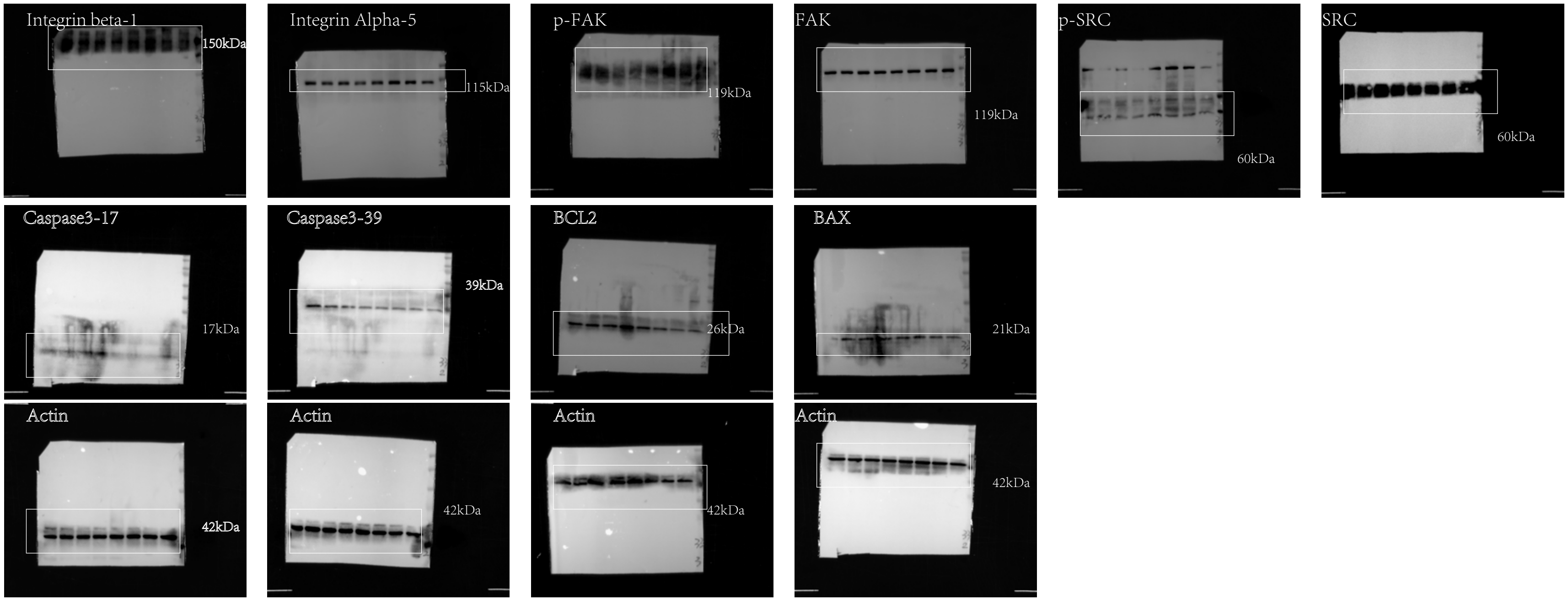

Figure S1A First Group 1：EN#1 EN#2 EN#3 EN#4 EC#1 EC#2 EC#3 EC#4

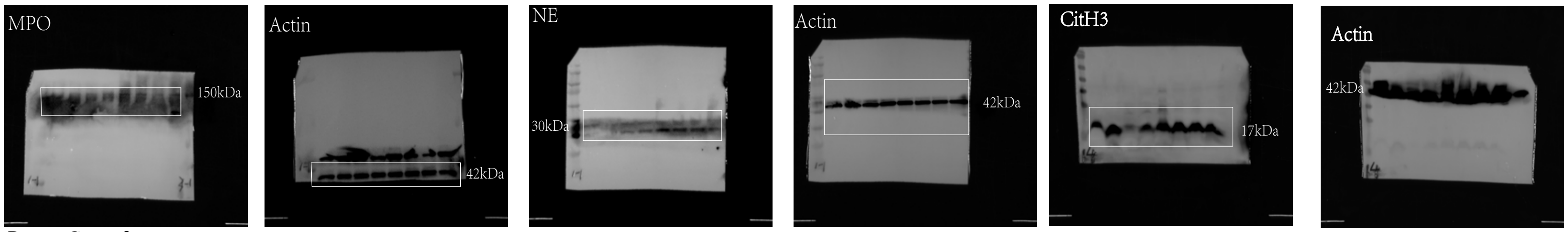

Repeat Group 2

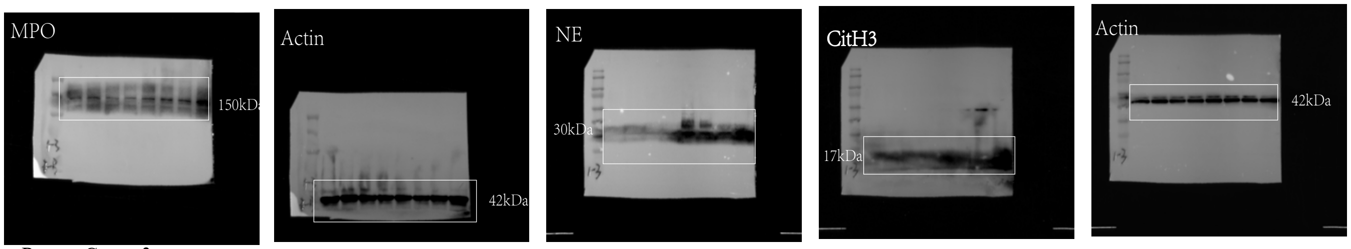

Repeat Group 3

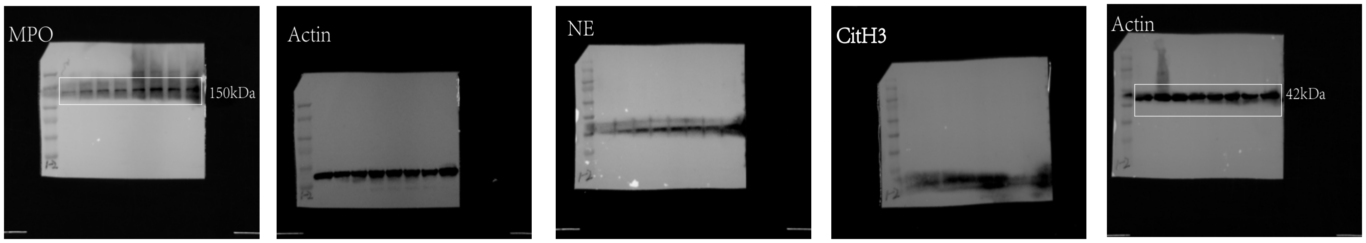

Figure S First Group 1 分组：salt\、LPS(10ug/mouse)、LPS+DNaseI(100U/mouse)

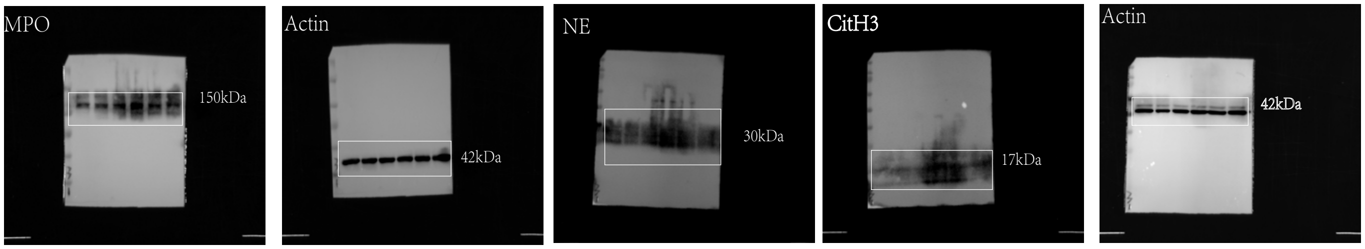

Repeat Group 2

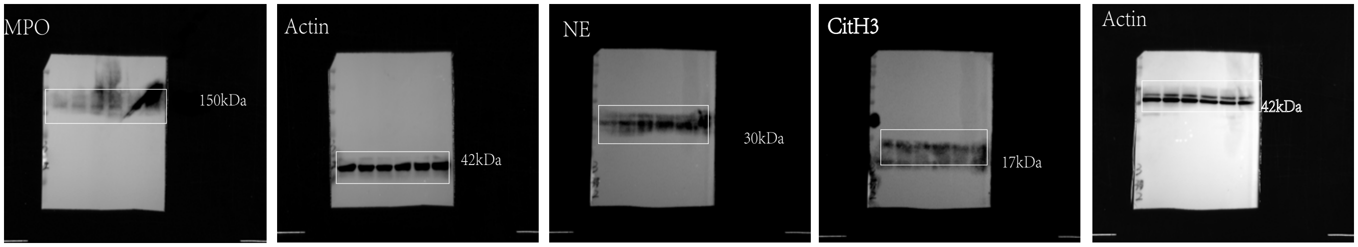

Repeat Group 3

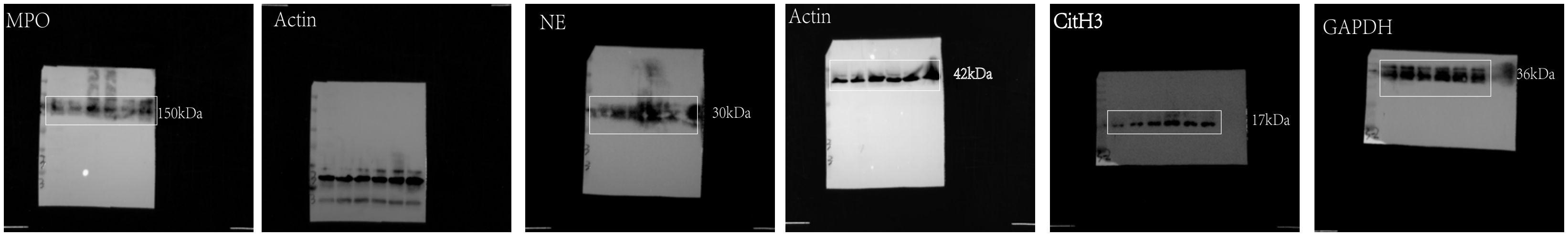

Figure S1 K First Group 1

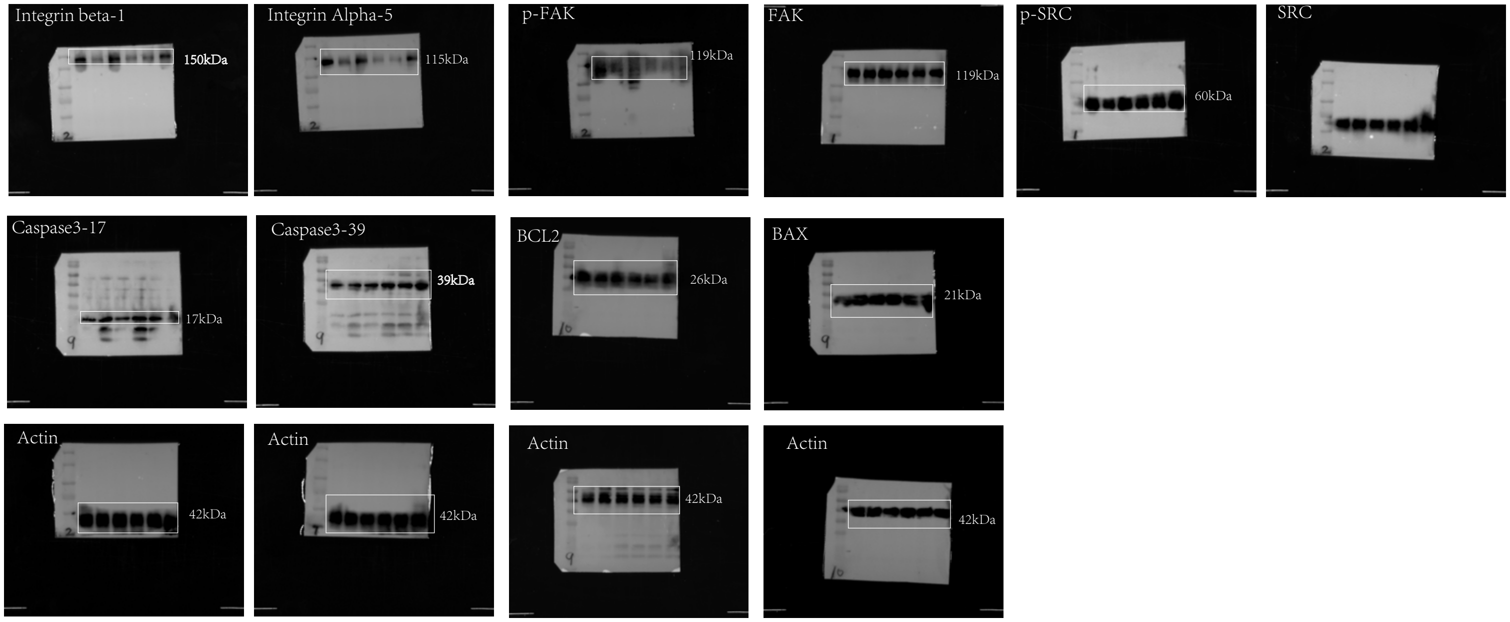

Repeat Group 2

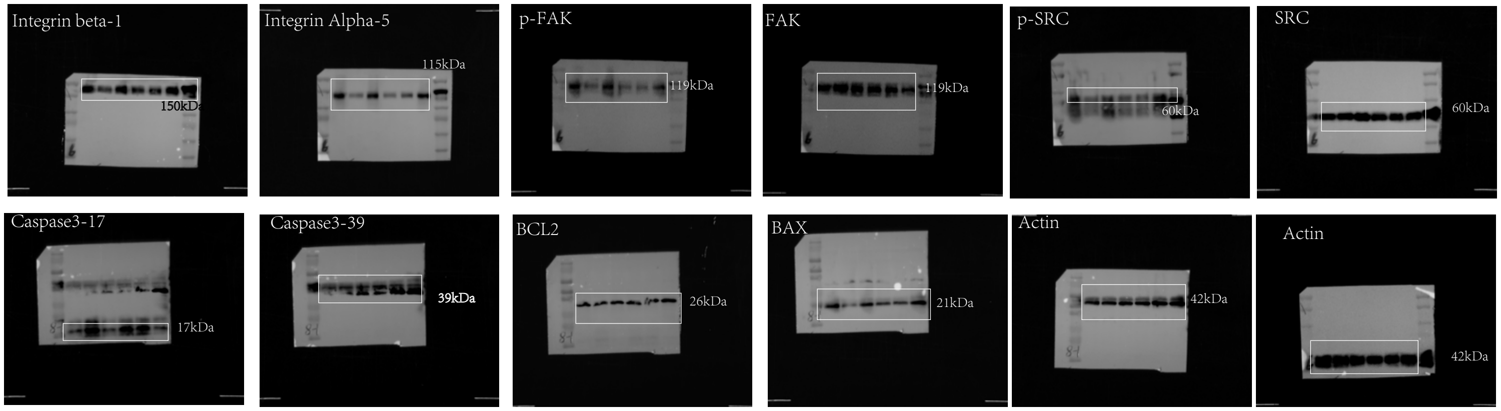

Repeat Group 3

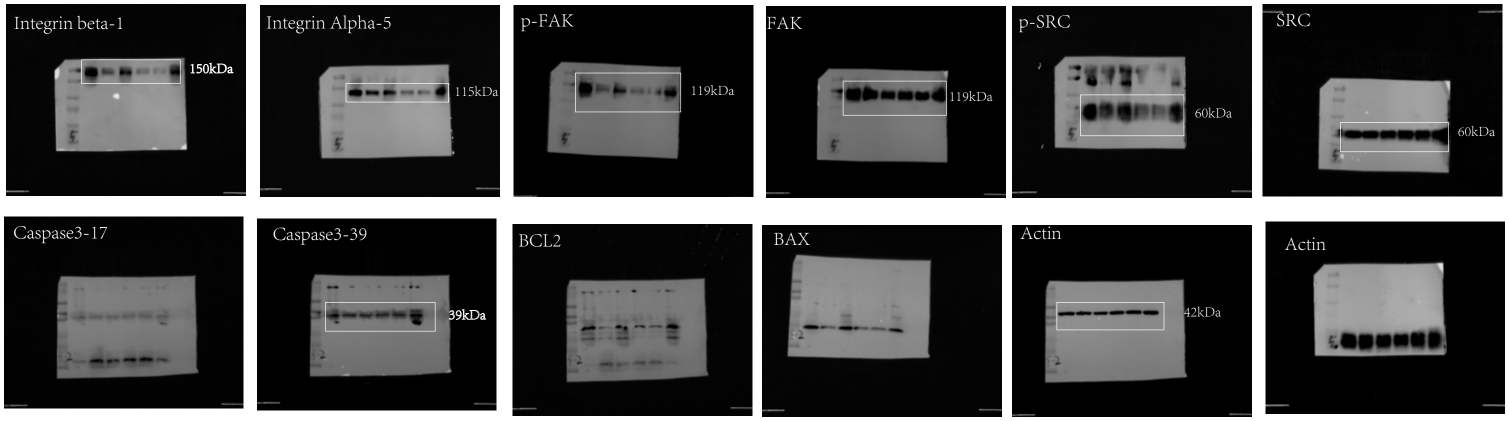

Figure S2A First Group 1 Repeat Group 2 Repeat Group 3

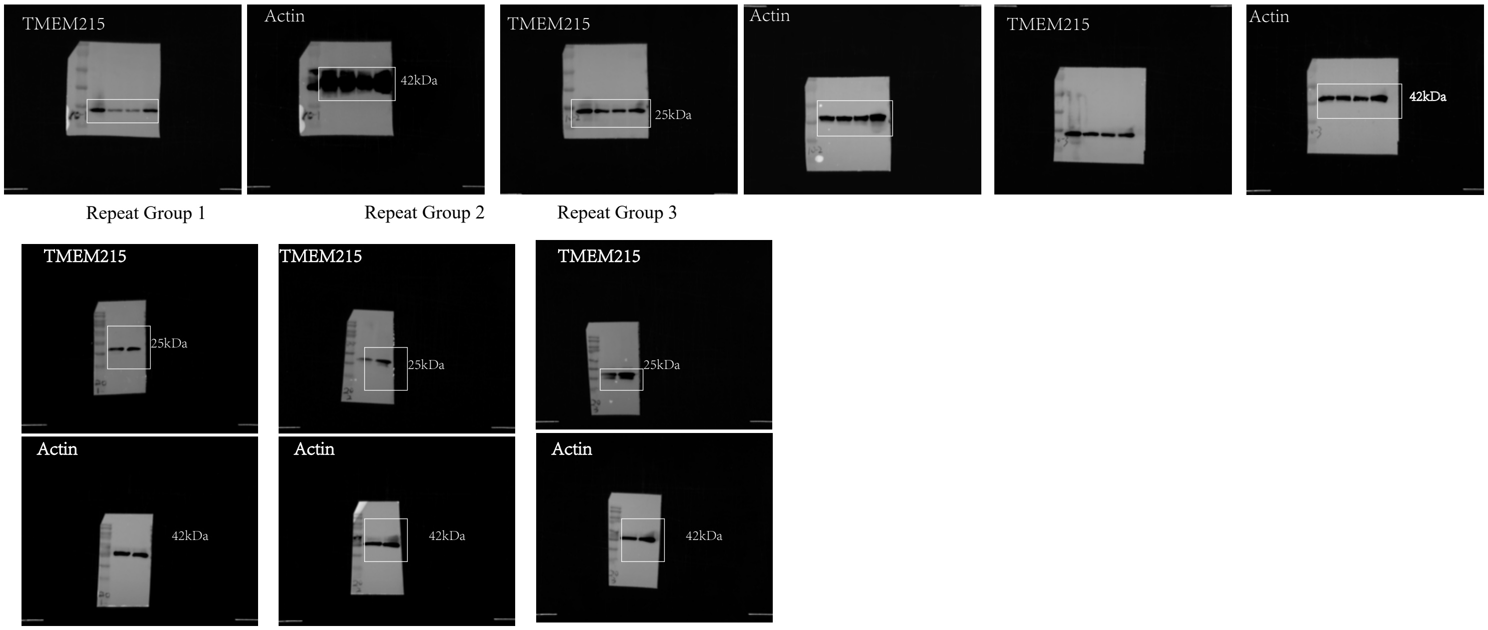

Figrue S2B First Group 1 Repeat Group 2 Repeat Group 3

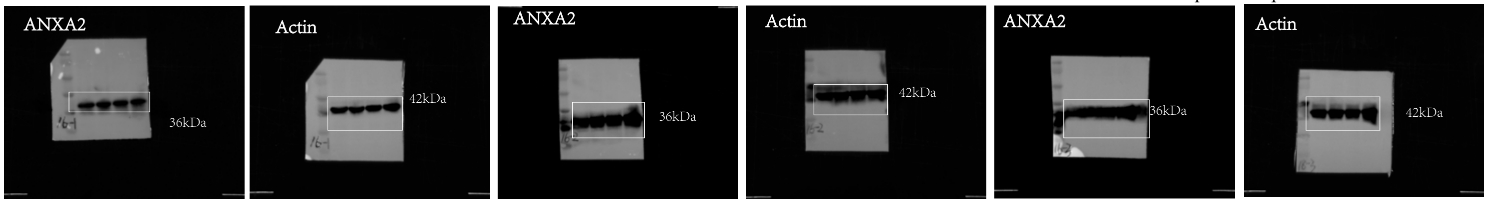

Figure S2C First Group 1 Repeat Group 2 Repeat Group 3

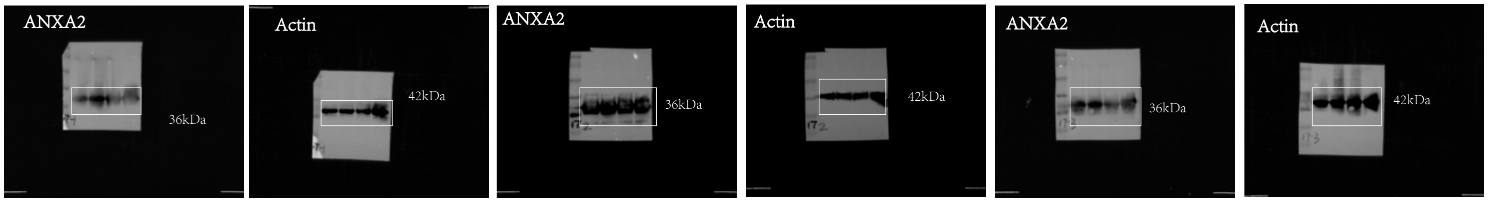

Figure S2D First Group 1 Repeat Group 2 Repeat Group 3

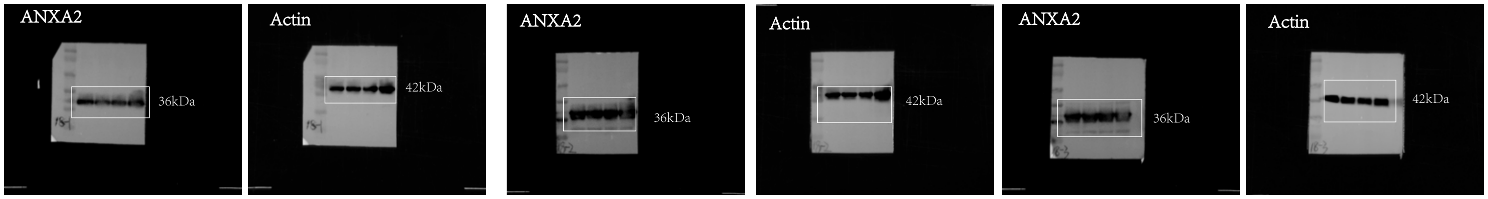

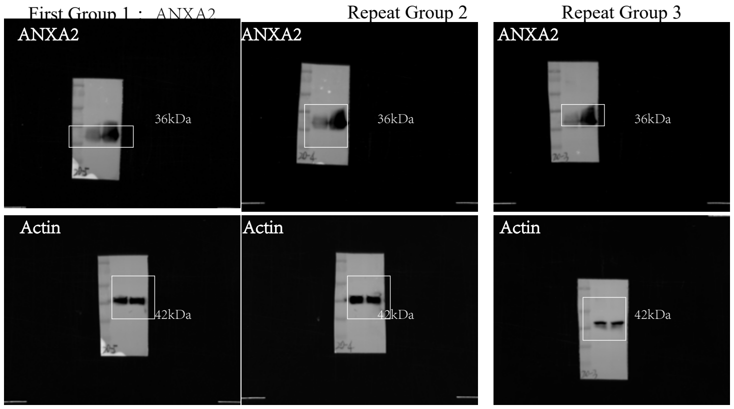

Figrue S2E First Group 1

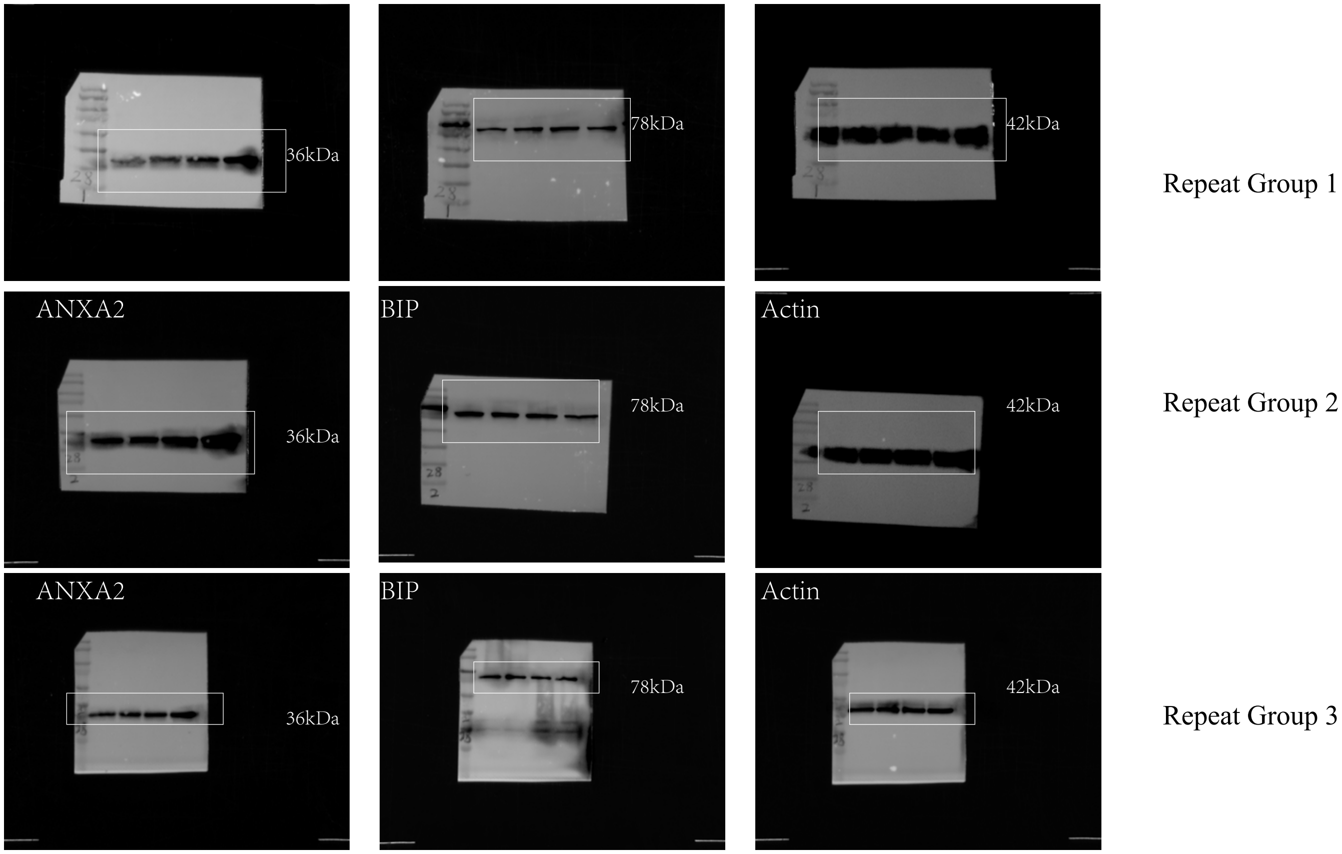

Figure S2F First Group 1

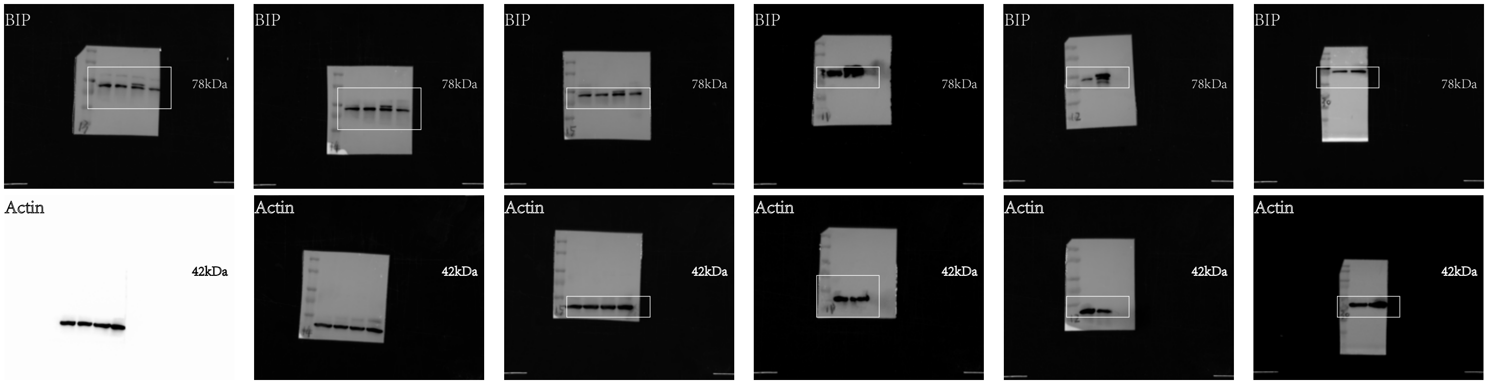

Supplement: Supplementary file 2 — Supporting File 2: advs75442‐sup‐0002‐Data.pdf. [file ADVS-13-e75442-s003.pdf]
