# Supplementary material for: NET‐DNA Activates the ANXA2/TMEM215/BiP Axis to Promote Mitophagy‐Mediated Anoikis Resistance in Endometriosis
Source: Adv Sci (Weinh). 2026 Apr 27;13(40):e75442. doi: 10.1002/advs.75442 (PMC13335634; doi:10.1002/advs.75442)
Supplement: Supplementary file 3 — Supporting File 3: advs75442‐sup‐0003‐TableS1.docx. [file ADVS-13-e75442-s002.docx]

**Table S1. List of animals, antibodies, siRNA/shRNA, plasmids, and primers used in the study**

***This table lists the animals, antibodies, siRNA/shRNA, plasmids, and primers used in the experiments, including their types, catalog numbers, sources, and relevant conditions.***

***Animals(in vivo studies)***

| **species** | **Vendor or source** | **Background Strain** | **sex** | **Persistent ID / URL** |
| --- | --- | --- | --- | --- |
| Wild-type C57BL/6J | Animal Experiment Center, The Second Affiliated Hospital of Harbin Medical University | C57BL/6J | Female | None |

***Primary Antibodies***

| **Target antigen** | **Full name** | **Manufacturer** | **Catalog number** | **Dilution ratio** | **Notes** |
| --- | --- | --- | --- | --- | --- |
| Vimentin | Vimentin Monoclonal Antibody | Proteintech | 60330-1-Ig | IF 1:100 | Mouse monoclonal antibody; detects human/mouse/rat Vimentin; validated for WB, IHC, IF, IP |
| E-cadherin | E-cadherin Monoclonal Antibody | Proteintech | 60902-1-Ig | IF 1:100 | Mouse monoclonal antibody; detects human E-cadherin; validated for WB, IHC, IF, ELISA |
| Caspase-3 | Caspase-3 Antibody | CST | #9662 | WB 1:1000 | Rabbit monoclonal antibody; detects human/mouse/rat; validated for WB, IHC, IP |
| BCL2 | BCL2 Polyclonal antibody | Proteintech | 12789-1-AP | WB 1:9000 | Rabbit polyclonal antibody; detects human BCL2; validated for WB, IHC, IF, IP |
| BAX | BAX Polyclonal antibody | Proteintech | 50599-2-Ig | WB 1:10000 | Rabbit monoclonal antibody; detects human/mouse/rat; validated for WB, IHC, IP |
| LC3B | LC3B Polyclonal antibody | Proteintech | 18725-1-AP | WB 1:1500 | Rabbit polyclonal antibody; detects human/mouse/rat; validated for WB, IHC, IF, ELISA |
| LC3B | LC3B (E5Q2K) Mouse Monoclonal Antibody | CST | #83506 | WB 1:1500  IF 1:50  IHC 1:200 | Mouse monoclonal antibody; detects human/mouse/rat; validated for WB, IHC, IF |
| P62 | P62/SQSTM1 Polyclonal antibody | Proteintech | 18420-1-AP | WB 1:10000 | Rabbit polyclonal antibody; human reactive; validated for WB, IHC, IF, IP |
| PARKIN | Parkin (PARK2) Polyclonal antibody | Proteintech | 14060-1-AP | WB 1:4000  IHC 1:500 | Rabbit polyclonal antibody; detects human/mouse/rat/pig; validated for WB |
| PINK1 | PINK1 Polyclonal antibody | Proteintech | 23274-1-AP | WB 1:2000  IHC 1:500 | Rabbit polyclonal antibody; detects human/mouse/rat; validated for WB |
| TOM20 | Tom20 (D8T4N) Rabbit mAb | CST | #42406 | WB 1:1000  IF 1:100 | Rabbit monoclonal antibody; detects endogenous human/mouse/rat TOM20 |
| Calnexin | Calnexin Polyclonal antibody | Proteintech | 10427-2-AP | IF 1:100 | Rabbit polyclonal antibody; detects Human/Mouse/Rat; validated for WB, IHC, IF/ICC, FC, ELISA |
| ANXA2 | Annexin A2 Monoclonal Antibody (Clone 3H1B11) | Proteintech | 60051-1-Ig | WB 1:10000  IF 1:500  IP 1:50 | Mouse monoclonal antibody; detects human/mouse/rat ANXA2; validated for WB, IHC, IF, IP; https://www.ptgcn.com/products/ANXA2-Antibody-60051-1-Ig.htm |
| BiP (GRP78) | GRP78/BiP Polyclonal antibody | Proteintech | 11587-1-AP | WB 1:6000  IF 1:500  IP 1:100 | Rabbit polyclonal antibody; detects human/mouse/rat; validated for WB, IHC |
| BiP (GRP78) | GRP78 BiP Mouse mAb | ZenBio | 222310 | IF 1:100 | Mouse monoclonal antibody; detects Human/Mouse/Rat/Monkey/Rabbit; validated for WB, IHC-P, ICC/IF, FC |
| TMEM215 | TMEM215 Polyclonal antibody | Novus Biologicals | NBP2-85945 | WB 1:500  IHC 1:100  IF 1:100  IP 1:50 | Rabbit polyclonal antibody; detects human/mouse TMEM215; validated for WB; https://www.novusbio.com/products/tmem215-antibody_nbp2-85945 |
| Integrin β1 | Integrin beta-1 (ITGB1) Antibody | UpingBio | YP-mAb-17031 | WB 1:2000  IHC 1:50 | Mouse monoclonal antibody; detects human/mouse/rat |
| Integrin α5 | Integrin alpha-5 (ITGA5) Antibody | UpingBio | YP-Ab-16842 | WB 1:2000  IHC 1:200 | Mouse monoclonal antibody; detects human/mouse/rat |
| FAK | Focal adhesion kinase 1 (PTK2) | UpingBio | YP-Ab-14142 | WB 1:2000 | Mouse monoclonal antibody; detects human FAK |
| p-FAK (Ser732) | Phospho-FAK (Ser732) Antibody | UpingBio | YP-Ab-10343 | WB 1:2000  IHC 1:50 | Rabbit polyclonal antibody; detects phosphorylated FAK (Ser732) |
| p-Src (Ser75) | Phospho-Src (Ser75) Antibody | UpingBio | YP-Ab-14501 | WB 1:2000  IHC 1:50 | Rabbit polyclonal antibody; detects phosphorylated Src (Ser75) |
| c-Src | c-Src mouse mAb (PTR2316) | UpingBio | YP-Ab-14775 | WB 1:2000 | Mouse monoclonal antibody; detects endogenous human/mouse/rat c-Src |
| MPO | Myeloperoxidase Monoclonal Antibody (Clone 4C11F6) | Proteintech | 66177-1-Ig | WB 1:1000  IF 1:500  IHC 1:100 | Mouse monoclonal antibody; detects human/rat MPO; validated for WB, IHC, IF, ELISA |
| NE | Neutrophil Elastase Antibody | Proteintech | 27642-1-Ap | WB 1:1000 | Rabbit polyclonal antibody; detects NE |
| CitH3 | Citrullinated Histone H3 Antibody | Abcam | ab5103 | WB 1:5000  IF 1:1000  IHC 1:100 | Rabbit polyclonal antibody; detects CitH3 |
| IP3R-I | IP3R-I (E-8) Mouse Monoclonal Antibody | Santa Cruz | sc-271197 | IF 1:50 | Mouse monoclonal antibody (IgG1); detects human/mouse/rat; validated for WB, IHC(P), IF, ELISA |
| GPR75 | GPR75 Polyclonal Antibody | Proteintech | 17972-1-AP | IF 1:100 | Rabbit polyclonal antibody; detects Human/Mouse (literature also reports Rat); validated for IHC, IF/ICC, IF-P, ELISA |
| FLAG | DYKDDDDK Tag Antibody | CST | #14793 | WB 1:1000  IF 1:500  IP 1:50 | Rabbit monoclonal antibody; detects FLAG-tagged proteins |
| HA | HA-Tag Antibody | CST | #3724 | WB 1:1000  IF 1:800  IP 1:50 | Rabbit monoclonal antibody; detects HA-tag |
| His | His-Tag Antibody | Proteintech | 66005-1-Ig | WB 1:5000  IF 1:400  IP 1:50 | Mouse monoclonal antibody; detects 6×His-tagged proteins |
| GAPDH | GAPDH Antibody | Proteintech | 60004-1-Ig | WB 1:50000 | Mouse monoclonal antibody; loading control |
| β-Actin | Beta-Actin Antibody | Proteintech | 66009-1-Ig | WB 1:20000 | Mouse monoclonal antibody; loading control |
| Rabbit IgG isotype control | Rabbit IgG control Polyclonal Antibody | Proteintech | 30000-0-AP | IP 1:50 | Rabbit polyclonal control IgG; used as negative control for IP, WB, ELISA |
| Mouse IgG isotype control | Mouse IgG Control Antibody | Proteintech | B900620 | IP 1:50 | Mouse polyclonal control IgG; used as negative control for IP, WB |

***Secondary Antibodies***

| **Target antigen** | **Manufacturer** | **Catalog number** | **Dilution ratio** |
| --- | --- | --- | --- |
| Multi-rAb™ HRP-Goat Anti-Rabbit Recombinant Secondary Antibody (H+L) | Proteintech | RGAR001 | WB 1:5000 |
| Multi-rAb™ HRP-Goat Anti-Mouse Recombinant Secondary Antibody (H+L) | Proteintech | RGAM001 | WB 1:5000 |
| Rabbit IgG (Heavy Chain specific) | Proteintech | SA00001-7H | WB 1:5000 |
| Rabbit IgG (Light Chain) | Proteintech | SA00001-7L | WB 1:5000 |
| Multi-rAb® CoraLite® Plus 488-Goat Anti-Mouse Recombinant Secondary Antibody (H+L) | Proteintech | RGAM002 | IF：1:400 |
| Multi-rAb® CoraLite® Plus 488-Goat Anti-Rabbit Recombinant Secondary Antibody (H+L) | Proteintech | RGAR002 | IF：1:400 |
| Multi-rAb®  CoraLite® Plus  555-Goat  Anti-Mouse  Recombinant  Secondary  Antibody (H+L) | Proteintech | RGAM003 | IF: 1:400 |
| Multi-rAb®  CoraLite® Plus  555-Goat  Anti-Rabbit  Recombinant  Secondary  Antibody (H+L) | Proteintech | RGAR003 | IF: 1:400 |
| Rabbit Polymer Detection Kit (HRP-polymer anti-rabbit) | ZSGB-BIO | PV-6001 | IHC ready-to-use |
| Mouse Polymer Detection Kit (HRP-polymer anti-mouse) | ZSGB-BIO | PV-6002 | IHC ready-to-use |

***FACS analysis***

| **Antibody target** | **Clone** | **Fluorochrome** | **Manufacturer** | **Catalog No.** | **Application** |
| --- | --- | --- | --- | --- | --- |
| CD45 (mouse) | 30-F11 | PerCP-Cyanine5.5 | Elabscience®, China | E-AB-F1136J | FCM |
| CD3 (mouse) | 17A2 | FITC | Elabscience®, China | E-AB-F1013C | FCM |
| CD19 (mouse) | 1D3 | PE | Elabscience®, China | E-AB-F0986D | FCM |
| CD11c (mouse) | N418 | PE-Cyanine7 | Elabscience®, China | E-AB-F0991H | FCM |
| Ly6G (mouse) | 1A8 | APC | Elabscience®, China | E-AB-F1108E | FCM |
| F4/80 (mouse) | CI:A3-1 | PE-Cyanine7 | Elabscience®, China | E-AB-F0995H | FCM |
| CD11b (mouse) | M1/70 | APC | Elabscience®, China | E-AB-F1081E | FCM |
| CD161 (NK1.1, mouse) | PK136 | PE | Elabscience®, China | E-AB-F0987D | FCM |
| Viability (fixable) | — | eFluor 450 | eBioscience™ | 65-0863-14 | FCM |
| Fc receptor block (CD16/32) | 2.4G2 | — | Elabscience®, China | E-AB-F0997A | Blocking |
| Cell staining buffer | — | — | Elabscience®, China | E-AB-263039 | Buffer |

***siRNA/shRNA***

| **Description** | **sense** | **Vendor or source** |
| --- | --- | --- |
| NC | sense:UUCUCCGAACGUGUCACGUTT Anti-sense: ACGUGACACGUUCGGAGAATT | Hanbio |
| hs-TMEM215-si3 | sense:GACAGAUACUGUUGUUAUATT Anti-sense: UAUAACAACAGUAUCUGUCTT | Hanbio |
| hs-TMEM215-si2 | sense:CAGAACAGCCCGUAUGACAGA Anti-sense: UGUCAUACGGGCUGUUCUGCU | Hanbio |
| hs-TMEM215-si1 | sense:GUCUUUGGUUUCAUGUUCACC Anti-sense: UGAACAUGAAACCAAAGACGA | Hanbio |
| hs-HSPA5-si1 | sense:GGGCAAAGAUGUCAGGAAATT Anti-sense: UUUCCUGACAUCUUUGCCCTT | Hanbio |
| hs-HSPA5-si2 | sense:GAGUGACAGCUGAAGACAATT Anti-sense: UUGUCUUCAGCUGUCACUCTT | Hanbio |
| hs-HSPA5-si3 | sense:GAAUCAGAUUGGAGAUAAATT Anti-sense: UUUAUCUCCAAUCUGAUUCTT | Hanbio |
| st-h-ANXA2-si1 | CGGCTGTATGACTCCATGA | RIBOBIO |
| st-h-ANXA2-si2 | GACCAACCGCAGCAATGCA | RIBOBIO |
| st-h-ANXA2-si3 | GTCTGTCAAAGCCTATACT | RIBOBIO |
| pAAV-CAG-DIO-MasterRNAi155(mAnxa2)#1-mCherry-WPRE-pA | CTGGAGGGTGATCATTCTACA | Taitool |
| pAAV-CAG-DIO-MasterRNAi155(mAnxa2)#2-mCherry-WPRE-pA | AACTTCGATGCTGAGAGGGAT | Taitool |
| pAAV-CAG-DIO-MasterRNAi155(mAnxa2)#3-mCherry-WPRE-pA | TCACCATTGTCAACATCCTGA | Taitool |
| pAAV2-H1-shRNA(mTMEM215)#1-CAG-mCherry-WPRE-pA | GATACTGTTGCTACATCAACC | Taitool |
| pAAV2-H1-shRNA(mTMEM215)#2-CAG-mCherry-WPRE-pA | GGTGGGACCACGAGACAATAG | Taitool |
| pAAV2-H1-shRNA(mTMEM215)#3-CAG-mCherry-WPRE-pA | GGACAAGGAAGTGG T G GAACT | Taitool |

***Plasmid***

| **Description** | **P1（5'-3'）** | **P2（5'-3'）** | **Vendor or source** |
| --- | --- | --- | --- |
| TMEM215 | ACGGGCCCTCTAGACTCGAGCGCCACCatgcggcctgatgacattaac | TCATAAGGGTACATGGATCCgacgatggtctcgtggtcccacc | GENE |
| ANXA2 | CTCCCAGGTCCAACTGCACCTGCAGCTTGGAGGGTGATCACTCTAC | TGGATCCCGCCACCATGTCTACTGGATGCTTTGAACATTGAAACAG | GENE |
| ANXA2(del1-31aa) | CACACTGGACTAGTGGATCCCGCCACCatgaactttgatgctgagcggg | CCTTGTAGTCACTTAAGCTTGGgtcatctccaccacacaggtacag | GENE |
| ANXA2(del32-104aa) | CACACTGGACTAGTGGATCCCGCCACCATGTCTACTGTTCACGAAATCCTGTGCAAGCTCAGCTTGGAGGGTGATCACTCT | CCTTGTAGTCACTTAAGCTTGGgtcatctccaccacacaggtacag | GENE |
| ANXA2(del105-175aa) | CACACTGGACTAGTGGATCCCGCCACCatgtctactgttcacgaaatc | CCTTGTAGTCACTTAAGCTTGGgtcatctccaccacacaggtacag | GENE |
| ANXA2(del191-265aa) | CACACTGGACTAGTGGATCCCGCCACCatgtctactgttcacgaaatc | CCTTGTAGTCACTTAAGCTTGGgtcatctccaccacacaggtacag | GENE |
| ANXA2(del266-339aa) | CACACTGGACTAGTGGATCCCGCCACCatgtctactgttcacgaaatc | CCTTGTAGTCACTTAAGCTTGGgttctgaatgcactgaaccaggttcaggaaagcattttc | GENE |
| HSPA5 | TAGGGAGACCCAAGCTGGCTAGCATGAAGCTCTCCCTGGTGGCCGCGA | TGTTCGGGCCCAAGCTTGGTACCCAACTCATCTTTTTCTGCTGTATCC | GENE |

***Primers***

| **Description** | **Forward (F)** | **Reverse (R)** |
| --- | --- | --- |
| TMEM215 | AGGACTTCCTGCTGTGCTTT | CTTGTCCTTCTTGGGCTGTC |
| ANXA2 | GAGGCTGTGAAGGGCTATGA | TTCTTCTCCACAGCTTCACG |
| PLN | GCTGATGACGAGGAGGAGAA | CTTGTCCTCGATGATGTTGG |
| MEX3B | GAAGAGCGTGAACATGACCGAG | CCGTCACAACAAAGACAGGCTC |
| SFRP4 | TGAGGACTTTGACTTCCGCT | AGGCTTGTTGGTGTTGTTGA |
| SERPINA1 | AGGCTGTGCTGACCATCGAG | TTGTGGGATGAGGAGAGGCT |
| PENK | TGCTGCTGTGGAGCTGTTTA | CTCTGTGGGCAATGTTGAGG |
| BMP6 | CCCTTCATGGTGGCTTTCTT | GAGCGATTACGACTCTGGTTCTGTTGTC |
| ITGB3 | TGCTGGAGAAAGACAGAGGA | TCCTTCTGGGATGGTGATGT |
